# Identification and comparative analysis of the ovarian microRNAs of prolific and non-prolific goats during the follicular phase using high-throughput sequencing

**DOI:** 10.1038/s41598-017-02225-x

**Published:** 2017-05-15

**Authors:** Xiang-dong Zi, Jian-yuan Lu, Li Ma

**Affiliations:** Key-Laboratory for Animal Science of State Ethnic Affairs Commission, Southwest University for Nationalities, Chengdu, 610041 P.R. China

## Abstract

The kidding rate is one of the most important economic traits for goat production, but the genetic mechanism that is associated with ovulation rate is poorly understood. Recently, increasing evidence has suggested that microRNAs (miRNAs) influence ovarian biological processes. The present study provides the first comparison of the ovarian miRNAs of prolific Jintang black goats (JTGs) and non-prolific Tibetan goats (TBGs) during the follicular phase using RNA-Seq technology. We generated 11.19 million (M) and 11.34 M clean reads from the TBG and JTG libraries, respectively, from which a total of 389 known miRNAs were identified and 142 novel miRNAs were predicted. A total of 191 miRNAs were differentially expressed between the two breeds. Among the 10 most abundant miRNAs, miR-21-5p was defined as differentially expressed miRNA with a higher level in the JTG library than in the TBG library, but the other miRNAs were not different between the breeds. The predicted miRNA-targeted genes were further analyzed by Gene Ontology and KEGG pathway analyses. The results revealed that miR-21, miR-99a, miRNA-143, let-7f, miR-493 and miR-200b may affect follicular development. These findings will increase the current understanding of the role of ovarian miRNAs in the regulation of ovulation rate in goats.

## Introduction

MicroRNAs (miRNAs) are a family of small RNA molecules ~22 nucleotides (nt) in length that posttranscriptionally silence gene expression by binding to and/or cleaving the 3′-untranslated regions (3′-UTRs) of target mRNAs^1, 2^. These molecules function in various biological processes, including cell proliferation^3^, differentiation^4^, apoptosis^5^, tumourigenesis^6^, hormone secretion^7^, and metabolism^8^.

A growing body of evidence indicates a possible role for miRNAs in nearly all ovarian biological processes, including folliculogenesis, ovulation, luteal development, and regression, which are all widely recognized^9^. miR17-5p and let7b participate in angiogenesis by regulating the expression of the antiangiogenic factor tissue inhibitor of metalloproteinase 1. A lack of miR17-5p and let7b results in angiogenesis in the mouse corpus luteum (CL) and ultimately causes infertility^10^. A pair of miRNAs (miR-200 and miR-429) in the pituitary gland has been implicated in the regulation of female luteinizing hormone (LH) and fertility^11^. miR-26b increases the number of DNA breaks and promotes porcine granulose cellular (GC) apoptosis during follicular atresia by targeting the ataxia telangiectasia mutated (ATM) gene^12^. There is a temporal relationship between the up-regulation of miR-21 and the posttranscriptional regulation of programmed cell death 4 (PDCD4) during porcine oocyte maturation^13^. miR-34a regulates bovine luteal cell proliferation and function during the transition from developing to functional CL^14^. MiRNA-320 in the human follicular fluid is associated with embryo quality *in vivo* and affects mouse embryonic development *in vitro* ^15^. miR-125b can regulate the expression of genes crucial for embryo development and implantation in porcine endometrial luminal epithelial cells^16^. Recently, a large number of differentially expressed known and novel miRNAs that participate in the regulation of reproduction have been identified and characterized using high-throughput RNA sequencing (RNA-Seq) technology. miRNAs abundant in the extracellular vesicles (EVs) of small bovine follicles were associated with cell proliferation pathways, whereas miRNAs abundant in large follicles were related to inflammatory response pathways^17^. A large number of differentially expressed known and novel miRNAs have also been identified and comparatively analyzed in the ovaries of goats that are pregnant vs. non-pregnant^18^ or have high vs. low kidding rates^19–21^ and in pigs with high vs. low litter sizes^22^. For example, Miao *et al*. identified 30 differentially expressed miRNAs in the ovaries of highly prolific Jining Grey goats and the less prolific Laiwu Black goats, and most of these miRNAs had defined homologs that were key miRNAs related to reproduction^21^.

Jintang black goats (JTGs) represent a local Chinese breed famous for its high fecundity (average kidding rate = 242%) and meat performance. While these animals have been used in many areas of China to increase the fecundity and meat performance of local breeds, no direct evidence has been reported for the involvement of mutations with large effects on prolificacy^23^. Tibetan goats (TBGs) (*Capra hircus*), with a population of 7.2 million, are single-birth breeds characterized by their adaptation to the cold, hypoxic ecological conditions in the Qinghai-Tibet Plateau. These animals provide their owners with meat where few other animals (except for yaks and Tibetan sheep) survive, although their production traits are inferior to those of improved goat breeds. In the present study, we summarize the first characterization and investigation of the miRNA expression profiles in the ovaries of prolific JTGs and non-prolific TBGs during the follicular phase using RNA-Seq technology. The results will increase the current understanding of the role of ovarian miRNAs in the regulation of ovulation rate in goats.

## Results

### Sequence analysis and mapping of small RNA reads

**Figure 1:**
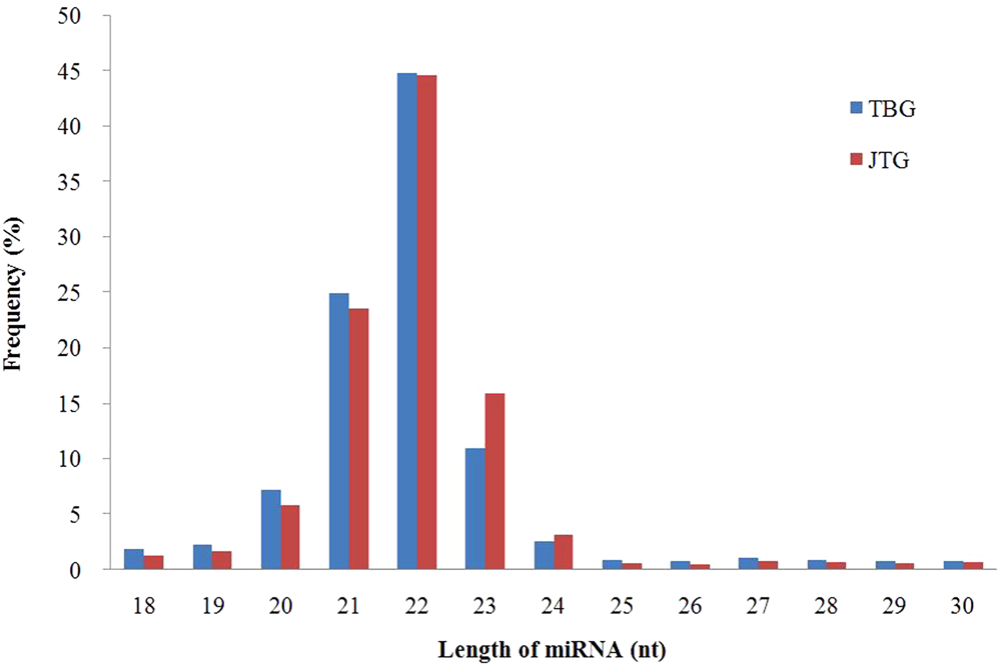
Sequence length distribution in the non-prolific Tibetan goat (TBG) and the prolific Jintang black goat (JTB) ovarian libraries.

To identify and characterize miRNAs during the follicular phase in goat ovaries, two small RNA libraries, non-prolific TBGs and prolific JTGs, were constructed. A total of approximately 14 million raw reads were obtained from each library by high-throughput sequencing. After eliminating adaptor and low-quality reads, we finally obtained 11,192,277 (78.08% of total) and 11,341,703 (81.75%) clean reads from TBGs and JTGs for further analysis, respectively (Table 1). The sequence length distributions ranging from 18 to 30 nt were similar between the two libraries, and the majority of small RNA molecules ranged from 20 to 24 nt in length (Fig. 1). There was a peak at 22 nt in length, the typical size of Dicer-derived products^24^, which accounted for 44.89% and 44.65% of the total sequence reads in the TBG and JTG libraries, respectively.

**Table 1:** Classification of total small RNA tags by RNA-Seq in Tibetan goat (TBG) and Jintang black goat (JTG) ovary libraries.

	TBG		JTG	
	No	%	No	%
Total_reads	14,333,506		13,874,032	
Filter having N reads	5,913	0.04	5,524	0.04
Smaller than 18 nt	1,072,828	7.48	805,444	5.81
Larger than 30 nt	2,062,488	14.39	1,721,361	12.41
Clean reads	11,192,277	78.08	11,341,703	81.75

**Figure 2:**
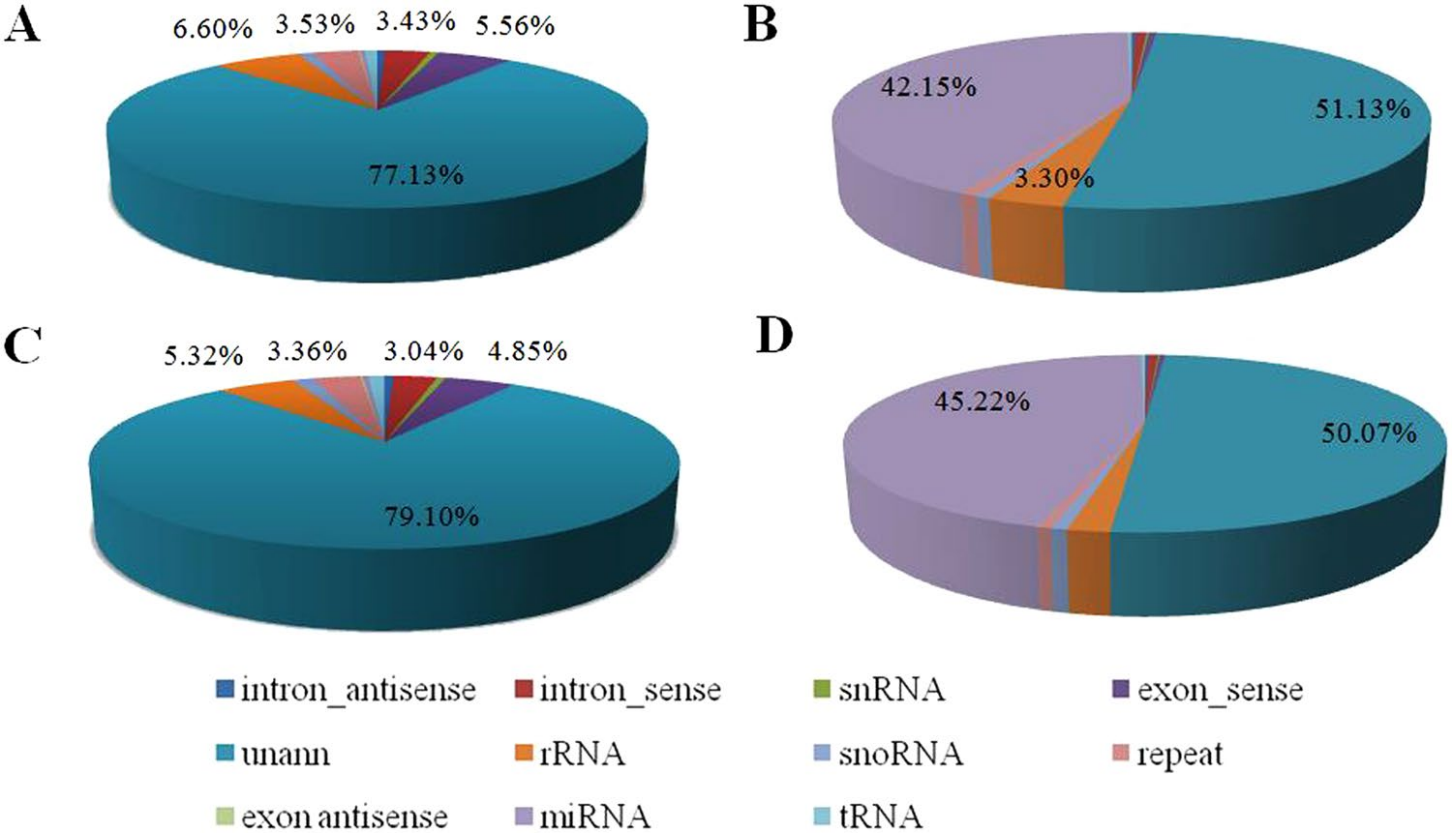
Composition of small RNA classes of the Solexa sequencing results. (**A**) Composition of unique sequences in the non-prolific Tibetan goat (TBG) library. (**B**) Composition of clean reads in the non-prolific TBG library. (**C**) Composition of unique sequences in the prolific Jintang black goat (JTB) library. (**D**) Composition of clean reads in the prolific JTB library.

All clean reads were annotated and classified by alignment against the Rfam11.0 and GenBank databases. Each unique small RNA was verified to map to only one annotation using the priority rule^24^. The compositions of the small RNA classes of the Solexa sequencing results are shown in Fig. 2 and Supplementary Table S1. In the present study, the total miRNA accounted for 51.13% and 50.07%, whereas the total rRNA accounted for only 3.30% and 1.80% in the TBG and JTG libraries, respectively, indicating that the collected ovary samples were of high quality. A total of 5,362,108 (47.91%) and 5,595,846 (49.34%) clean reads in the TBG and JTG libraries, respectively, were mapped to the goat genome.

### Identification of conserved miRNAs

To identify conserved miRNAs in hircine ovaries, all clean reads were aligned to the miRNA precursor/mature miRNAs of *Capra hircus* in the miRBase 21.0 database (http://www.mirbase.org/ftp.shtml). We then obtained the family groups, expression levels and sequences of the miRNAs (Supplementary Table S2). In summary, we identified 388 and 389 conserved miRNAs in the TBG and JTG libraries, respectively. Among these molecules, 379 were co-expressed, and nine and 10 miRNAs were specifically expressed in the non-prolific TBG and prolific JTG libraries, respectively. The levels of these specifically expressed miRNAs were extremely low (under 5 read counts).

### Novel miRNA prediction

The hairpin structures of the miRNA precursors were used to predict novel miRNAs using Mireap v0.2. These novel miRNAs were then mapped to the goat genome. In total, we detected 142 novel miRNAs, of which 61 were co-expressed, and 33 and 48 were specific miRNAs in the TBG and JTG libraries, respectively (Supplementary Table S3). Six novel miRNAs with expression higher than 1,000 read counts were the same between the two libraries (Table 2).

**Table 2:** Novel miRNAs in ovarian libraries with expression higher than 1,000 read counts.

miR_name	TBG	JTG	log2 ratio (JTG/TBG) normalized	p-value	q-value	sig-lable
Novel-miR-8	6,803	9,244	0.2517	1.19E-40	1.89E-39	No
Novel-miR-20	2,827	2,785	−0.2122	4.67E-09	9.87E-09	No
Novel-miR-93	2,701	3,121	0.0179	0.6146	0.1522	No
Novel-miR-94	2,352	2,397	−0.1633	4.02E-05	4.25E-05	No
Novel-miR-77	2,160	2,336	−0.0776	0.0583	0.0183	No
Novel-miR-72	1,162	1,001	−0.4058	2.29E-11	7.25E-11	No

### Differential miRNA expression between the two goat breeds

**Figure 3:**
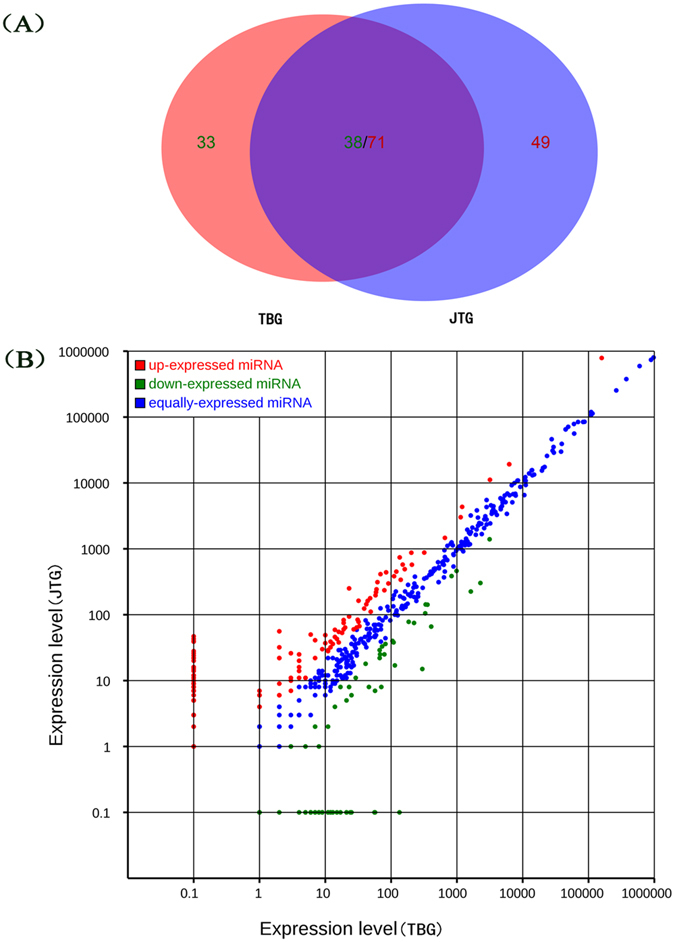
Differentially expressed miRNAs between the non-prolific Tibetan goat (TBG) and prolific Jintang black goat (JTG) ovary libraries. (**A**) Venn diagram displaying the distribution of 191 differentially expressed unique miRNAs (fold-change ≥ 1.0 or ≤ −1.0 and q*-*value < 0.05) in the non-prolific TBG (left circle) and prolific JTG libraries (right circle). The overlapping region indicates co-expressed unique miRNAs. (**B**) In the scatter plot of differentially expressed miRNAs, the red points indicate miRNAs with ratios > 2; the blue points indicate miRNAs with 1/2 < ratios ≤ 2; and the green points indicate miRNAs with ratios ≤ 1/2. Ratio = normalized expression of JTGs/normalized expression of TBGs.

Differential miRNA expression analysis of the two libraries was performed using the DEGseq (2010) R package^25^. The results are shown in Fig. 3 and Supplementary Table S4. Analysis of the sequencing data resulted in the identification of 191 unique miRNAs, which were significantly differentially expressed in different libraries at a threshold of fold-change ≥ 1.0 or ≤−1.0 and a q*-*value < 0.05. Among these 191 unique miRNAs, 120 (49 prolific-specific, 71 co-expressed) and 71 (33 non-prolific-specific, 38 co-expressed) unique miRNAs were up- and down-regulated, respectively, in the prolific JTG library compared to expression in the non-prolific TBG library. The top 10 most significantly differentially expressed miRNAs were miR-21, miR-199b, miR-199c, miR-127, miR-200a, miR-379, miR-200b, miR-204, miR-411a, and miR-493. The 10 most abundant miRNAs (miR-99a-5p, miR-148a-3p, miR-143-3p, miR-10b-5p, miR-26a-5p, miR-21-5p, miR-125b-5p, miR-27b-3p, let-7f-5p, and miR-101-3p) were the same in the two libraries, of which miR-21-5p was defined as differentially expressed miRNA with a higher level in the prolific library than in the non-prolific library, but the remaining nine miRNAs did not meet the criteria of differentially expressed miRNA between the breeds (Table 3).

**Table 3:** Comparison of the 10 most abundant conserved miRNAs in the ovarian libraries of the two goat breeds.

miR_name	TBG	JTG	log2 ratio (JTG/TBG), normalized	p-value	q-value	sig-lable
miR-99a-5p	973,391	802,348	−0.4129	0	0	No
miR-21-5p	158,549	788,052	2.1793	0	0	Yes
miR-148a-3p	891,044	739,216	−0.4036	0	0	No
miR-143-3p	597,073	594,445	−0.1404	0	0	No
miR-10b-5p	375,466	376,792	−0.1290	0	0	No
miR-26a-5p	264,052	253,691	−0.1918	0	0	No
miR-27b-3p	109,607	119,279	−0.0121	0.0427	0.0249	No
miR-125b-5p	114,899	113,234	−0.1551	6.32E-149	2.77E-148	No
let-7f-5p	108,566	106,187	−0.1660	3.25E-160	1.59E-159	No
miR-101-3p	86,961	847,64	−0.1710	1.37E-135	5.60E-135	No

### Target gene prediction for miRNAs

We used miRanda v3.3a, TargetScan v61, and PITA v6. to predict potential target genes regulated by miRNAs. Given the high false positive rates for miRNA target prediction, we only identified those potential target genes predicted by all three methods. In the non-prolific TBG library, a total of 29,489 target genes were predicted from 388 conserved miRNAs, and 29,260 target genes were predicted from 94 novel miRNAs. In the prolific JTG library, a total of 29,489 target genes were predicted from the 389 conserved miRNAs, and 29,314 target genes were predicted from 109 novel miRNAs (Supplementary Table S5).

### Gene ontology and KEGG pathway analysis of target genes

**Figure 4:**
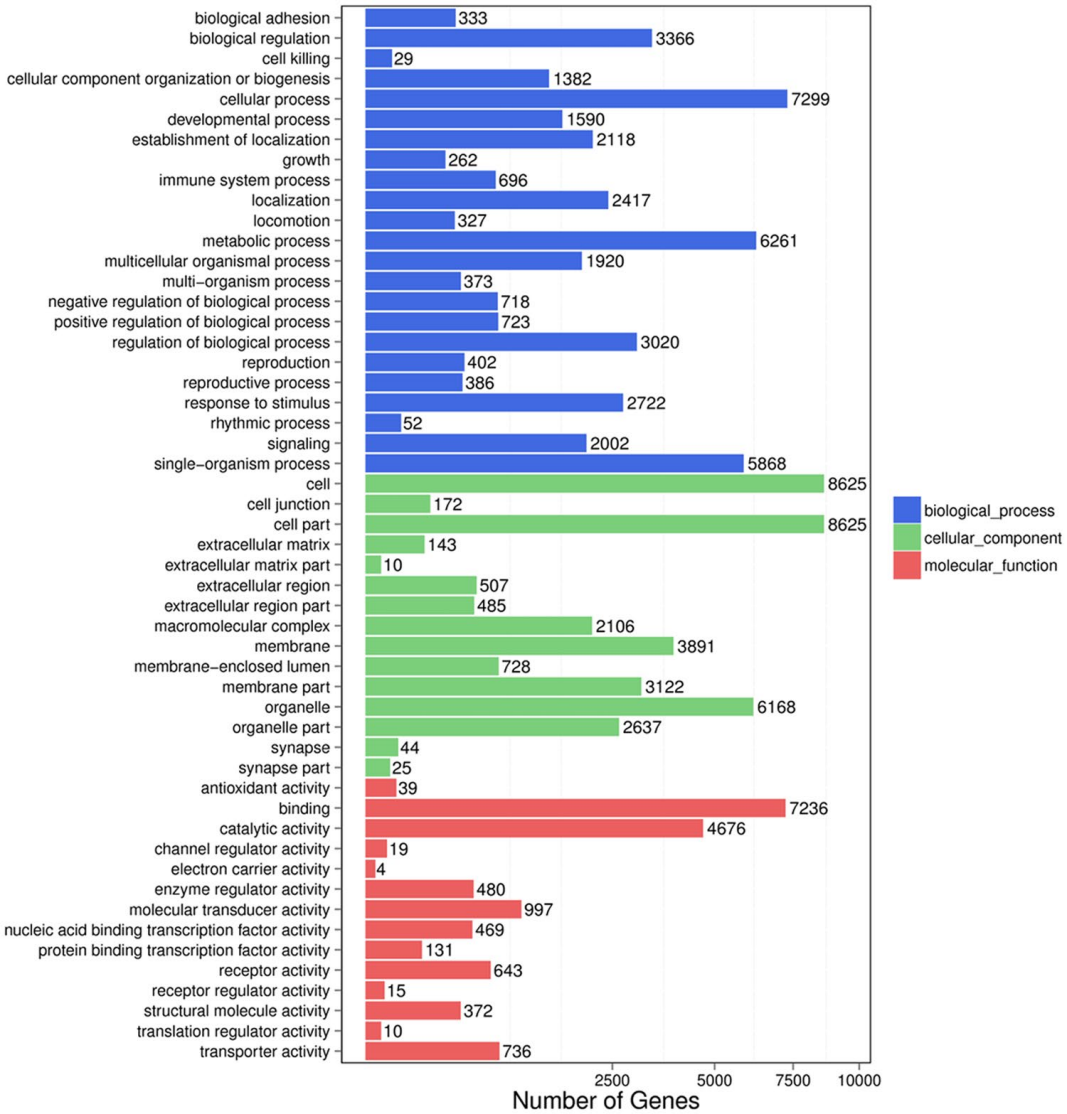
GO functional classification of all differentially expressed genes (DEGs) predicted from the known miRNAs. The GO distributions of the DEGs in the ovaries of the non-prolific Tibetan goats (TBG) vs. the prolific Jintang black goats (JTG) were classified into three categories: cellular components (15 subcategories), molecular functions (14 subcategories), and biological processes (23 subcategories).

Gene Ontology (GO) of the predicted miRNA target genes was performed by searching against the InterPro database^26^ using InterProScan^27^. For all predicted miRNA target genes in these two libraries, a total of 10,793 target genes were mapped to the GO terms of *cellular components*, 10,486 target genes were associated with the GO terms of *molecular function*, and 10,019 target genes were related to the *biological processes* of the GO terms in the prolific JTG and non-prolific TBG libraries. Compared to the reference gene background, GO terms were neither significantly enriched for these two libraries based on the above three ontologies in GO nor significantly enriched for differentially expressed genes (DEGs) predicted from the known miRNAs (Fig. 4). However, 12 GO terms were significantly (q*-*value < 0.05) enriched for DEGs predicted from the novel miRNAs based on the molecular function (Supplementary Table S6), including *transferase activity*, *metal ion binding*, *catalytic activity*, *small molecule binding*, *nucleotide binding*, *nucleoside phosphate binding*, *purine nucleotide binding*, and others.

The Kyoto Encyclopedia of Genes and Genomes (KEGG) pathways of target genes were assigned using KAAS webserver^28^. KEGG pathway annotation revealed that 22,885 background genes were annotated for 310 biological functions. There were no significantly (q-value > 0.05) enriched KEGG pathways. The top five pathways were *olfactory transduction, regulation of actin cytoskeleton, pathways in cancer, biosynthesis of secondary metabolite*, and *focal adhesion* in prolific JTGs compared to *metabolic pathways, olfactory transduction, regulation of actin cytoskeleton, pathways in cancer*, and *biosynthesis of secondary metabolite* in non-prolific TBGs. The DEGs also participated in 310 pathways, but there were no significantly enriched KEGG pathways. The top five most enriched pathways were *olfactory transduction, regulation of actin cytoskeleton, biosynthesis of secondary metabolites, focal adhesion*, and *endocytosis*.

### Validation of sequencing results by quantitative real-time RT-PCR (RT-qPCR)

**Figure 5:**
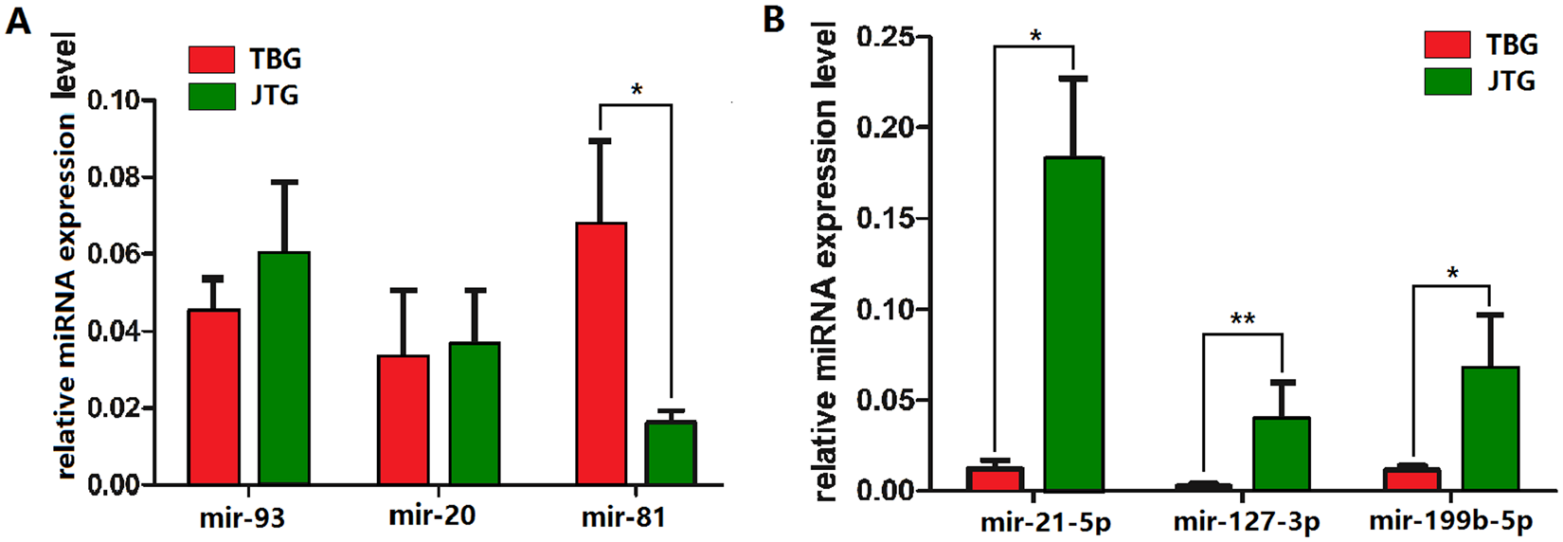
Validation of sequencing results by RT-qPCR. (**A**) A bar graph showing novel miRNAs, and (**B**) a bar graph showing known miRNAs. Error bars indicate mean ± SE (*n* = 5 per breed), *p < 0.05, **p < 0.01.

The expression levels of six randomly selected miRNAs, including three known miRNAs (miR-21-5p, miR-127-3p and miR-199b-5p) and three novel miRNAs (miR-93, miR-20 and miR-81), were verified in the ovaries of prolific JTGs (*n* = 5) and non-prolific TBGs (*n* = 5) using RT-qPCR. Solexa sequencing results indicated that four of these genes were differentially expressed. RT-qPCR analysis demonstrated that the relative expression levels of these six selected miRNAs were consistent with the RNA-Seq results (Fig. 5).

## Discussion

The kidding rate is fundamental to goat production, but the genetic mechanism underlying ovulation rates is poorly understood, which largely limits the improvement of this trait through genetic selection^29, 30^. Various mutations influencing ovulation rate and litter size in sheep provide additional opportunities to rapidly adjust genetic potentials, but these mutations require careful breeding management^31^. Comparable major genes have not yet been identified in goats^29, 30^. In the present study, we provided the first characterization and investigation of the ovarian miRNA expression profiles of the prolific JTGs and non-prolific TBGs during the follicular phase using Solexa sequencing technology.

We harvested intact ovaries from prolific and non-prolific goats during the follicular phase and completely homogenized these samples for RNA extraction and thus ensured that the miRNA-Seq results were representative of the ovarian miRNAome. In the present study, the length distribution showed that more than 90% of the small RNA sequences were primarily distributed in the 19- to 24-nt range in both libraries. The dominant size of small RNAs in the ovary was 22 nt, followed by 21- and 23-nt sequences. These results are consistent with typical Dicer-processed small RNA products with a known 19- to 24-nt range for miRNAs^24^. These results indicate that the high-throughput sequencing data were highly enriched for small RNA sequences. These sequencing data are consistent with previous findings in mice^32^, pigs^22^, goats^18–21^, and chickens^33^, but vary among bovine ovaries, where the 20-nt size was the most abundant, followed by 22 nt^34, 35^, likely reflecting species differences.

We identified total of 388 and 389 conserved miRNAs in the non-prolific TBG and prolific JTG libraries, respectively. The 10 most highly expressed miRNAs (miR-99a, miR-148a, miR-143, miR-10b, miR-26a, miR-21, miR-125b, miR-27b, let-7f, and miR-101) identified in the present study were also highly expressed in the ovaries of goats^18, 20^, pigs^22^, and other animal species as reviewed by Li *et al*.^9^. Six of these miRNAs (miR-99a, miR-143, miR-10b, miR-26a, miR-125b, and let-7f) are specifically or predominantly expressed in bovine ovaries compared to the somatic tissue pools (e.g. hypothalamus, liver, heart, lung, kidney, etc.) assessed by RT-qPCR^36^. Most of these miRNAs are also abundantly present in human ovaries^37^. The ovary contains oocytes and multiple somatic cell types such as GCs and theca cells. The expression and function of miRNAs are associated with different cell types. Various miRNAs were expressed in GCs (miR-143, miR-125b, let-7 family, miR-21, miR-10b, miR-378, etc.)^38–40^, theca cells (mir-24, miR-222 and miR-378)^40, 41^, and oocytes (miR-10a, miR-10b, miR-100, let-7 family, etc.)^36, 42, 43^. All of these miRNAs were also highly expressed in goat ovaries in the present study, and they may play a housekeeping role in the ovary, independent of species.

There is increasing evidence to suggest that these ovarian specifically or predominantly expressed miRNAs may play important roles in normal physical development and basic reproductive activities. miR-99a has been shown to be one of the most predominant miRNA populations in mammalian ovaries^18, 22, 41^; it induces G1-phase cell cycle arrest and suppresses tumourigenicity^44^, which may play critical roles in normal ovarian functions. miR-125b suppresses proapoptotic protein expression^45^, and androgens attenuate follicular atresia through nuclear and extranuclear signalling pathways by enhancing miR-125b expression, which in turn suppresses proapoptotic protein expression^46^. The members of the let-7 family play important roles in cell fate determination and are associated with regulating housekeeping genes during ovarian development^47^. Target prediction indicated that miR-143 might bind to the Frizzled-6 and Frizzled-3 receptor genes in the Wnt signalling pathway, through which it affects the binding of Wnt-4 to its receptor^18^. The Wnt-4 gene, one of the most important members of the Wnt family, may regulate the function of ovarian GCs and luteal cells by binding to specific members of the Frizzled receptor family^48, 49^. MiR-26a is abundant in the ovary but not in the testis. MiR-26a promotes ovarian cancer proliferation and tumorigenesis^50^, which may be related to follicle degeneration at various stages of follicular growth and development in mammalian ovaries.

In total, 142 novel miRNAs were detected from the prolific JTG and non-prolific TBG libraries, which is distinctly higher than the amount predicted in the ovaries of polytocous compared with monotocous goats^19, 20^ and pigs with high and low litter sizes^22^. Analysis of the sequencing data resulted in the identification of 191 unique miRNAs, which were significantly differentially expressed between the prolific JTG and non-prolific TBG libraries. The top 10 most significantly differentially expressed miRNAs (miR-21, miR-199b, miR-199c, miR-127, miR-200a, miR-379, miR-200b, miR-204, miR-411a, miR-493) were analysed. miR-21 plays an important role in the posttranscriptional regulation of transcripts involved in the prevention of apoptosis of murine GCs. The apoptosis increased and ovulation rate decreased in locked nucleic acid 21 (LNA-21)-treated murine ovaries^51^. Hasuwa *et al*. described anovulation and infertility in female mice lacking the microRNAs miR-200b and miR-429. Both miRNAs are strongly expressed in the pituitary gland, where they suppress the expression of the transcriptional repressor ZEB1. Eliminating these miRNAs, in turn, inhibits LH synthesis by repressing the transcription of its β-subunit gene, thereby reducing the serum LH concentration, impairing LH surge, and repressing ovulation^11^. These results reveal roles for miR-200b and miR-429, and their target gene *Zeb1*, in the regulation of mammalian reproduction. Thus, the hypothalamo-pituitary-ovarian axis required miR-200b and miR-429 to support ovulation^11^. miR-127 has a tumour suppressor function in cancer pathways^52^. The expression level of miR-493-3p was also higher in ovaries of polytocous compared with monotocous goats^19, 21^. Booroola was the first major gene (FecB) that has been reported to increase ovulation rate in sheep in the early 1980’s^53^. Twenty years later, sheep carrying the FecB have been demonstrated to have a mutation (Q249R) in the coding sequence of the Bone Morphogenetic Protein Receptor-type 1B (BMPR1B) responsible for the hyperprolific phenotype^54–56^. Recently, the BMPR1B gene has been reported to be regulated by chi-miR-493-3p alone^21^. RT-qPCR was performed to analyse the expression profiles of six randomly selected miRNAs (including three known miRNAs and three novel miRNAs) in prolific and non-prolific goat ovaries, and the results were consistent with the sequencing analysis, indicating the good reliability and reproducibility of transcript abundance assayed using RNA-seq.

*Olfactory transduction, actin cytoskeleton regulation, secondary metabolite biosynthesis, focal adhesion*, and *endocytosis* were the major pathways involving DEGs. *Focal adhesion and actin cytoskeleton regulation* pathways were also enriched in the ovary transcriptomes of other goat breeds^57, 58^. Focal adhesion plays essential roles in important biological processes, including cell motility, cell proliferation, cell differentiation, regulation of gene expression and cell survival^59^. Numerous small molecular substances that regulate oocyte growth and development are transported through this connection^60^. Recent studies have shown that the actin cytoskeleton also has an important function in the early development of oocyte maturation. The regulation of the dynamic actin cytoskeleton includes signalling to the cytoskeleton, which can lead to diverse effects on cellular activity, such as changes in cell shape, migration and proliferation^61^. The cell prepares for meiosis when actin cytoskeleton dynamics change appropriately^62, 63^. Therefore, these signalling pathways are closely associated with follicle development.

In conclusion, we identified 379 co-expressed miRNAs, and 9 and 10 specific miRNAs in the ovaries of the non-prolific TBGs and prolific JTGs, respectively, during the follicular phase. We also obtained 191 differentially expressed miRNAs in the ovaries of the two distinctive breeds, of which 120 were up-regulated and 71 were down-regulated in the prolific JTGs compared to the non-prolific TBGs. 142 novel miRNAs were predicted, of which 61 were co-expressed, and 33 and 48 were specifically expressed in the TBG and JTG libraries, respectively. miR-21, miR-99a, miRNA-143, let-7f, miR-493 and miR-200b may play important roles in follicular development. The results of the present study will increase the current understanding of the role of ovarian miRNAs in the regulation of ovulation rate in goats.

## Materials and Methods

### Ethics statement

All experimental procedures and sample collections were conducted in accordance with the Regulations for the Administration of Affairs Concerning Experimental Animals (Ministry of Science and Technology, China; revised in August 2011) and were approved by the Institutional Animal Care and Use Committee of Southwest University for Nationalities, Chengdu, China.

### Animals and ovary collection

A total of twelve healthy female goats were used in the present study from two breeds, i.e., the extreme high prolific breed of Jintang black goat (JTG: *n* = 6), and the non-prolific breed of Tibetan goat (TBG: *n* = 6). To reduce the effects of age, parity and stage of oestrus on the RNA-Seq results, all animals selected had the same age (4-yr) and parity (four parities), and oestrus cycles were synchronized. The JTGs had a history of successive multiple births (≥triplet births), and the TBGs had a history of successive single birth. All does were treated for 13 days using Controlled Internal Drug Release Devices (CIDR) containing 300 mg progesterone (Eazi-Breed CIDR, InterAg, Hamilton, New Zealand) to synchronize oestrus. All does were intramuscularly injected with 3.75 mg of the PGF_2α_ analogue Luprostiol (0.5 mL Prosolvinl, Intervet) at 24 h prior to CIDR removal. Five goats showing the most synchronized time of oestrus in each breed were subsequently slaughtered at 40 h (follicular phase) after CIDR removal. The intact ovaries were rapidly harvested and immediately frozen in liquid nitrogen, followed by storage at −80 °C for the subsequent generation of small RNA libraries.

### Small RNA library construction and sequencing

The ovaries were completely ground, and total RNA was extracted using TRIzol (Invitrogen Inc., California, USA). RNA purity was assessed using a NanoPhotometer^®^ spectrophotometer (IMPLEN, CA, USA). The RNA concentration was measured using the Qubit^®^ RNA Assay Kit in Qubit^®^ 2.0 Fluorometer (Life Technologies, CA, USA). RNA integrity was assessed on a Bioanalyzer 2100 (Agilent, USA) using an RNA Nano 6000 kit (Agilent Technologies, Palo Alto, CA). One and half micrograms of total RNA per sample was used as input material for the creation of two small RNA libraries. Sequencing libraries were generated using a NEBNext^®^Ultra^TM^ small RNA Sample Library Prep Kit for Illumina^®^ (NEB, USA) following the manufacturer’s recommendations, and index codes were added to identify the sequences from each sample. Briefly, the RNA was reversed transcribed into cDNA after ligation of the multiplex 3′SR Adaptor, hybridization of the reverse transcription primer, and ligation of the multiplex 5′SR Adaptor. RNA libraries were amplified by PCR using Illumina-compatible index primers. The amplified libraries were resolved on a 6% native polyacrylamide gel (Bio-Rad, CA). Finally, the library quality was assessed on a Bioanalyzer 2100 (Agilent), and the libraries were quantified by real-time qPCR using KAPA Library Quantification Kits (KAPA Biosystems, Wilmington, MA). The clustering of the index-coded samples was performed on a cBot Cluster Generation System using a TruSeq PE Cluster Kit v4-cBot-HS (Illumina) according to the manufacturer’s instructions. After cluster generation, the library preparations were sequenced on an Illumina HiSeq 2500 platform, and paired-end reads were generated.

### Sequence analysis

The clean reads were obtained by removing reads containing with 5′ adaptor contaminants or poly-A, reads without 3′ adaptor or insert tag, shorter than 18 nt, or low quality reads from the raw data. Subsequently, we obtained the length, distribution and counts of these clean reads. In the alignment and annotation steps, some small RNA tags may be mapped to more than one category. To ensure that unique small RNAs mapped to only one annotation, we followed the following priority rule: rRNAetc (GenBank > Rfam) > known miRNA > piRNA > repeat > exon > intron^24^. All clean reads were mapped to the ncRNA database from Rfam 11.0 (http://rfam.janelia.org) and GenBank databases using a BLASTN algorithm. They were also mapped to the goat genome (http://goat.kiz.ac.cn/GGD/download.htm)^64^ using SOAP v2.20 to analyze their expression and distribution in the goat genome. The clean reads were aligned to the miRNA precursor/mature miRNA of *Capra hircus* in miRBase 21.0 (http://www.mirbase.org/ftp.shtml) to identify the sequences and counts of miRNA families observed in the samples. The characteristics of hairpin structures of miRNA precursors can be used to predict novel miRNAs. Mireap v0.2 (http://sourceforge.net/projects/mireap/) was used to analyze the unannotated reads.

### Analysis of differential miRNA expression between TBGs and JTBs

To compare the differential miRNA expression between TBG and JTG libraries, the clean reads from each miRNA were normalized using the following formula: normalized expression = mapped read count/total number of reads × 1,000,000. If the normalized read count of a given miRNA is zero, then the expression value is set to 0.01 for further analysis. Differential expression analysis of two samples was performed using the DEGseq (2010) R package^25^. The p*-*value was adjusted using the q-value^65^, and the q*-*value was set as the default threshold for significant differential expression. The differentially expressed miRNAs were screened with a threshold of fold-change ≥ 1.0 or  ≤ −1.0 (the log2 JTG/TBG) and a q*-*value < 0.05.

### Predictions of target genes

The 3′-UTR sequences of the goat RefSeq genes (http://goat.kiz.ac.cn/GGD/download.htm) were downloaded and used to identify miRNA targets using three different computational approaches: miRanda v3.3a, TargetScan vert_61, and PITA v6. Only the 3′-UTR targets identified by all three approaches were further considered.

### Annotation of miRNA targets

Gene Ontology (GO) and Kyoto Encyclopaedia of Genes and Genomes (KEGG) enrichment analysis of the predicted miRNA target genes were performed as previously described^66^. First, the GO annotation was performed by searching against the InterPro database2^6^ using InterProScan^27^. KEGG pathways were assigned using the KAAS webserver^28^. We used a hypergeometric test to identify the significantly enriched GO terms or pathways in DEGs compared to the genome background. Multiple tests were adjusted using the false discovery rate (FDR) method^67^. The adjusted p-value ≤ 0.05 was set as the significant threshold.

### Validation of sequencing results using real-time RT-PCR

Six miRNAs were selected for validation using quantitative real-time PCR (RT-qPCR). Primers for RT-qPCR (Supplementary Table S7) were designed with Primer3web software (http://primer3.ut.ee/). RT-qPCRs were analysed in a BioRad CFX96 Real-Time PCR System with SYBR Green PCR Master Mix (Bio-Rad SsoFast, USA) and were amplified with 2 μl of cDNA template, 10 μl of 2 × SYBR Green PCR SuperMix, and 0.5 μl of each primer (10 nM) in a final volume of 20 μl by adding MilliQ water. The amplification programme consisted of 95 °C for 10 min, followed by 40 cycles at 95 °C for 15 s and 60 °C for 15 s. The fluorescent products were detected in the last step of each cycle. A melting curve analysis was performed at the end of 40 cycles to ensure proper amplification of target fragments. All reactions were performed in three replicates, and β-actin was used as the internal control. The relative expression level of each miRNA was caculated using the 2^−ΔΔCt^ method. The data are indicated as the means ± SE (*n = *5). The significance of the expression in two samples was calculated using a two sample *t*-test in SPSS statistical software (Version17.0, Chicago, IL, USA). The difference was considered as significant when p < 0.05.

## Electronic supplementary material

Supplementary Information
