# Supplementary material for: Identification and comparative analysis of the ovarian microRNAs of prolific and non-prolific goats during the follicular phase using high-throughput sequencing
Source: Sci Rep. 2017 May 15;7:1921. doi: 10.1038/s41598-017-02225-x (PMC5432505; doi:10.1038/s41598-017-02225-x)
Supplement: Supplementary file 1 — Supplementary Information [file 41598_2017_2225_MOESM1_ESM.pdf]

Supplementary Information

Identification and comparative analysis of the ovarian microRNAs of prolific and non-prolific goats during the follicular phase using high-throughput sequencing

Xiang-dong Zi[*], Jian-yuan Lu & Li Ma

Key-Laboratory for Animal Science of State Ethnic Affairs Commission, Southwest University for Nationalities, Chengdu 610041, PR China.

***Correspondence:** zixd2000@yahoo.com

Key-Laboratory for Animal Science of State Ethnic Affairs Commission, Southwest University for Nationalities, Chengdu 610041, PR China

Supplementary Table S1

Composition of small RNA classes of the Solexa in the ovaries of Tibetan goat (**a**) and Jintang black goat (**b**)

(a) Non-prolific Tibetan goat

Category	unique reads	% of unique reads	total reads	% of total reads
clean reads	881509	100.00	11192277	100.00
match genome	177165	20.10	5362108	47.91
exon_antisense	967	0.11	1242	0.01
exon_sense	49011	5.56	59091	0.53
intron_antisense	5006	0.57	27040	0.24
intron_sense	30275	3.43	88254	0.79
miRNA	3187	0.36	4717201	42.15
rRNA	58191	6.60	369523	3.30
repeat	31148	3.53	86164	0.77
snRNA	5798	0.66	18882	0.17
snoRNA	9573	1.09	71927	0.64
tRNA	8475	0.96	30762	0.27
unann	679878	77.13	5722191	51.13

(b) Prolific Jintang black goat

Category	unique reads	% of unique reads	total reads	% of total reads
clean reads	719416	100.00	11341703	100.00
match genome	137731	19.14	5595846	49.34
exon_antisense	869	0.12	1126	0.01
exon_sense	34901	4.85	41316	0.36
intron_antisense	4601	0.64	22524	0.20
intron_sense	21856	3.04	78205	0.69
miRNA	3307	0.46	5128858	45.22
rRNA	38290	5.32	204053	1.80
repeat	24180	3.36	62403	0.55
snRNA	4425	0.62	12741	0.11
snoRNA	10087	1.40	81342	0.72
tRNA	7865	1.09	30609	0.27
unann	569035	79.10	5678526	50.07

Supplementary Table S2

Conserved miRNAs in the ovaries of Tibetan goat (**a**) and Jintang black goat (**b**) during follicular phase

(a) Non-prolific Tibetan goat

miRNA	Counts	Sequence
chi-miR-99a-5p	973391	AACCCGTAGATCCGATCTTGT
chi-miR-148a-3p	891044	TCAGTGCACTACAGAACTTTGT
chi-miR-143-3p	597073	TGAGATGAAGCACTGTAGCTCG
chi-miR-10b-5p	375466	TACCCTGTAGAACCGAATTTGT
chi-miR-26a-5p	264052	TTCAAGTAATCCAGGATAGGCT
chi-miR-21-5p	158549	TAGCTTATCAGACTGATGTTGAC
chi-miR-125b-5p	114899	TCCCTGAGACCCTAACTTGT
chi-miR-27b-3p	109607	TTCACAGTGGCTAAGTTCTGC
chi-let-7f-5p	108566	TGAGGTAGTAGATTGTATAGTT
chi-miR-101-3p	86961	TACAGTACTGTGATAACTGA
chi-miR-100-5p	82633	AACCCGTAGATCCGAACTTGT
chi-let-7i-5p	69326	TGAGGTAGTAGTTTGTGCTGTT
chi-let-7g-5p	60544	TGAGGTAGTAGTTTGTACAGTT
chi-miR-145-5p	60065	GTCCAGTTTTCCCAGGAATCCCT
chi-miR-126-3p	49103	TCGTACCGTGAGTAATAATGC
chi-miR-199a-3p	44884	ACAGTAGTCTGCACATTGGTT
chi-miR-199b-3p	44881	ACAGTAGTCTGCACATTGGTT
chi-miR-199c-3p	44881	ACAGTAGTCTGCACATTGGTT
chi-let-7a-5p	39199	TGAGGTAGTAGGTTGTATAGTT
chi-miR-125a-5p	38255	TCCCTGAGACCCTTTAACCTGT
chi-let-7c-5p	29712	TGAGGTAGTAGGTTGTATGGTT
chi-miR-30a-5p	29399	TGTAAACATCCTCGACTGGAAGCT
chi-miR-140-3p	28454	ACCACAGGGTAGAACCACGGAC
chi-miR-199a-5p	27543	CCCAGTGTTCAGACTACCTGTTC
chi-let-7b-5p	23396	TGAGGTAGTAGGTTGTGTGGTT
chi-miR-29a-3p	21523	TAGCACCATCTGAAATCGGTT
chi-miR-99b-5p	20313	CACCCGTAGAACCGACCTTGCG
chi-miR-26b-5p	19589	TTCAAGTAATTCAGGATAGGTT
chi-miR-191-5p	14900	CAACGGAATCCCAAAAGCAGCT
chi-miR-10a-5p	14037	TACCCTGTAGATCCGAATTTGT
chi-miR-25-3p	13875	CATTGCACTTGTCTCGGTCTGA
chi-miR-532-5p	13684	CATGCCTTGAGTGTAGGACCGT
chi-miR-92a-3p	12497	TATTGCACTTGTCCCGGCCTGT
chi-miR-22-3p	11104	AAGCTGCCAGTTGAAGAAC
chi-miR-24-3p	11089	TGGCTCAGTTCAGCAGGAAC

chi-miR-92b	10966	TATTGCACTTGTCCCGGCCTGT
chi-miR-151-3p	10642	CTAGACTGAAGCTCCTTGAGG
chi-miR-542-3p	10575	TGTGACAGATTGATAACTGA
chi-miR-202-5p	9197	TTCCTATGCATATACTTCTTT
chi-miR-455-5p	8469	TATGTGCCTTTGGACTACATCG
chi-miR-16a-5p	8322	TAGCAGCACGTAAATATTGGAG
chi-let-7e-5p	7815	TGAGGTAGGAGGTTGTATAGTT
chi-miR-30e-5p	7721	TGTAAACATCCTTGACTGGAAGCT
chi-miR-1	7482	TGGAATGTAAAGAAGTATGTAT
chi-miR-148b-3p	7161	TCAGTGCATCACAGAACTTTGT
chi-miR-30c-5p	6951	TGTAAACATCCTACACTCTCAGC
chi-miR-145-3p	6317	ATTCCTGGAAATACTGTTCTT
chi-miR-199b-5p	6238	CCCAGTGTTTAGACTATCTGTTC
chi-miR-199c-5p	6238	CCCAGTGTTTAGACTATCTGTTC
chi-miR-27a-3p	5845	TTCACAGTGGCTAAGTTCCG
chi-miR-450-5p	5743	TTTTGCGATGTGTTCCTAAT
chi-miR-23b-3p	5484	ATCACATTGCCAGGGATTACC
chi-miR-122	5326	TGGAGTGTGACAATGGTGTTTG
chi-miR-23a	5038	ATCACATTGCCAGGGATTTCC
chi-miR-423-3p	4956	AGCTCGGTCTGAGGCCCCTCAGT
chi-miR-320-3p	4888	AAAAGCTGGGTTGAGAGGGCGA
chi-miR-224-5p	4835	CAAGTCACTAGTGGTTCCGTTT
chi-miR-361-3p	4645	TCCCCCAGGTGTGATTCTGATT
chi-miR-186-5p	4634	CAAAGAATTCTCCTTTTGGGCT
chi-miR-103-3p	4592	AGCAGCATTGTACAGGGCTATGA
chi-miR-98-5p	4046	TGAGGTAGTAAGTTGTATTGTT
chi-miR-708-3p	3836	CATCTAGACTGTGAGCTTCTAGA
chi-miR-342-3p	3630	TCTCACACAGAAATCGCACCCA
chi-miR-28-3p	3593	CACTAGATTGAGAGCTCCTGGA
chi-miR-3431-5p	3567	CCTCAGTCAGCCTTGTGGATGT
chi-miR-125b-3p	3425	ACAAGTCAGGCTCTTGGGACC
chi-miR-192-5p	3378	CTGACCTATGAATTGACAGCC
chi-miR-20a-5p	3293	TAAAGTGCTTATAGTGCAGGTAG
chi-miR-30b-5p	3206	TGTAAACATCCTACACTCAGCT
chi-miR-127-3p	3178	TCGGATCCGTCTGAGCTTGG
chi-miR-204-5p	3139	TTCCCTTTGTCATCCTATGCCT
chi-miR-374b-5p	2828	ATATAATACAACCTGCTAAGTG
chi-miR-378-3p	2825	ACTGGACTTGGAGTCAGAAGGC
chi-miR-218	2822	TTGTGCTTGATCTAACCATGT
chi-miR-423-5p	2611	TGAGGGGCAGAGAGCGAGACTTT
chi-miR-424-5p	2595	CAGCAGCAATTCATGTTTTGA
chi-miR-30e-3p	2385	CTTTCAGTCGGATGTTTACAG
chi-miR-200a	2275	TAACACTGTCTGGTAACGATG

chi-miR-660	2187	TACCCATTGCATATCGGAGCTGT
chi-miR-340-5p	2092	TTATAAAGCAATGAGACTGATT
chi-miR-16b-5p	2088	TAGCAGCACGTAAATATTGGGG
chi-miR-7-5p	2011	TGGAAGACTAGTGATTTTGTTGTT
chi-miR-143-5p	1991	GGTGCAGTGCTGCATCTCTGG
chi-miR-151-5p	1938	TCGAGGAGCTCACAGTCTAGT
chi-miR-455-3p	1719	GCAGTCCATGGGCATATACACT
chi-miR-155-5p	1630	TTAATGCTAATCGTGATAGGGGT
chi-miR-200b	1626	TAATACTGCCTGGTAATGATGA
chi-miR-214-3p	1623	TACAGCAGGCACAGACAGGC
chi-miR-93-5p	1582	CAAAGTGCTGTTCGTGCAGGTAG
chi-miR-29b-3p	1577	TAGCACCATTTGAAATCAGT
chi-miR-195-3p	1529	CCAATATTGGCTGTGCTGCTC
chi-miR-328-3p	1503	CTGGCCCTCTCTGCCCTTCCGT
chi-let-7d-5p	1452	AGAGGTAGTAGGTTGCATAGTT
chi-miR-126-5p	1438	CATTATTACTTTTGGTACGCGC
chi-miR-3432-5p	1391	TGCGGGATCTTTAGTTGTGGCG
chi-miR-451-5p	1377	AAACCGTTACCATTACTGA
chi-miR-374a-3p	1325	CTTATCAGGTTGTATTGTAATT
chi-miR-140-5p	1309	CAGTGGTTTTACCCTATGGTAG
chi-miR-148a-5p	1306	AAAGTTCTGAGACACTCCGACT
chi-miR-128-3p	1263	TCACAGTGAACCGGTCTCTTT
chi-miR-374a-5p	1247	TTATAATACAACCTGATAAGT
chi-miR-379-5p	1201	TGGTAGACTATGGAACGTAGG
chi-miR-106b-3p	1172	CCGCACTGTGGGTACTTGCT
chi-miR-1271-5p	1157	CTTGGCACCTAGTAAGTACT
chi-miR-411a-5p	1140	ATAGTAGACCGTATAGCGTAC
chi-miR-30a-3p	1104	CTTTCAGTCGGATGTTTGCAG
chi-miR-106b-5p	1033	TAAAGTGCTGACAGTGCAGAT
chi-miR-107-3p	1029	AGCAGCATTGTACAGGGCTAT
chi-miR-424-3p	988	CAAAACGTGAGGCGCTGCTAT
chi-miR-221-3p	972	AGCTACATTGTCTGCTGGGTTT
chi-miR-1307-3p	894	ACTCGGCGTGGCGTCGGTCGTGG
chi-miR-499-5p	888	TTAAGACTTGCAGTGATGTTT
chi-miR-497-5p	884	AGCAGCACACTGTGGTTTGTAC
chi-miR-28-5p	866	AAGGAGCTCACAGTCTATTGA
chi-miR-17-5p	830	CAAAGTGCTTACAGTGCAGGTAGT
chi-miR-1468-5p	806	CTCCGTTTGCCTGTTTTGCTGA
chi-miR-181b-5p	717	AACATTCATTGCTGTCGGTGGGT
chi-miR-99a-3p	714	CAAGCTCGCTTCTATGGGTCTGT
chi-miR-500-3p	694	ATGCACCTGGGCAAGGATTCT
chi-miR-150	657	TCTCCCAACCCTTGTACCAGTG
chi-miR-19b-3p	656	TGTGCAAATCCATGCAAAACTGA

chi-miR-365-3p	655	TAATGCCCCTAAAAATCCTTAT
chi-miR-146b-5p	646	TGAGAACTGAATTCCATAGGCTGT
chi-miR-9-5p	636	TCTTTGGTTATCTAGCTGTATGA
chi-miR-197-3p	632	TTCACCACCTTCTCCACCCAGC
chi-miR-222-3p	623	AGCTACATCTGGCTACTGGGTCTC
chi-miR-194	608	TGTAACAGCAACTCCATGTGGA
chi-miR-30f-5p	601	TGTAAACACCCTACACTCTCAGC
chi-miR-361-5p	534	TTATCAGAATCTCCAGGGGTAC
chi-miR-193a	527	AACTGGCCCACAAAGTCCC
chi-miR-193b-3p	527	AACTGGCCCACAAAGTCCCGCT
chi-miR-133a-3p	517	TTTGGTCCCCTTCAACCAGCTGT
chi-miR-221-5p	463	ACCTGGCATACAATGTAGATT
chi-miR-331-3p	462	CCCCTGGGCCTATCCTAGAAC
chi-miR-532-3p	447	CCTCCCACACCCAAGGCTTGC
chi-miR-708-5p	436	AAGGAGCTTACAATCTAGCTGGG
chi-miR-483	407	CACTCCTCTCCTCCCGTCTTCT
chi-miR-130a-3p	399	CAGTGCAATGTTAAAAGGGCA
chi-miR-362-5p	382	AATCCTTGGAACCTAGGTGTGAGT
chi-miR-592	357	TTGTGTCAATATGCGATGATGT
chi-miR-195-5p	345	TAGCAGCACAGAAATGTTGG
chi-miR-504	335	AGACCCTGGTCTGCACTCTGT
chi-miR-181d	330	AACATTCATTGTTGTCGGTGGGT
chi-miR-136-3p	319	ATCATCGTCTCAAATGAGTCT
chi-miR-3431-3p	319	ATCTAGAGGACTGACTGAAATT
chi-miR-425-5p	302	AATGACACGATCACTCCCGTTGA
chi-miR-196b	298	TAGGTAGTTTCCTGTTGTTGG
chi-let-7d-3p	257	CTATACGACCTGCTGCCTTTC
chi-miR-10b-3p	257	ACAGATTCGATTCTAGGGGAAT
chi-miR-29c-5p	242	TGACCGATTTCTCCTGGTGTTCA
chi-miR-1343	239	CTCCTGGGGCCCGCACTCTCGC
chi-miR-146a	230	TGAGAACTGAATTCCATAGGTT
chi-miR-34a	226	TGGCAGTGTCTTAGCTGGTTGT
chi-miR-135a	223	TATGGCTTTTTATTCCTATGTGA
chi-miR-1388-5p	223	AGGACTGTCCAACCTGAGAAT
chi-miR-99b-3p	219	CAAGCTCGTGTCTGTGGGTC
chi-miR-335-5p	209	TCAAGAGCAATAACGAAAAAT
chi-miR-136-5p	207	ACTCCATTTGTTTTGATGATGG
chi-miR-409-3p	203	GAATGTTGCTCGGTGAACCCC
chi-let-7b-3p	197	CTATACAACCTACTGCCTTCCT
chi-miR-34c-5p	185	AGGCAGTGTAGTTAGCTGATTGC
chi-miR-874-3p	182	CTGCCCTGGCCCGAGGGACCGAC
chi-miR-214-5p	181	TGCCTGTCTACACTTGCTGTGC
chi-miR-490	178	CAACCTGGAGGACTCCATGCTGT

chi-miR-505-3p	167	TCAACACTTGCTGGTTTCCTCT
chi-miR-1307-5p	166	TCGACCGGACCTCGACC
chi-miR-500-5p	153	TAATCCTTGCTACCTGGGTGAGA
chi-miR-24-5p	150	GTGCCTACTGAGCTGATATC
chi-miR-3958-3p	150	AGATATTGCACGGTTGATCTCT
chi-miR-1388-3p	148	ATCTCAGGTTCGTCAGCCCGCA
chi-let-7a-3p	147	CTATACAATCTACTGTCTTTCC
chi-miR-148b-5p	143	GAAGTTCTGTTATACACTCAGGC
chi-miR-495-3p	140	AAACAAACATGGTGCACTTCTT
chi-miR-493-5p	135	TTGTACATGGTAGGCTTTCATT
chi-miR-22-5p	126	AGTTCTTCAGTGGCAAGCTTT
chi-miR-340-3p	121	TCCGTCTCAGTTACTTTATAGC
chi-miR-296-3p	120	AGGGTTGGGCGGAGGCTTTCCT
chi-miR-382-3p	120	AATCATTCACGGACAACACTT
chi-miR-15b-5p	118	TAGCAGCACATCATGGTTTACA
chi-let-7i-3p	114	CTGCGCAAGCTACTGCCTTGCT
chi-miR-196a	114	TAGGTAGTTTCATGTTGTTGG
chi-miR-335-3p	111	TTTTTCATTATTGCTCCTGACC
chi-miR-3959-5p	111	GGTTGATCAGAGAACATACATT
chi-miR-1249	109	ACGCCCTTCCCCCCCTTCTTCA
chi-miR-129-5p	106	CTTTTTGCGGTCTGGGCTTGC
chi-let-7e-3p	97	CTATACGGCCTCCTAGCTTTCC
chi-miR-127-5p	91	GAAGCTCAGAGGGCTCTGATTC
chi-miR-30f-3p	91	CTGGGAGGAGGCTGTTTACTCT
chi-miR-15a-5p	88	TAGCAGCACATAATGGTTTGTGG
chi-miR-494	84	TGAAACATACACGGGAAACCTCT
chi-miR-331-5p	83	TCTAGGTATGGTCCCAGGGAT
chi-miR-374b-3p	82	CTTATCAGGTTGTATTATCATT
chi-miR-450-3p	82	ATTGGGAACATTTTGCAT
chi-miR-153	79	TTGCATAGTCACAAAAGTGATC
chi-miR-369-3p	79	AATAATACATGGTTGATCTTT
chi-miR-129-3p	74	AAGCCCTTACCCCAAAAAGCAT
chi-miR-200c	73	TAATACTGCCGGGTAATGATGGA
chi-miR-767	71	TGCACCATGGTTGTCTGAGCAT
chi-miR-202-3p	70	AGAGGTGTAGGGCATGGGAA
chi-miR-542-5p	70	TCGGGGATCATCATGTCACGAGA
chi-miR-2483-5p	69	CGTCAACCATCCAGCTGTTTGA
chi-miR-493-3p	69	TGAAGGTCTACTGTGTGCCAGG
chi-miR-181c-5p	68	AACATTCAACCTGTCGGTGAG
chi-miR-181c-3p	67	ACCATCGACCGTTGAGTGGACC
chi-miR-204-3p	67	GCTGGGAAGGCAAAGGGAC
chi-miR-330-3p	66	CAAAGCACACGGCCTGCAGAGA
chi-miR-125a-3p	62	ACAGGTGAGGTTCTTGGGAGC

chi-miR-409-5p	60	AGGTTACCCGAGCAACTTTGCAT
chi-miR-30d-3p	59	TTTCAGTCAGATGTTTGCTGC
chi-miR-432-5p	59	TCTTGGAGTAGGTCATTGGGTGG
chi-miR-1306-5p	58	CCACCTCCCCTGCAAACGTCC
chi-miR-380-3p	58	TATGTAATGTGGTCCACGTCT
chi-miR-429	57	TAATACTGTCTGGTAATGCCG
chi-miR-877-5p	57	GTAGAGGAGATGGCGCAGGGG
chi-miR-191-3p	56	CTGCGCTTGGATTTCGTTCCC
chi-miR-29a-5p	56	ACTGATTTCTTTTGGTGTTCA
chi-miR-378-5p	55	CTCCTGACTCCAGGTCCTGTGT
chi-miR-543-3p	49	AAACATTCGCGGTGCACTTCT
chi-miR-188-5p	48	CATCCCTTGCATGGTGGAGGG
chi-miR-19a	48	TGTGCAAATCTATGCAAAACTGA
chi-miR-411a-3p	48	TATGTAACACGGTCCACTAAC
chi-miR-105a	46	TCAAATGCTCAGACTCCTGTGG
chi-miR-7-3p	46	AACAAATCACAGTCTGCCATA
chi-miR-421-3p	45	ATCAACAGACATTAATTGGGCGC
chi-miR-130b-5p	44	ACTCTTTCCCTGTTGCACTACT
chi-miR-1296	42	TTAGGGCCCTGGCTCCATCTCC
chi-miR-412-5p	42	TGGTCGACCAGTTGGAAAGTAAT
chi-miR-1248-5p	41	ACCTTCTTGTATAAGCACTG
chi-let-7c-3p	40	CTGTACAACCTTCTAGCTTTCC
chi-miR-454-3p	40	TAGTGCAATATTGCTTATAGGGT
chi-miR-487b-3p	39	AATCGTACAGGGTCATCCACTT
chi-miR-345-3p	38	CCCTGAACTAGGGGTCTGGAGG
chi-miR-301a-5p	35	GCTCTGACTTTATTGCACTAC
chi-miR-330-5p	34	TCTCTGGGCCTGTGTCTTAG
chi-miR-329b-3p	33	AACACACCTGGTTAACCTCT
chi-miR-382-5p	32	GAAGTTGTTCGTGGTGGATTCG
chi-miR-411b-5p	32	TGGTCGACCATAAAACGTACGT
chi-miR-27a-5p	31	AGGGCTTAGCTGCTTGTGAGCA
chi-miR-215-5p	30	ATGACCTATGAATTGACAGAC
chi-miR-27b-5p	30	AGAGCTTAGCTGATTGGTGAAC
chi-miR-363-3p	30	AATTGCACGGTATCCATCTGCG
chi-miR-671-3p	30	TCCGGTTCTCAGGGCTCCACC
chi-miR-10a-3p	29	CAAATTCGTATCTAGGGGAAT
chi-miR-18a-5p	29	TAAGGTGCATCTAGTGCAGATAG
chi-miR-362-3p	28	AACACACCTATTCAAGGATTC
chi-miR-130a-5p	27	GCTCTTTTCACATTGTGCTACT
chi-miR-182	27	TTTGGCAATGGTAGAACTCAC
chi-miR-324-5p	27	CGCATCCCCTAGGGCATTGGTGT
chi-miR-376e-3p	27	AACATAGAGGAAAATCCACATT
chi-miR-26b-3p	26	CCTGTTCTCCATTACTTGGCTC

chi-miR-33a-5p	26	GTGCATTGTAGTTGCATTGC
chi-miR-671-5p	26	AGGAAGCCCTGGAGGGGCTGGAGG
chi-miR-135b-5p	25	TATGGCTTTTCATTCCTATGTGA
chi-miR-2411-5p	25	GTGGAGTGACTGTCAGATGCAGCCAGC
chi-miR-2483-3p	25	AAACATCTGGTTGGTTGAGAGA
chi-miR-30c-3p	24	CTGGGAGAGGGTTGTTTACTC
chi-miR-338-3p	24	TCCAGCATCAGTGATTTTGTT
chi-miR-1306-3p	23	ACGTTGGCTCTGGTGGTGATGG
chi-miR-338-5p	23	AACAATATCCTGGTGCTGAGT
chi-miR-381	23	TATACAAGGGCAAGCTCTCTGT
chi-miR-425-3p	23	CATCGGGAATGTCGTGTCCGCC
chi-miR-655	23	ATAATACATGGTTAACCTCTCT
chi-miR-502b-3p	22	ATCCACCTGGGCAAGGATTCTGAA
chi-miR-101-5p	21	TCAGTTATCACAGTGCTGATGC
chi-miR-147-3p	21	GTGTGCGGAAATGCTTCT
chi-miR-206	21	TGGAATGTAAGGAAGTGTGTGGT
chi-miR-491-5p	21	AGTGGGGAACCCTTCCATGAGG
chi-miR-9-3p	21	ATAAAGCTAGATAACCGAAA
chi-miR-144-3p	20	TACAGTATAGATGATGTAC
chi-miR-193b-5p	20	CGGGGTTTTGAGGGCGAGATGA
chi-miR-20b	20	CAAAGTGCTCACAGTGCAGGTAG
chi-miR-379-3p	20	TATGTAACATGGTCCACTAAC
chi-miR-223-3p	19	TGTCAGTTTGTCAAATACCCCA
chi-miR-433	19	ATCATGATGGGCTCCTCGGTGT
chi-miR-758	19	TTTGTGACCTGGTCCACTAACC
chi-miR-93-3p	19	ACTGCTGAGCCAGCACTTCCCGA
chi-let-7g-3p	18	CTGTACAGGCCACTGCCTTGCC
chi-miR-25-5p	18	AGGCGGAGACTTGGGCAATTGCT
chi-miR-376c-3p	18	AACATAGAGGAAATTCCACGT
chi-let-7f-3p	17	CTATACAATCTATTGCCTTCCC
chi-miR-17-3p	17	ACTGCAGTGAAGGCACTTGTAGCA
chi-miR-187	17	TCGTGTCTTGTGTTGCAGCC
chi-miR-190a-5p	17	TGATATGTTTGATATATTAGGTT
chi-miR-34b-3p	17	AATCACTAGTTCCACTGCCATC
chi-miR-154b-5p	16	AGAGGTCTTCCATGGTGCATTC
chi-miR-29b-5p	16	CTGGTTTCACATGGTGGCTTAGA
chi-miR-30b-3p	16	CTGGGAGGTGGATGTTTACTTC
chi-miR-323b	16	CACAATACACGGTCGGCCTCT
chi-miR-326-3p	16	CCTCTGGGCCCTTCCTCCAGC
chi-miR-211	15	TTCCCTTTGTCATCCTTTGCCC
chi-miR-342-5p	15	AGGGGTGCTATCTGTGGTTGAGG
chi-miR-345-5p	15	GCTGACTCCTAGTCCAGTGCT
chi-miR-628-5p	15	ATGCTGACATATTTACTAGAGGG

chi-miR-141	14	TAACACTGTCTGGTAAAGATGG
chi-miR-183	14	TATGGCACTGGTAGAATTCACT
chi-miR-369-5p	14	AGATCGACCGTGTTATATTCG
chi-miR-410-3p	14	AATATAACACAGATGGCCTGT
chi-miR-133a-5p	13	AGCTGGTAAAATGGAACCAAAT
chi-miR-133b	13	TTTGGTCCCCTTCAACCAGCT
chi-miR-146b-3p	13	TGCCCTAGGGACTCAGTTCTGGT
chi-miR-502b-5p	13	TAATTCTTGCTCCCCAGGTGAG
chi-miR-2332	12	CGGTTTAAGGTCTTGGAGACAAAG
chi-miR-3432-3p	12	CAGCAACTAAAGATCCCTCAGG
chi-miR-485-5p	12	AGAGGCTGGCCGTGATGAATTC
chi-miR-224-3p	11	AAATGGTACCCTAGTGACTACA
chi-miR-323a-3p	11	CACATTACACGGTCGACCTCT
chi-miR-582-5p	11	TACAGTTGTTCAACCAGTTACT
chi-miR-20a-3p	10	ACTGCATTATGAGCACTTAAA
chi-miR-217-5p	10	TACTGCATCAGGAACTGATTGGA
chi-miR-301a-3p	10	CAGTGCAATAGTATTGTCAAAGC
chi-miR-383	10	AGATCAGAAGGTGATTGTGGCTT
chi-miR-3955-5p	10	TTTGATGGCTGATCCTCTCACT
chi-miR-130b-3p	9	CAGTGCAATGATGAAAGGGCAT
chi-miR-16a-3p	9	AAATTATCTCCAGTATTAACTGTGCTGC
chi-miR-23b-5p	8	TGGGTTCCTGGCATGCTGATTT
chi-miR-33a-3p	8	CAATGTTTCCACAGTGCATCA
chi-miR-544-5p	8	TCTTGTTAAAAGGCAGATTCT
chi-miR-105b-3p	7	CCACGGATGTTTGAGCATGTGC
chi-miR-1185-3p	7	ATATACAGAGGGAGACTCTTAT
chi-miR-1271-3p	7	AGTGCCTGCTATGTGCCAGG
chi-miR-144-5p	7	TGGGATATCATCATATACTGT
chi-miR-324-3p	7	ACTGCCCCAGGTGCTGCTGGG
chi-miR-106a-5p	6	AAAAGTGCTTACAGTGCAGGTAGC
chi-miR-21-3p	6	CAACAGCAGTCGATGGGCTGT
chi-miR-2404	6	TTGCACTGCATGGTATCTG
chi-miR-26a-3p	6	CCTATTCTCGGTTACTTGCACG
chi-miR-1814	5	GTTTTGTTTGGGTTTGTT
chi-miR-34c-3p	5	AATCACTAACCACACGGCCAGG
chi-miR-487a-3p	5	AATCATACAGGGACATCCAGTT
chi-miR-147-5p	4	TGGAAACACTTCTGCACAAACT
chi-miR-154a-3p	4	AATCATACACGGTTCACCTACT
chi-miR-15b-3p	4	CGAATCATTATTTGCTGCTCT
chi-miR-181b-3p	4	CTCACTGATCAATGAATGCAA
chi-miR-18a-3p	4	ACTGCCCTAAGTGCTCCTTCTGG
chi-miR-223-5p	4	TGTGTATTTGACAAGCTGAGTTG
chi-miR-2411-3p	4	CTGAACTGTCTTACTCCCACATC

chi-miR-376b-3p	4	ATCATAGAGGAAAATCCAT
chi-miR-449a-5p	4	TGGCAGTGTATTGTTAGCTGG
chi-miR-545-3p	4	TCAACAAACATTTATTGTGTGC
chi-miR-665	4	ACCAGTAGGCCGAGGCCCCT
chi-miR-98-3p	4	CTATACAACTTACTACTTTCCC
chi-miR-100-3p	3	CAAGCTTGTGTCTATAGGTAT
chi-miR-16b-3p	3	ACCAATATTATTGTGCTGCTTT
chi-miR-1839	3	AAGGTAGACAGAACAGGTCTTG
chi-miR-216b	3	AAATCTCTGCAGGCAAATGTGAT
chi-miR-2331	3	ACCCTGCAGCCAAAGAAGCT
chi-miR-2432	3	AGGATTCTAGGCTTAGAGGT
chi-miR-326-5p	3	CCTCGTCTGTCTGTTGGGCT
chi-miR-3959-3p	3	TGTATGTCAACTGATCCACAGT
chi-miR-485-3p	3	AGTCATACACGGCTCTCCTCTCT
chi-miR-1185-5p	2	AGAGGATACCCTTTGTATGT
chi-miR-1197-3p	2	TAGGACACATGGTCTACTTCT
chi-miR-128-5p	2	CGGGGCCGTAGCACTGTCTGAGA
chi-miR-134	2	TGTGACTGGTTGACCAGAGGGG
chi-miR-154b-3p	2	ATCATACATGGTTGACCTTTTTT
chi-miR-190a-3p	2	CTATATATCAAACATATTCCT
chi-miR-190b	2	TGATATGTTTGATATTGGGTTG
chi-miR-2318	2	CGTGTATGATGAATTATCTGACC
chi-miR-376a	2	GTAGATTCTCCTTCTATGAGT
chi-miR-376d	2	ATCATAGAGGAAAATCCACAT
chi-miR-454-5p	2	ACCCTATCGATATTGTCTCTG
chi-miR-497-3p	2	TCCAAACCACACTGTGATGTT
chi-miR-877-3p	2	TCCTCTTCTCCCTCCTCCCAGG
chi-miR-92a-5p	2	AGGTTGGGATCAGTTGCAATGCT
chi-miR-96	2	TTTGGCACTAGCACATTTTTG
chi-miR-103-5p	1	AGCTTCTTTACAGTGCTGCCTTGTAGCATT
chi-miR-105b-5p	1	TCAAATGCTCAGACTCCTTGGTG
chi-miR-1224	1	GTGAGGACTCGGGAGGTGGAGG
chi-miR-188-3p	1	CTCCCACATGCAGGGTTTGCA
chi-miR-192-3p	1	CTGCCAATTCCATAGGTCACAGG
chi-miR-19b-5p	1	AGTTTTGCAGGTTTGCATCCAGC
chi-miR-2284e	1	AAAACTTGAATGAACTCTTTGG
chi-miR-29c-3p	1	TAGCACCATTTGAAATTGGTT
chi-miR-301b	1	CAGTGCAATGATATTGTCAAAGC
chi-miR-329a-3p	1	AACACACCTGGTTAACCTTT
chi-miR-33b-3p	1	CAGTGCCTCGGCAGTGCAGCC
chi-miR-346-5p	1	TGTCTGCCCGCATGCCTGCCTCT
chi-miR-376b-5p	1	GGTGGATATTCCTTCTATGTTT
chi-miR-376c-5p	1	GGTGGATATTCCTTCTATGTTT

chi-miR-376e-5p	1	GGTGGATATTCCTTCTATGTTT
chi-miR-380-5p	1	ATGGTTGACCACAGAACATGCGC
chi-miR-411b-3p	1	TATGTCACATGGTCCACTAAT
chi-miR-449b-3p	1	AGCCACAACTGCCCTGCCACTT
chi-miR-499-3p	1	GAACATCACAGCAAGTCTGTGC
chi-miR-502a	1	ATGCACCTGGACAAGGATTC
chi-miR-543-5p	1	ACCTGTGGTGCTTAAGGAG
chi-miR-545-5p	1	CTCAGTAAATGTTTATTGGAT
chi-miR-874-5p	1	CGGCCCCACGCACCAGGGTAAGA

(b) Prolific Jintang black goat

miRNA	Counts	Sequence
chi-miR-99a-5p	802348	AACCCGTAGATCCGATCTTGT
chi-miR-21-5p	788052	TAGCTTATCAGACTGATGTTGAC
chi-miR-148a-3p	739216	TCAGTGCACTACAGAACTTTGT
chi-miR-143-3p	594445	TGAGATGAAGCACTGTAGCTCG
chi-miR-10b-5p	376792	TACCCTGTAGAACCGAATTTGT
chi-miR-26a-5p	253691	TTCAAGTAATCCAGGATAGGCT
chi-miR-27b-3p	119279	TTCACAGTGGCTAAGTTCTGC
chi-miR-125b-5p	113234	TCCCTGAGACCCTAACTTGT
chi-let-7f-5p	106187	TGAGGTAGTAGATTGTATAGTT
chi-miR-101-3p	84764	TACAGTACTGTGATAACTGA
chi-miR-100-5p	84325	AACCCGTAGATCCGAACTTGT
chi-let-7i-5p	84233	TGAGGTAGTAGTTTGTGCTGTT
chi-miR-145-5p	78542	GTCCAGTTTTCCCAGGAATCCCT
chi-miR-126-3p	71065	TCGTACCGTGAGTAATAATGC
chi-miR-199a-3p	65008	ACAGTAGTCTGCACATTGGTT
chi-miR-199b-3p	65003	ACAGTAGTCTGCACATTGGTT
chi-miR-199c-3p	65003	ACAGTAGTCTGCACATTGGTT
chi-let-7g-5p	56145	TGAGGTAGTAGTTTGTACAGTT
chi-miR-199a-5p	46117	CCCAGTGTTCAGACTACCTGTTC
chi-let-7a-5p	39182	TGAGGTAGTAGGTTGTATAGTT
chi-miR-30a-5p	35128	TGTAAACATCCTCGACTGGAAGCT
chi-miR-140-3p	30889	ACCACAGGGTAGAACCACGGAC
chi-miR-125a-5p	29851	TCCCTGAGACCCTTTAACCTGT
chi-let-7c-5p	28806	TGAGGTAGTAGGTTGTATGGTT
chi-let-7b-5p	25720	TGAGGTAGTAGGTTGTGTGGTT
chi-miR-199b-5p	19222	CCCAGTGTTTAGACTATCTGTTC
chi-miR-199c-5p	19222	CCCAGTGTTTAGACTATCTGTTC
chi-miR-29a-3p	17483	TAGCACCATCTGAAATCGGTT
chi-miR-99b-5p	16885	CACCCGTAGAACCGACCTTGCG
chi-miR-25-3p	15636	CATTGCACTTGTCTCGGTCTGA

chi-miR-532-5p	15541	CATGCCTTGAGTGTAGGACCGT
chi-miR-26b-5p	15414	TTCAAGTAATTCAGGATAGGTT
chi-miR-92a-3p	13918	TATTGCACTTGTCCCGGCCTGT
chi-miR-191-5p	13380	CAACGGAATCCCAAAAGCAGCT
chi-miR-10a-5p	13066	TACCCTGTAGATCCGAATTTGT
chi-miR-92b	12273	TATTGCACTTGTCCCGGCCTGT
chi-miR-127-3p	11169	TCGGATCCGTCTGAGCTTGG
chi-miR-151-3p	11044	CTAGACTGAAGCTCCTTGAGG
chi-miR-455-5p	10892	TATGTGCCTTTGGACTACATCG
chi-miR-16a-5p	10852	TAGCAGCACGTAAATATTGGAG
chi-miR-24-3p	10789	TGGCTCAGTTCAGCAGGAAC
chi-miR-1	9954	TGGAATGTAAAGAAGTATGTAT
chi-miR-22-3p	9303	AAGCTGCCAGTTGAAGAAC
chi-miR-202-5p	8722	TTCCTATGCATATACTTCTTT
chi-miR-27a-3p	6901	TTCACAGTGGCTAAGTTCCG
chi-miR-30e-5p	6849	TGTAAACATCCTTGACTGGAAGCT
chi-miR-148b-3p	6767	TCAGTGCATCACAGAACTTTGT
chi-miR-542-3p	6540	TGTGACAGATTGATAACTGA
chi-miR-145-3p	6494	ATTCCTGGAAATACTGTTCTT
chi-miR-122	6443	TGGAGTGTGACAATGGTGTTTG
chi-let-7e-5p	6436	TGAGGTAGGAGGTTGTATAGTT
chi-miR-103-3p	5856	AGCAGCATTGTACAGGGCTATGA
chi-miR-23a	5640	ATCACATTGCCAGGGATTTCC
chi-miR-378-3p	5501	ACTGGACTTGGAGTCAGAAGGC
chi-miR-320-3p	5482	AAAAGCTGGGTTGAGAGGGCGA
chi-miR-23b-3p	5107	ATCACATTGCCAGGGATTACC
chi-miR-30c-5p	5086	TGTAAACATCCTACACTCTCAGC
chi-miR-423-3p	5019	AGCTCGGTCTGAGGCCCCTCAGT
chi-miR-20a-5p	4568	TAAAGTGCTTATAGTGCAGGTAG
chi-miR-224-5p	4459	CAAGTCACTAGTGGTTCCGTTT
chi-miR-28-3p	4436	CACTAGATTGAGAGCTCCTGGA
chi-miR-379-5p	4338	TGGTAGACTATGGAACGTAGG
chi-miR-218	4309	TTGTGCTTGATCTAACCATGT
chi-miR-361-3p	4222	TCCCCCAGGTGTGATTCTGATT
chi-miR-3431-5p	4004	CCTCAGTCAGCCTTGTGGATGT
chi-miR-186-5p	3988	CAAAGAATTCTCCTTTTGGGCT
chi-miR-7-5p	3834	TGGAAGACTAGTGATTTTGTTGTT
chi-miR-125b-3p	3694	ACAAGTCAGGCTCTTGGGACC
chi-miR-708-3p	3632	CATCTAGACTGTGAGCTTCTAGA
chi-miR-342-3p	3574	TCTCACACAGAAATCGCACCCA
chi-miR-192-5p	3404	CTGACCTATGAATTGACAGCC
chi-miR-450-5p	3396	TTTTGCGATGTGTTCCTAAT
chi-miR-98-5p	3272	TGAGGTAGTAAGTTGTATTGTT

chi-miR-214-3p	3206	TACAGCAGGCACAGACAGGC
chi-miR-411a-5p	3020	ATAGTAGACCGTATAGCGTAC
chi-miR-16b-5p	2979	TAGCAGCACGTAAATATTGGGG
chi-miR-423-5p	2804	TGAGGGGCAGAGAGCGAGACTTT
chi-miR-660	2465	TACCCATTGCATATCGGAGCTGT
chi-miR-30b-5p	2431	TGTAAACATCCTACACTCAGCT
chi-miR-374b-5p	2345	ATATAATACAACCTGCTAAGTG
chi-miR-340-5p	2248	TTATAAAGCAATGAGACTGATT
chi-miR-455-3p	2098	GCAGTCCATGGGCATATACACT
chi-miR-424-5p	2062	CAGCAGCAATTCATGTTTTGA
chi-miR-143-5p	1966	GGTGCAGTGCTGCATCTCTGG
chi-miR-126-5p	1950	CATTATTACTTTTGGTACGCGC
chi-miR-155-5p	1794	TTAATGCTAATCGTGATAGGGGT
chi-miR-195-3p	1758	CCAATATTGGCTGTGCTGCTC
chi-miR-93-5p	1690	CAAAGTGCTGTTCGTGCAGGTAG
chi-miR-30e-3p	1676	CTTTCAGTCGGATGTTTACAG
chi-miR-151-5p	1636	TCGAGGAGCTCACAGTCTAGT
chi-miR-150	1467	TCTCCCAACCCTTGTACCAGTG
chi-miR-451-5p	1427	AAACCGTTACCATTACTGA
chi-miR-204-5p	1397	TTCCCTTTGTCATCCTATGCCT
chi-miR-3432-5p	1382	TGCGGGATCTTTAGTTGTGGCG
chi-let-7d-5p	1291	AGAGGTAGTAGGTTGCATAGTT
chi-miR-106b-3p	1273	CCGCACTGTGGGTACTTGCT
chi-miR-140-5p	1265	CAGTGGTTTTACCCTATGGTAG
chi-miR-17-5p	1252	CAAAGTGCTTACAGTGCAGGTAGT
chi-miR-148a-5p	1204	AAAGTTCTGAGACACTCCGACT
chi-miR-1468-5p	1190	CTCCGTTTGCCTGTTTTGCTGA
chi-miR-29b-3p	1174	TAGCACCATTTGAAATCAGT
chi-miR-128-3p	1166	TCACAGTGAACCGGTCTCTTT
chi-miR-328-3p	1147	CTGGCCCTCTCTGCCCTTCCGT
chi-miR-374a-3p	1132	CTTATCAGGTTGTATTGTAATT
chi-miR-28-5p	1128	AAGGAGCTCACAGTCTATTGA
chi-miR-1271-5p	1114	CTTGGCACCTAGTAAGTACT
chi-miR-181b-5p	1110	AACATTCATTGCTGTCGGTGGGT
chi-miR-30a-3p	1100	CTTTCAGTCGGATGTTTGCAG
chi-miR-107-3p	1027	AGCAGCATTGTACAGGGCTAT
chi-miR-106b-5p	1010	TAAAGTGCTGACAGTGCAGAT
chi-miR-146b-5p	953	TGAGAACTGAATTCCATAGGCTGT
chi-miR-221-3p	931	AGCTACATTGTCTGCTGGGTTT
chi-miR-374a-5p	917	TTATAATACAACCTGATAAGT
chi-miR-136-3p	877	ATCATCGTCTCAAATGAGTCT
chi-miR-409-3p	873	GAATGTTGCTCGGTGAACCCC
chi-miR-497-5p	829	AGCAGCACACTGTGGTTTGTAC

chi-miR-1307-3p	806	ACTCGGCGTGGCGTCGGTCGTGG
chi-miR-19b-3p	752	TGTGCAAATCCATGCAAAACTGA
chi-miR-493-5p	741	TTGTACATGGTAGGCTTTCATT
chi-miR-500-3p	690	ATGCACCTGGGCAAGGATTCT
chi-miR-99a-3p	673	CAAGCTCGCTTCTATGGGTCTGT
chi-miR-222-3p	647	AGCTACATCTGGCTACTGGGTCTC
chi-miR-133a-3p	626	TTTGGTCCCCTTCAACCAGCTGT
chi-miR-30f-5p	624	TGTAAACACCCTACACTCTCAGC
chi-miR-194	584	TGTAACAGCAACTCCATGTGGA
chi-miR-3958-3p	576	AGATATTGCACGGTTGATCTCT
chi-miR-136-5p	574	ACTCCATTTGTTTTGATGATGG
chi-miR-197-3p	572	TTCACCACCTTCTCCACCCAGC
chi-miR-499-5p	539	TTAAGACTTGCAGTGATGTTT
chi-miR-361-5p	506	TTATCAGAATCTCCAGGGGTAC
chi-miR-221-5p	503	ACCTGGCATACAATGTAGATT
chi-miR-532-3p	497	CCTCCCACACCCAAGGCTTGC
chi-miR-331-3p	483	CCCCTGGGCCTATCCTAGAAC
chi-miR-424-3p	462	CAAAACGTGAGGCGCTGCTAT
chi-miR-708-5p	454	AAGGAGCTTACAATCTAGCTGGG
chi-miR-365-3p	452	TAATGCCCCTAAAAATCCTTAT
chi-miR-382-3p	451	AATCATTCACGGACAACACTT
chi-miR-130a-3p	450	CAGTGCAATGTTAAAAGGGCA
chi-miR-494	440	TGAAACATACACGGGAAACCTCT
chi-miR-493-3p	413	TGAAGGTCTACTGTGTGCCAGG
chi-miR-362-5p	401	AATCCTTGGAACCTAGGTGTGAGT
chi-miR-3959-5p	384	GGTTGATCAGAGAACATACATT
chi-miR-146a	378	TGAGAACTGAATTCCATAGGTT
chi-miR-9-5p	369	TCTTTGGTTATCTAGCTGTATGA
chi-miR-195-5p	365	TAGCAGCACAGAAATGTTGG
chi-miR-3431-3p	353	ATCTAGAGGACTGACTGAAATT
chi-miR-495-3p	339	AAACAAACATGGTGCACTTCTT
chi-miR-193a	312	AACTGGCCCACAAAGTCCC
chi-miR-193b-3p	312	AACTGGCCCACAAAGTCCCGCT
chi-miR-200a	303	TAACACTGTCTGGTAACGATG
chi-miR-127-5p	298	GAAGCTCAGAGGGCTCTGATTC
chi-miR-34a	297	TGGCAGTGTCTTAGCTGGTTGT
chi-miR-214-5p	284	TGCCTGTCTACACTTGCTGTGC
chi-miR-99b-3p	258	CAAGCTCGTGTCTGTGGGTC
chi-miR-425-5p	256	AATGACACGATCACTCCCGTTGA
chi-miR-381	251	TATACAAGGGCAAGCTCTCTGT
chi-miR-409-5p	243	AGGTTACCCGAGCAACTTTGCAT
chi-miR-369-3p	235	AATAATACATGGTTGATCTTT
chi-miR-15b-5p	225	TAGCAGCACATCATGGTTTACA

chi-miR-200b	225	TAATACTGCCTGGTAATGATGA
chi-miR-335-5p	220	TCAAGAGCAATAACGAAAAAT
chi-miR-432-5p	220	TCTTGGAGTAGGTCATTGGGTGG
chi-miR-10b-3p	211	ACAGATTCGATTCTAGGGGAAT
chi-miR-874-3p	203	CTGCCCTGGCCCGAGGGACCGAC
chi-miR-335-3p	198	TTTTTCATTATTGCTCCTGACC
chi-miR-380-3p	197	TATGTAATGTGGTCCACGTCT
chi-let-7b-3p	192	CTATACAACCTACTGCCTTCCT
chi-let-7d-3p	189	CTATACGACCTGCTGCCTTTC
chi-miR-1388-5p	186	AGGACTGTCCAACCTGAGAAT
chi-miR-1343	180	CTCCTGGGGCCCGCACTCTCGC
chi-miR-500-5p	180	TAATCCTTGCTACCTGGGTGAGA
chi-miR-411a-3p	178	TATGTAACACGGTCCACTAAC
chi-miR-129-5p	174	CTTTTTGCGGTCTGGGCTTGC
chi-miR-382-5p	163	GAAGTTGTTCGTGGTGGATTCG
chi-miR-130b-5p	162	ACTCTTTCCCTGTTGCACTACT
chi-miR-29c-5p	162	TGACCGATTTCTCCTGGTGTTCA
chi-miR-490	147	CAACCTGGAGGACTCCATGCTGT
chi-miR-412-5p	144	TGGTCGACCAGTTGGAAAGTAAT
chi-miR-504	143	AGACCCTGGTCTGCACTCTGT
chi-miR-592	142	TTGTGTCAATATGCGATGATGT
chi-miR-505-3p	140	TCAACACTTGCTGGTTTCCTCT
chi-let-7a-3p	136	CTATACAATCTACTGTCTTTCC
chi-miR-15a-5p	132	TAGCAGCACATAATGGTTTGTGG
chi-miR-1307-5p	131	TCGACCGGACCTCGACC
chi-miR-1388-3p	128	ATCTCAGGTTCGTCAGCCCGCA
chi-miR-148b-5p	126	GAAGTTCTGTTATACACTCAGGC
chi-miR-2483-5p	125	CGTCAACCATCCAGCTGTTTGA
chi-miR-487b-3p	124	AATCGTACAGGGTCATCCACTT
chi-miR-24-5p	123	GTGCCTACTGAGCTGATATC
chi-miR-30f-3p	116	CTGGGAGGAGGCTGTTTACTCT
chi-miR-340-3p	116	TCCGTCTCAGTTACTTTATAGC
chi-miR-296-3p	111	AGGGTTGGGCGGAGGCTTTCCT
chi-miR-543-3p	111	AAACATTCGCGGTGCACTTCT
chi-miR-181d	105	AACATTCATTGTTGTCGGTGGGT
chi-let-7i-3p	100	CTGCGCAAGCTACTGCCTTGCT
chi-miR-22-5p	98	AGTTCTTCAGTGGCAAGCTTT
chi-miR-655	94	ATAATACATGGTTAACCTCTCT
chi-miR-331-5p	91	TCTAGGTATGGTCCCAGGGAT
chi-miR-330-3p	88	CAAAGCACACGGCCTGCAGAGA
chi-miR-27a-5p	84	AGGGCTTAGCTGCTTGTGAGCA
chi-miR-433	83	ATCATGATGGGCTCCTCGGTGT
chi-let-7e-3p	82	CTATACGGCCTCCTAGCTTTCC

chi-miR-129-3p	82	AAGCCCTTACCCCAAAAAGCAT
chi-miR-34c-5p	78	AGGCAGTGTAGTTAGCTGATTGC
chi-miR-411b-5p	78	TGGTCGACCATAAAACGTACGT
chi-miR-135a	75	TATGGCTTTTTATTCCTATGTGA
chi-miR-223-3p	75	TGTCAGTTTGTCAAATACCCCA
chi-miR-1306-5p	70	CCACCTCCCCTGCAAACGTCC
chi-miR-378-5p	70	CTCCTGACTCCAGGTCCTGTGT
chi-miR-202-3p	69	AGAGGTGTAGGGCATGGGAA
chi-miR-329b-3p	67	AACACACCTGGTTAACCTCT
chi-miR-483	66	CACTCCTCTCCTCCCGTCTTCT
chi-miR-215-5p	64	ATGACCTATGAATTGACAGAC
chi-miR-379-3p	64	TATGTAACATGGTCCACTAAC
chi-miR-19a	61	TGTGCAAATCTATGCAAAACTGA
chi-miR-877-5p	61	GTAGAGGAGATGGCGCAGGGG
chi-miR-376e-3p	60	AACATAGAGGAAAATCCACATT
chi-miR-758	60	TTTGTGACCTGGTCCACTAACC
chi-miR-27b-5p	59	AGAGCTTAGCTGATTGGTGAAC
chi-miR-369-5p	59	AGATCGACCGTGTTATATTCG
chi-miR-125a-3p	58	ACAGGTGAGGTTCTTGGGAGC
chi-miR-497-3p	56	TCCAAACCACACTGTGATGTT
chi-miR-154b-5p	55	AGAGGTCTTCCATGGTGCATTC
chi-miR-376c-3p	53	AACATAGAGGAAATTCCACGT
chi-miR-7-3p	52	AACAAATCACAGTCTGCCATA
chi-miR-21-3p	50	CAACAGCAGTCGATGGGCTGT
chi-miR-30d-3p	49	TTTCAGTCAGATGTTTGCTGC
chi-miR-3955-5p	49	TTTGATGGCTGATCCTCTCACT
chi-miR-421-3p	48	ATCAACAGACATTAATTGGGCGC
chi-miR-191-3p	47	CTGCGCTTGGATTTCGTTCCC
chi-miR-29a-5p	46	ACTGATTTCTTTTGGTGTTCA
chi-miR-410-3p	46	AATATAACACAGATGGCCTGT
chi-miR-454-3p	46	TAGTGCAATATTGCTTATAGGGT
chi-miR-671-5p	46	AGGAAGCCCTGGAGGGGCTGGAGG
chi-miR-188-5p	45	CATCCCTTGCATGGTGGAGGG
chi-miR-374b-3p	44	CTTATCAGGTTGTATTATCATT
chi-miR-211	43	TTCCCTTTGTCATCCTTTGCCC
chi-miR-301a-5p	43	GCTCTGACTTTATTGCACTAC
chi-miR-330-5p	42	TCTCTGGGCCTGTGTCTTAG
chi-miR-485-5p	39	AGAGGCTGGCCGTGATGAATTC
chi-miR-542-5p	39	TCGGGGATCATCATGTCACGAGA
chi-miR-1249	38	ACGCCCTTCCCCCCCTTCTTCA
chi-miR-323b	38	CACAATACACGGTCGGCCTCT
chi-miR-345-3p	38	CCCTGAACTAGGGGTCTGGAGG
chi-miR-383	37	AGATCAGAAGGTGATTGTGGCTT

chi-let-7c-3p	36	CTGTACAACCTTCTAGCTTTCC
chi-miR-133b	36	TTTGGTCCCCTTCAACCAGCT
chi-miR-18a-5p	36	TAAGGTGCATCTAGTGCAGATAG
chi-miR-2483-3p	36	AAACATCTGGTTGGTTGAGAGA
chi-miR-450-3p	36	ATTGGGAACATTTTGCAT
chi-miR-200c	33	TAATACTGCCGGGTAATGATGGA
chi-miR-362-3p	33	AACACACCTATTCAAGGATTC
chi-miR-1296	32	TTAGGGCCCTGGCTCCATCTCC
chi-miR-2318	32	CGTGTATGATGAATTATCTGACC
chi-miR-20b	30	CAAAGTGCTCACAGTGCAGGTAG
chi-miR-181c-3p	29	ACCATCGACCGTTGAGTGGACC
chi-miR-326-3p	29	CCTCTGGGCCCTTCCTCCAGC
chi-miR-582-5p	28	TACAGTTGTTCAACCAGTTACT
chi-miR-130a-5p	27	GCTCTTTTCACATTGTGCTACT
chi-miR-206	27	TGGAATGTAAGGAAGTGTGTGGT
chi-miR-33a-5p	27	GTGCATTGTAGTTGCATTGC
chi-miR-485-3p	26	AGTCATACACGGCTCTCCTCTCT
chi-miR-671-3p	26	TCCGGTTCTCAGGGCTCCACC
chi-miR-153	25	TTGCATAGTCACAAAAGTGATC
chi-miR-181c-5p	25	AACATTCAACCTGTCGGTGAG
chi-miR-665	25	ACCAGTAGGCCGAGGCCCCT
chi-miR-30c-3p	24	CTGGGAGAGGGTTGTTTACTC
chi-miR-363-3p	24	AATTGCACGGTATCCATCTGCG
chi-miR-425-3p	24	CATCGGGAATGTCGTGTCCGCC
chi-miR-144-3p	23	TACAGTATAGATGATGTAC
chi-miR-1185-3p	22	ATATACAGAGGGAGACTCTTAT
chi-miR-1197-3p	22	TAGGACACATGGTCTACTTCT
chi-miR-133a-5p	22	AGCTGGTAAAATGGAACCAAAT
chi-miR-134	22	TGTGACTGGTTGACCAGAGGGG
chi-miR-204-3p	22	GCTGGGAAGGCAAAGGGAC
chi-miR-2411-5p	22	GTGGAGTGACTGTCAGATGCAGCCAGC
chi-miR-323a-3p	22	CACATTACACGGTCGACCTCT
chi-miR-147-3p	21	GTGTGCGGAAATGCTTCT
chi-miR-25-5p	21	AGGCGGAGACTTGGGCAATTGCT
chi-miR-628-5p	21	ATGCTGACATATTTACTAGAGGG
chi-miR-17-3p	20	ACTGCAGTGAAGGCACTTGTAGCA
chi-miR-187	20	TCGTGTCTTGTGTTGCAGCC
chi-miR-223-5p	20	TGTGTATTTGACAAGCTGAGTTG
chi-miR-502b-3p	20	ATCCACCTGGGCAAGGATTCTGAA
chi-miR-101-5p	19	TCAGTTATCACAGTGCTGATGC
chi-miR-26b-3p	19	CCTGTTCTCCATTACTTGGCTC
chi-miR-29b-5p	19	CTGGTTTCACATGGTGGCTTAGA
chi-miR-1248-5p	18	ACCTTCTTGTATAAGCACTG

chi-miR-1306-3p	17	ACGTTGGCTCTGGTGGTGATGG
chi-miR-182	17	TTTGGCAATGGTAGAACTCAC
chi-miR-196a	17	TAGGTAGTTTCATGTTGTTGG
chi-miR-324-5p	17	CGCATCCCCTAGGGCATTGGTGT
chi-miR-345-5p	17	GCTGACTCCTAGTCCAGTGCT
chi-miR-190a-5p	16	TGATATGTTTGATATATTAGGTT
chi-miR-30b-3p	16	CTGGGAGGTGGATGTTTACTTC
chi-miR-376b-3p	16	ATCATAGAGGAAAATCCAT
chi-miR-491-5p	16	AGTGGGGAACCCTTCCATGAGG
chi-miR-196b	15	TAGGTAGTTTCCTGTTGTTGG
chi-miR-154a-3p	14	AATCATACACGGTTCACCTACT
chi-miR-16a-3p	14	AAATTATCTCCAGTATTAACTGTGCTGC
chi-miR-338-3p	13	TCCAGCATCAGTGATTTTGTT
chi-let-7f-3p	12	CTATACAATCTATTGCCTTCCC
chi-miR-130b-3p	12	CAGTGCAATGATGAAAGGGCAT
chi-miR-193b-5p	12	CGGGGTTTTGAGGGCGAGATGA
chi-miR-301a-3p	12	CAGTGCAATAGTATTGTCAAAGC
chi-let-7g-3p	11	CTGTACAGGCCACTGCCTTGCC
chi-miR-10a-3p	11	CAAATTCGTATCTAGGGGAAT
chi-miR-144-5p	11	TGGGATATCATCATATACTGT
chi-miR-15b-3p	11	CGAATCATTATTTGCTGCTCT
chi-miR-1814	11	GTTTTGTTTGGGTTTGTT
chi-miR-2331	11	ACCCTGCAGCCAAAGAAGCT
chi-miR-487a-3p	11	AATCATACAGGGACATCCAGTT
chi-miR-544-5p	11	TCTTGTTAAAAGGCAGATTCT
chi-miR-93-3p	11	ACTGCTGAGCCAGCACTTCCCGA
chi-miR-146b-3p	10	TGCCCTAGGGACTCAGTTCTGGT
chi-miR-26a-3p	10	CCTATTCTCGGTTACTTGCACG
chi-miR-33a-3p	10	CAATGTTTCCACAGTGCATCA
chi-miR-342-5p	10	AGGGGTGCTATCTGTGGTTGAGG
chi-miR-3959-3p	10	TGTATGTCAACTGATCCACAGT
chi-miR-1271-3p	9	AGTGCCTGCTATGTGCCAGG
chi-miR-183	9	TATGGCACTGGTAGAATTCACT
chi-miR-20a-3p	9	ACTGCATTATGAGCACTTAAA
chi-miR-376d	9	ATCATAGAGGAAAATCCACAT
chi-miR-502b-5p	9	TAATTCTTGCTCCCCAGGTGAG
chi-miR-105a	8	TCAAATGCTCAGACTCCTGTGG
chi-miR-106a-5p	8	AAAAGTGCTTACAGTGCAGGTAGC
chi-miR-18a-3p	8	ACTGCCCTAAGTGCTCCTTCTGG
chi-miR-2332	8	CGGTTTAAGGTCTTGGAGACAAAG
chi-miR-324-3p	8	ACTGCCCCAGGTGCTGCTGGG
chi-miR-338-5p	8	AACAATATCCTGGTGCTGAGT
chi-miR-34b-3p	8	AATCACTAGTTCCACTGCCATC

chi-miR-767	8	TGCACCATGGTTGTCTGAGCAT
chi-miR-16b-3p	7	ACCAATATTATTGTGCTGCTTT
chi-miR-3432-3p	7	CAGCAACTAAAGATCCCTCAGG
chi-miR-380-5p	7	ATGGTTGACCACAGAACATGCGC
chi-miR-429	7	TAATACTGTCTGGTAATGCCG
chi-miR-135b-5p	6	TATGGCTTTTCATTCCTATGTGA
chi-miR-192-3p	6	CTGCCAATTCCATAGGTCACAGG
chi-miR-217-5p	6	TACTGCATCAGGAACTGATTGGA
chi-miR-329a-3p	6	AACACACCTGGTTAACCTTT
chi-miR-376a	6	GTAGATTCTCCTTCTATGAGT
chi-miR-92a-5p	6	AGGTTGGGATCAGTTGCAATGCT
chi-miR-656	5	AATATTATACAGTCAACCTCT
chi-miR-9-3p	5	ATAAAGCTAGATAACCGAAA
chi-miR-98-3p	5	CTATACAACTTACTACTTTCCC
chi-miR-1185-5p	4	AGAGGATACCCTTTGTATGT
chi-miR-141	4	TAACACTGTCTGGTAAAGATGG
chi-miR-190b	4	TGATATGTTTGATATTGGGTTG
chi-miR-376b-5p	4	GGTGGATATTCCTTCTATGTTT
chi-miR-376c-5p	4	GGTGGATATTCCTTCTATGTTT
chi-miR-376e-5p	4	GGTGGATATTCCTTCTATGTTT
chi-miR-100-3p	3	CAAGCTTGTGTCTATAGGTAT
chi-miR-128-5p	3	CGGGGCCGTAGCACTGTCTGAGA
chi-miR-147-5p	3	TGGAAACACTTCTGCACAAACT
chi-miR-181b-3p	3	CTCACTGATCAATGAATGCAA
chi-miR-184	3	TGGACGGAGAACTGATAAGGGT
chi-miR-186-3p	3	CCCAAAGGTGAATTTTTTGG
chi-miR-2335	3	GATAATGATGACTAACTGAAT
chi-miR-2404	3	TTGCACTGCATGGTATCTG
chi-miR-412-3p	3	ACTTCACCTGGTCCACTAGCT
chi-miR-449a-5p	3	TGGCAGTGTATTGTTAGCTGG
chi-miR-545-3p	3	TCAACAAACATTTATTGTGTGC
chi-miR-103-5p	2	AGCTTCTTTACAGTGCTGCCTTGTAGCATT
chi-miR-105b-3p	2	CCACGGATGTTTGAGCATGTGC
chi-miR-19b-5p	2	AGTTTTGCAGGTTTGCATCCAGC
chi-miR-216b	2	AAATCTCTGCAGGCAAATGTGAT
chi-miR-224-3p	2	AAATGGTACCCTAGTGACTACA
chi-miR-2432	2	AGGATTCTAGGCTTAGAGGT
chi-miR-33b-5p	2	GTGCATTGCTGTTGCATTG
chi-miR-346-5p	2	TGTCTGCCCGCATGCCTGCCTCT
chi-miR-411b-3p	2	TATGTCACATGGTCCACTAAT
chi-miR-454-5p	2	ACCCTATCGATATTGTCTCTG
chi-miR-499-3p	2	GAACATCACAGCAAGTCTGTGC
chi-miR-502a	2	ATGCACCTGGACAAGGATTC

chi-miR-874-5p	2	CGGCCCCACGCACCAGGGTAAGA
chi-miR-96	2	TTTGGCACTAGCACATTTTTG
chi-miR-105b-5p	1	TCAAATGCTCAGACTCCTTGGTG
chi-miR-1224	1	GTGAGGACTCGGGAGGTGGAGG
chi-miR-154b-3p	1	ATCATACATGGTTGACCTTTTTT
chi-miR-1839	1	AAGGTAGACAGAACAGGTCTTG
chi-miR-18b-5p	1	TAAGGTGCATCTAGTGCAG
chi-miR-190a-3p	1	CTATATATCAAACATATTCCT
chi-miR-197-5p	1	TCTGTGCTCCGGGGGCTGTGC
chi-miR-23b-5p	1	TGGGTTCCTGGCATGCTGATTT
chi-miR-326-5p	1	CCTCGTCTGTCTGTTGGGCT
chi-miR-34c-3p	1	AATCACTAACCACACGGCCAGG
chi-miR-377	1	AATCACACAAAGGCAACTTTTG
chi-miR-3955-3p	1	TGGGATTTTAGTCCATCATAGAG
chi-miR-545-5p	1	CTCAGTAAATGTTTATTGGAT

Supplementary Table S3

Novel miRNAs in the ovaries of Tibetan goat (**a**) and Jintang black goat (**b**) during follicular phase

(a) Non-prolific Tbetan goats

miR_Name	Counts	Sequence
novel-miR-8	6803	AACATTCAACGCTGTCGGTGAGT
novel-miR-93	2701	AACTGTTTGCAGAGGAAACTGA
novel-miR-20	2827	TCCCTGAGACCCTAACTTGTGAT
novel-miR-94	2352	TGAGGTAGTAGATTGTATAGTTT
novel-miR-77	2160	TACAGTACTGTGATAACTGAAGA
novel-miR-72	1162	AGGCAAGATGCTGGCATAGCTGT
novel-miR-81	829	TAGCAGCGGGAACAGTACTGCAG
novel-miR-78	532	TATTGCACATTACTAAGTTGCA
novel-miR-70	412	TGAGATGAAGCACTGTAGCTC
novel-miR-37	161	GCCTGCTGGGGTGGAACCTGGT
novel-miR-83	242	AGAGGTAAAAAATTGATTTGACT
novel-miR-91	249	TGATTGGTACTTCTTAGAGTGA
novel-miR-27	196	TGAGACCTCCGGGTTCTGAGCT
novel-miR-68	163	GTGCCTACTGAGCTGAAACACA
novel-miR-38	62	TATGTGGGACGGTAAACCGCTT
novel-miR-36	158	GAGAGATCAGAGGCGCAGAGT
novel-miR-12	137	TGTTGTACTTTTTTTTTTGTTC
novel-miR-52	159	TGTAACAGCAACTCCATGTGGAA
novel-miR-71	108	GTCCAGTTTTCCCAGGAATCCCT
novel-miR-92	105	ATTGGCACGTCTTGGAATGCA
novel-miR-66	65	TCTGGTGCTTAGACTCTGTGCT
novel-miR-76	70	TGAGGTAGTAGATTGTATAGTT
novel-miR-56	41	AACTTTTGCCCCTAGTAACGGACT
novel-miR-75	44	TGAGGTAGTAGGTTGTATAGTTTT
novel-miR-62	56	ATGTCCGCGGGTTCCCTATCCC
novel-miR-14	57	TTCTCTGTTCATGTGGACGTGT
novel-miR-64	43	TGAGGTAGTAGTTTGTGCTGTT
novel-miR-55	57	GTACAGTACTGTGATAACTGA
novel-miR-9	47	GTTTCCGGTCGCTGTGCTCTCG
novel-miR-45	45	TGAGGTAGTAGTTTGTACAGTT
novel-miR-43	28	TCACAGTGAACCGGTCTCTTTC
novel-miR-21	32	TGAGGTAGTAGGTTGTATAGTT
novel-miR-13	29	GAAAGGTTCATTTGGGTTTTT
novel-miR-15	20	TTGCAGTGATGACTTGAGTCTG
novel-miR-31	23	ATCATGTATGATACTGCAAACA

novel-miR-41	17	TTGGACACTTCAGTACTGCTACA
novel-miR-67	12	TGCCCCGAGGCTTGTGAAGCTC
novel-miR-24	18	AAAACTCTGAATGAACTTTATG
novel-miR-35	25	TGAGGTAGTAGGTTGTATGGTT
novel-miR-48	11	TGGAAGGCCTGGCTTTGCAGCGT
novel-miR-65	21	TGAGGTAGTAGGTTGTGTGGTT
novel-miR-46	9	CTTAGAGCTTCCCCGCCTTGGGGA
novel-miR-69	22	CTCTGCCCGCTCTCTGTCTTACA
novel-miR-53	7	AAAACCTGAATGAACTTTTGAG
novel-miR-51	15	AAAACCTGAGTGAACTTTCTAG
novel-miR-39	14	CATCTGGTTGGTATGTTTTGCT
novel-miR-33	15	GCGGCCCGCGGGCTCGGATGCT
novel-miR-40	11	AAACCGACATGAACTTTTTGG
novel-miR-79	10	TGTTTGACATGAGAGCTTCCAGA
novel-miR-74	8	TGGCTCAGTTCAGCAGGAAC
novel-miR-11	21	GAAAACCTGAATGAACTTTTG
novel-miR-80	10	TTTAAGTACACGATGAACTGC
novel-miR-50	8	AAACCCGAATGAACTTTTTGA
novel-miR-6	10	AAAAACCTGAATGAACCTTTTG
novel-miR-61	8	CCTCATGTGGAATCTTTAGTTG
novel-miR-49	10	TAGAGAAGCACTGGGGGAAAGT
novel-miR-89	8	TGATTGGCATTTCTTAGAGTGA
novel-miR-4	6	TCTCAGGGTCTGTGAGCTCCTCG
novel-miR-16	6	TGGAATGTAAAGAAGTATGTAT
novel-miR-17	6	GCCCTGTCCTCCAGGAGCTC
novel-miR-7	7	AGGCCAAAGCAAACGACTGTGC
novel-miR-84	134	AATCGGTATCTGTCTGAGTAGA
novel-miR-90	57	AGTAGTGCCTCTCTGAGTGT
novel-miR-58	56	CCTATTCTTGATTACTTGTTT
novel-miR-63	56	TAGGTAGTTTCATGTTGTTGGGA
novel-miR-32	25	ATCTGAATCAGTCTATGTTGACT
novel-miR-57	24	TCCGGGGAAAGCAGGAGTGAG
novel-miR-87	21	TGAAAGGACCTGCCTGGGTAGA
novel-miR-3	21	CCCAGGGATGTAGCTCCTAGT
novel-miR-18	17	TGTAAACATCCTCGACTGGAAGCT
novel-miR-88	15	TTCACAAGAAGGTGTCATTCA
novel-miR-30	13	GAAAACCTGAACAAACTTTGTG
novel-miR-34	13	TTCTCATTGGCCTCACGTCCTGT
novel-miR-28	12	TAGATCTTTACCTATTAGCA
novel-miR-54	12	ATTGTCCTTGCTGTTTGGAGAT
novel-miR-2	11	ACCGGGGACTGTGGAGACGGA
novel-miR-42	11	TCACTGGGCATCCTCTGCTTTA
novel-miR-29	12	TTAATTTTTGCAAGGCTTTTC

novel-miR-82	9	TACGGGGATAGGTCTCAAGA
novel-miR-47	9	ACCCTGTCCTCCAGGAGCTC
novel-miR-10	9	TTGAGGCTCCGAGCTTTGGTGT
novel-miR-25	9	TTTCTTTGAGCTTCTTTCTTTC
novel-miR-73	9	TGAGTGTGTGTGTGGGAGTGT
novel-miR-85	8	TACTGTGCCACGGATGGGTAGC
novel-miR-5	8	ACTGGGCAATCCTTTCACTCT
novel-miR-19	7	TCAAGGTCCGCTGTGAACACGG
novel-miR-59	7	CTCACTGATGAGCAGCTTCTGAC
novel-miR-26	6	GATGAGGCTCAGCGAGCCCTGGT
novel-miR-23	6	CAGCACTGTCCGGTAAGATGT
novel-miR-22	6	ATCGTTACCAGACAGTGTTAGA
novel-miR-1	6	ACATCTCTGGGGAATTGCTGAGT
novel-miR-60	6	TGTAGGGATGGAAGCCATGAA
novel-miR-86	5	TGCCTAGAGAGGTTCTGTTTACT
novel-miR-44	5	CCAGGAGTGTCTTTGGATTGTC

(b) Prolific Jintang black goats

**miR_Name**	Counts	Sequence
novel-miR-8	9244	AACATTCAACGCTGTCGGTGAGT
novel-miR-93	3121	AACTGTTTGCAGAGGAAACTGA
novel-miR-20	2785	TCCCTGAGACCCTAACTTGTGAT
novel-miR-94	2397	TGAGGTAGTAGATTGTATAGTTT
novel-miR-77	2336	TACAGTACTGTGATAACTGAAGA
novel-miR-72	1001	AGGCAAGATGCTGGCATAGCTGT
novel-miR-81	386	TAGCAGCGGGAACAGTACTGCAG
novel-miR-78	529	TATTGCACATTACTAAGTTGCA
novel-miR-70	417	TGAGATGAAGCACTGTAGCTC
novel-miR-37	491	GCCTGCTGGGGTGGAACCTGGT
novel-miR-83	266	AGAGGTAAAAAATTGATTTGACT
novel-miR-91	187	TGATTGGTACTTCTTAGAGTGA
novel-miR-27	204	TGAGACCTCCGGGTTCTGAGCT
novel-miR-68	225	GTGCCTACTGAGCTGAAACACA
novel-miR-38	312	TATGTGGGACGGTAAACCGCTT
novel-miR-36	176	GAGAGATCAGAGGCGCAGAGT
novel-miR-12	190	TGTTGTACTTTTTTTTTTGTTC
novel-miR-52	132	TGTAACAGCAACTCCATGTGGAA
novel-miR-71	134	GTCCAGTTTTCCCAGGAATCCCT
novel-miR-92	40	ATTGGCACGTCTTGGAATGCA
novel-miR-66	71	TCTGGTGCTTAGACTCTGTGCT
novel-miR-76	60	TGAGGTAGTAGATTGTATAGTT
novel-miR-56	82	AACTTTTGCCCCTAGTAACGGACT
novel-miR-75	73	TGAGGTAGTAGGTTGTATAGTTTT

novel-miR-62	57	ATGTCCGCGGGTTCCCTATCCC
novel-miR-14	48	TTCTCTGTTCATGTGGACGTGT
novel-miR-64	61	TGAGGTAGTAGTTTGTGCTGTT
novel-miR-55	46	GTACAGTACTGTGATAACTGA
novel-miR-9	52	GTTTCCGGTCGCTGTGCTCTCG
novel-miR-45	41	TGAGGTAGTAGTTTGTACAGTT
novel-miR-43	42	TCACAGTGAACCGGTCTCTTTC
novel-miR-21	37	TGAGGTAGTAGGTTGTATAGTT
novel-miR-13	31	GAAAGGTTCATTTGGGTTTTT
novel-miR-15	30	TTGCAGTGATGACTTGAGTCTG
novel-miR-31	24	ATCATGTATGATACTGCAAACA
novel-miR-41	29	TTGGACACTTCAGTACTGCTACA
novel-miR-67	32	TGCCCCGAGGCTTGTGAAGCTC
novel-miR-24	25	AAAACTCTGAATGAACTTTATG
novel-miR-35	16	TGAGGTAGTAGGTTGTATGGTT
novel-miR-48	29	TGGAAGGCCTGGCTTTGCAGCGT
novel-miR-65	18	TGAGGTAGTAGGTTGTGTGGTT
novel-miR-46	30	CTTAGAGCTTCCCCGCCTTGGGGA
novel-miR-69	13	CTCTGCCCGCTCTCTGTCTTACA
novel-miR-53	41	AAAACCTGAATGAACTTTTGAG
novel-miR-51	14	AAAACCTGAGTGAACTTTCTAG
novel-miR-39	14	CATCTGGTTGGTATGTTTTGCT
novel-miR-33	12	GCGGCCCGCGGGCTCGGATGCT
novel-miR-40	12	AAACCGACATGAACTTTTTGG
novel-miR-79	12	TGTTTGACATGAGAGCTTCCAGA
novel-miR-74	14	TGGCTCAGTTCAGCAGGAAC
novel-miR-11	11	GAAAACCTGAATGAACTTTTG
novel-miR-80	10	TTTAAGTACACGATGAACTGC
novel-miR-50	13	AAACCCGAATGAACTTTTTGA
novel-miR-6	9	AAAAACCTGAATGAACCTTTTG
novel-miR-61	10	CCTCATGTGGAATCTTTAGTTG
novel-miR-49	8	TAGAGAAGCACTGGGGGAAAGT
novel-miR-89	8	TGATTGGCATTTCTTAGAGTGA
novel-miR-4	9	TCTCAGGGTCTGTGAGCTCCTCG
novel-miR-16	8	TGGAATGTAAAGAAGTATGTAT
novel-miR-17	8	GCCCTGTCCTCCAGGAGCTC
novel-miR-7	6	AGGCCAAAGCAAACGACTGTGC
novel-miR-131	47	CCTATTCTTGATTACTTGTTTC
novel-miR-141	43	TGAGTAGTGCCTCTCTGAGTGT
novel-miR-121	40	ATTGTTCTCCAACCTGGCTCTTT
novel-miR-114	39	TCACTGGGCATCCTCTGCTTT
novel-miR-127	28	ATTGTCCTTGCTGTTTGGAGA
novel-miR-95	26	CCCAGGGATGTAGCTCCTAGTGC

novel-miR-107	24	TTTGCAGTAACAGGTGTGAAC
novel-miR-118	22	TAGCCAGTTGGGGAAGAATGC
novel-miR-134	22	TTTTCAGTGATTCAGCTCTAAGT
novel-miR-99	20	TGTAAACATCCTCGACTGGAAGC
novel-miR-132	16	CCTCACTGATGAGCAGCTTCTGAC
novel-miR-116	16	CTGCAGTGATGCCGCATCTGAGC
novel-miR-129	16	GAAGAATCTGAATGAACATTT
novel-miR-101	15	TTCTTGACTGCTCACCTGGGCT
novel-miR-105	14	TAATTTTTGCAAGGCTTTTCC
novel-miR-111	12	AACAGCGTGGATTTCAGTTGGCT
novel-miR-113	12	CTCGGAGAGTGGAGCTGTGGGTGT
novel-miR-123	11	ACCCTGTCCTCCAGGAGCTCA
novel-miR-108	10	TCTGAATCAGTCTATGTTGACT
novel-miR-97	10	TGCCAAGCCCACGTTCAAAG
novel-miR-140	10	AATGGTGCTTTTTTGTGAAGA
novel-miR-112	9	CAGTTTGCTCGGGCTTTTCTGT
novel-miR-135	9	AAAACCTGAACTAACTTTTTGA
novel-miR-106	9	GAAAACCTGAACAAACTTTGT
novel-miR-109	9	TTCTCATTGGCCTCACGTCCTG
novel-miR-104	9	TCCTCACTGTCTCTGCTCTGCAG
novel-miR-102	8	TTTCTTTGAGCTTCTTTCTTT
novel-miR-115	8	GTAGGGACCTCTCATGTCTGTG
novel-miR-137	8	GAAAAGTTCGTTTGGGTTTTCC
novel-miR-103	8	TGAGGTAGGAGGTTGTATAGTT
novel-miR-126	8	TACCCTGTAGAACCGAATTTGT
novel-miR-138	7	TGGTGCTCCCTGGAGCTGAGC
novel-miR-136	7	TTGCCTCCCGGAGGGCTGGAGC
novel-miR-98	7	AGCAGCATTGTACAGGGCTATGA
novel-miR-142	7	TGAGGTAGTAAGTTGTATTGTT
novel-miR-130	7	TGTAAACATCCTTGACTGGAAGCT
novel-miR-133	7	TAAAAGTTTGGTTGGGTTTTT
novel-miR-120	7	TGGTTTCAGGACAGATCTCTCC
novel-miR-117	7	CTGGTTTCAGGACAGATCTCT
novel-miR-119	6	TACGCCGGGAGCTCGCAGCG
novel-miR-122	5	AAACTTGAATGAACTTTCTGGCA
novel-miR-96	5	GAAAACCAAAACGAACTTTATG
novel-miR-125	5	TCTGAACGAACTTTGTGGCAA
novel-miR-110	5	GTAAACCTGAATGAACTTTTTG
novel-miR-139	5	TGTGACAGATTGATAACTGA
novel-miR-124	5	TCCCCCATGTGTCTCCTCGCCC
novel-miR-128	5	AAAAACCTGAATGAACCTTTTA
novel-miR-100	5	AAACCCAAATGAACTTCTTTGC

Supplementary Table S4

Differential expression of miRNAs in the ovaries of Tibetan goat (TBG) and Jintang black goat (JTG) during follicular phase

miR_name	TBG	JTG	log2 ratio (JTG/TBG) normalized	p-value	q-value
chi-miR-21-5p	158549	788052	2.179	0.000	0.000
chi-miR-379-5p	1201	4338	1.719	0.000	0.000
chi-miR-127-3p	3178	11169	1.679	0.000	0.000
chi-miR-199b-5p	6238	19222	1.490	0.000	0.000
chi-miR-199c-5p	6238	19222	1.490	0.000	0.000
chi-miR-200a	2275	303	-3.043	0.000	0.000
chi-miR-200b	1626	225	-2.987	0.000	0.000
chi-miR-204-5p	3139	1397	-1.302	0.000	0.000
chi-miR-411a-5p	1140	3020	1.271	0.000	0.000
chi-miR-493-5p	135	741	2.322	0.000	0.000
chi-miR-409-3p	203	873	1.970	0.000	0.000
chi-miR-196b	298	15	-4.446	0.000	0.000
chi-miR-483	407	66	-2.759	0.000	0.000
chi-miR-150	657	1467	1.025	0.000	0.000
chi-miR-424-3p	988	462	-1.231	0.000	0.000
chi-miR-493-3p	69	413	2.447	0.000	0.000
chi-miR-494	84	440	2.255	0.000	0.000
chi-miR-3958-3p	150	576	1.807	0.000	0.000
novel-miR-81	829	386	-1.293	0.000	0.000
chi-miR-136-3p	319	877	1.325	0.000	0.000
chi-miR-381	23	251	3.314	0.000	0.000
chi-miR-382-3p	120	451	1.776	0.000	0.000
novel-miR-38	62	312	2.141	0.000	0.000
chi-miR-136-5p	207	574	1.337	0.000	0.000
chi-miR-181d	330	105	-1.786	0.000	0.000
chi-miR-3959-5p	111	384	1.657	0.000	0.000
novel-mir-37	161	491	1.418	0.000	0.000
novel-mir-84	134	0	-8.257	0.000	0.000
chi-miR-592	357	142	-1.464	0.000	0.000
chi-miR-409-5p	60	243	1.884	0.000	0.000
chi-miR-127-5p	91	298	1.577	0.000	0.000
chi-miR-504	335	143	-1.362	0.000	0.000
chi-miR-135a	223	75	-1.706	0.000	0.000
chi-miR-196a	114	17	-2.880	0.000	0.000
chi-miR-432-5p	59	220	1.765	0.000	0.000
chi-miR-382-5p	32	163	2.215	0.000	0.000

chi-miR-411a-3p	48	178	1.757	0.000	0.000
chi-miR-380-3p	58	197	1.630	0.000	0.000
chi-miR-495-3p	140	339	1.142	0.000	0.000
chi-miR-369-3p	79	235	1.439	0.000	0.000
chi-miR-130b-5p	44	162	1.746	0.000	0.000
chi-miR-767	71	8	-3.284	0.000	0.000
novel-miR-90	57	0	-7.024	0.000	0.000
novel-miR-58	56	0	-6.998	0.000	0.000
novel-miR-63	56	0	-6.998	0.000	0.000
chi-miR-497-3p	2	56	4.673	0.000	0.000
chi-miR-34c-5p	185	78	-1.380	0.000	0.000
chi-miR-412-5p	42	144	1.644	0.000	0.000
chi-miR-429	57	7	-3.160	0.000	0.000
novel-miR-131	0	47	6.364	0.000	0.000
chi-miR-1249	109	38	-1.654	0.000	0.000
novel-miR-141	0	43	6.236	0.000	0.000
chi-miR-655	23	94	1.897	0.000	0.000
chi-miR-487b-3p	39	124	1.535	0.000	0.000
novel-miR-121	0	40	6.131	0.000	0.000
novel-miR-114	0	39	6.095	0.000	0.000
chi-miR-433	19	83	1.993	0.000	0.000
novel-miR-92	105	40	-1.583	0.000	0.000
chi-miR-21-3p	6	50	2.925	0.000	0.000
chi-miR-153	79	25	-1.794	0.000	0.000
chi-miR-105a	46	8	-2.658	0.000	0.000
chi-miR-223-3p	19	75	1.847	0.000	0.000
chi-miR-2318	2	32	3.866	0.000	0.000
chi-miR-204-3p	67	22	-1.741	0.000	0.000
novel-miR-32	25	0	-5.834	0.000	0.000
novel-miR-127	0	28	5.617	0.000	0.000
novel-miR-57	24	0	-5.776	0.000	0.000
chi-miR-369-5p	14	59	1.941	0.000	0.000
novel-miR-95	0	26	5.510	0.000	0.000
chi-miR-3955-5p	10	49	2.159	0.000	0.000
chi-miR-181c-5p	68	25	-1.578	0.000	0.000
novel-miR-107	0	24	5.394	0.000	0.000
novel-miR-3	21	0	-5.583	0.000	0.000
novel-miR-87	21	0	-5.583	0.000	0.000
chi-miR-450-3p	82	36	-1.322	0.000	0.000
novel-miR-53	7	41	2.360	0.000	0.000
novel-miR-118	0	22	5.269	0.000	0.000
novel-miR-134	0	22	5.269	0.000	0.000
chi-miR-27a-5p	31	84	1.304	0.000	0.000

chi-miR-379-3p	20	64	1.544	0.000	0.000
novel-miR-99	0	20	5.131	0.000	0.000
chi-miR-543-3p	49	111	1.046	0.000	0.000
chi-miR-154b-5p	16	55	1.647	0.000	0.000
chi-miR-200c	73	33	-1.280	0.000	0.000
chi-miR-181c-3p	67	29	-1.342	0.000	0.000
novel-miR-18	17	0	-5.278	0.000	0.000
chi-miR-485-3p	3	26	2.981	0.000	0.000
chi-miR-758	19	60	1.525	0.000	0.000
chi-miR-1197-3p	2	22	3.325	0.000	0.000
chi-miR-134	2	22	3.325	0.000	0.000
novel-miR-88	15	0	-5.098	0.000	0.000
chi-miR-411b-5p	32	78	1.151	0.000	0.000
novel-miR-116	0	16	4.809	0.000	0.000
novel-miR-129	0	16	4.809	0.000	0.000
novel-miR-132	0	16	4.809	0.000	0.000
chi-miR-374b-3p	82	44	-1.032	0.000	0.000
chi-miR-410-3p	14	46	1.582	0.000	0.000
chi-miR-665	4	25	2.510	0.000	0.000
chi-miR-376c-3p	18	53	1.424	0.000	0.000
novel-miR-101	0	15	4.716	0.000	0.000
novel-miR-30	13	0	-4.891	0.000	0.000
novel-miR-34	13	0	-4.891	0.000	0.000
chi-miR-383	10	37	1.753	0.000	0.000
chi-miR-135b-5p	25	6	-2.193	0.000	0.000
novel-miR-105	0	14	4.617	0.000	0.000
novel-miR-28	12	0	-4.776	0.000	0.000
novel-miR-29	12	0	-4.776	0.000	0.000
novel-miR-54	12	0	-4.776	0.000	0.000
chi-miR-485-5p	12	39	1.566	0.000	0.000
novel-miR-2	11	0	-4.650	0.000	0.000
novel-miR-42	11	0	-4.650	0.000	0.000
chi-miR-9-3p	21	5	-2.205	0.001	0.000
chi-miR-211	15	43	1.385	0.001	0.000
novel-miR-111	0	12	4.394	0.001	0.001
novel-miR-113	0	12	4.394	0.001	0.001
chi-miR-1248-5p	41	18	-1.322	0.001	0.001
novel-miR-123	0	11	4.269	0.001	0.001
chi-miR-223-5p	4	20	2.188	0.001	0.001
chi-miR-10a-3p	29	11	-1.533	0.002	0.001
chi-miR-376e-3p	27	60	1.018	0.002	0.001
novel-miR-10	9	0	-4.361	0.002	0.001
novel-miR-25	9	0	-4.361	0.002	0.001

novel-miR-47	9	0	-4.361	0.002	0.001
novel-miR-73	9	0	-4.361	0.002	0.001
novel-miR-82	9	0	-4.361	0.002	0.001
novel-miR-46	9	30	1.546	0.002	0.001
novel-miR-108	0	10	4.131	0.002	0.001
novel-miR-140	0	10	4.131	0.002	0.001
novel-miR-97	0	10	4.131	0.002	0.001
chi-miR-133b	13	36	1.335	0.002	0.002
chi-miR-338-5p	23	8	-1.658	0.003	0.002
novel-miR-5	8	0	-4.191	0.004	0.002
novel-miR-85	8	0	-4.191	0.004	0.002
novel-miR-104	0	9	3.979	0.004	0.002
novel-miR-106	0	9	3.979	0.004	0.002
novel-miR-109	0	9	3.979	0.004	0.002
novel-miR-112	0	9	3.979	0.004	0.002
novel-miR-135	0	9	3.979	0.004	0.002
novel-miR-19	7	0	-3.998	0.007	0.004
novel-miR-59	7	0	-3.998	0.007	0.004
novel-miR-102	0	8	3.809	0.008	0.004
novel-miR-103	0	8	3.809	0.008	0.004
novel-miR-115	0	8	3.809	0.008	0.004
novel-miR-126	0	8	3.809	0.008	0.004
novel-miR-137	0	8	3.809	0.008	0.004
novel-miR-67	12	32	1.224	0.008	0.004
chi-miR-224-3p	11	2	-2.594	0.006	0.004
chi-miR-323b	16	38	1.114	0.007	0.004
novel-miR-48	11	29	1.208	0.012	0.005
novel-miR-117	0	7	3.617	0.015	0.006
novel-miR-120	0	7	3.617	0.015	0.006
novel-miR-130	0	7	3.617	0.015	0.006
novel-miR-133	0	7	3.617	0.015	0.006
novel-miR-136	0	7	3.617	0.015	0.006
novel-miR-138	0	7	3.617	0.015	0.006
novel-miR-142	0	7	3.617	0.015	0.006
novel-miR-98	0	7	3.617	0.015	0.006
novel-miR-1	6	0	-3.776	0.015	0.006
novel-miR-22	6	0	-3.776	0.015	0.006
novel-miR-23	6	0	-3.776	0.015	0.006
novel-miR-26	6	0	-3.776	0.015	0.006
novel-miR-60	6	0	-3.776	0.015	0.006
chi-miR-1185-3p	7	22	1.518	0.009	0.006
chi-miR-141	14	4	-1.941	0.009	0.006
chi-miR-376b-3p	4	16	1.866	0.010	0.006

chi-miR-23b-5p	8	1	-3.134	0.010	0.006
chi-miR-582-5p	11	28	1.214	0.013	0.008
novel-miR-119	0	6	3.394	0.028	0.010
novel-miR-44	5	0	-3.513	0.030	0.011
novel-miR-86	5	0	-3.513	0.030	0.011
novel-miR-11	21	11	-1.124	0.032	0.011
chi-miR-154a-3p	4	14	1.673	0.025	0.015
novel-miR-100	0	5	3.131	0.053	0.017
novel-miR-110	0	5	3.131	0.053	0.017
novel-miR-122	0	5	3.131	0.053	0.017
novel-miR-124	0	5	3.131	0.053	0.017
novel-miR-125	0	5	3.131	0.053	0.017
novel-miR-128	0	5	3.131	0.053	0.017
novel-miR-139	0	5	3.131	0.053	0.017
novel-miR-96	0	5	3.131	0.053	0.017
chi-miR-380-5p	1	7	2.673	0.033	0.020
chi-miR-338-3p	24	13	-1.019	0.036	0.021
chi-miR-376d	2	9	2.036	0.040	0.024
chi-miR-2331	3	11	1.740	0.041	0.024
chi-miR-34b-3p	17	8	-1.222	0.041	0.024
chi-miR-656	0	5	3.188	0.049	0.028
chi-miR-192-3p	1	6	2.451	0.060	0.034
chi-miR-329a-3p	1	6	2.451	0.060	0.034
chi-miR-105b-3p	7	2	-1.941	0.066	0.037
chi-miR-3959-3p	3	10	1.603	0.066	0.037
chi-miR-34c-3p	5	1	-2.456	0.072	0.040

Supplementary Table S5

Target gene prediction for miRNAs from Tibetan (TBG) and Jintang black (JTG) goats

miRNA types	Breeds	Software	miRNA_ number	Target_gene_ number	miRNA::target_ number	Target_location_ number
Known miRNA	TBG	miRanda	388	29,491	1,815,017	2,276,364
		PITA	388	29,496	9,581,600	45,478,007
		targetscan	388	29,496	2,749,564	3,860,577
		Result	388	29,489	1,807,176	-
	JTG	miRanda	389	29,490	1,804,613	2,257,347
		PITA	389	29,496	9,609,012	45,701,734
		targetscan	389	29,496	2,742,221	3,843,082
		Result	389	29,489	1,796,868	-
Novel miRNA	TBG	miRanda	94	29,275	452,035	564,625
		PITA	94	29,496	2,370,690	11,633,916
		targetscan	94	29,445	684,787	966,694
		Result	94	29,260	449,709	-
	JTG	miRanda	109	29,328	530,446	661,664
		PITA	109	29,496	2,724,945	13,428,697
		targetscan	109	29,450	805,088	1,133,346
		Result	109	29,314	527,684	-

Supplementary Table S6

Terms from the function Ontology with p-value as good or better than 1 DEGs predicted from the novel miRNAs

Gene Ontology term	Cluster frequency	Genome frequency of use	Corrected P-value
transferase activity	1685/10189, 16.5%	1704/10487, 16.2%	0.000
metal ion binding	2025/10189, 19.9%	2052/10487, 19.6%	0.000
catalytic activity	4596 /10189, 45.1%	4690/10487, 44.7%	0.001
small molecule binding	1705/10189, 16.7%	1729/10487, 16.5%	0.009
nucleotide binding	1612/10189, 15.8%	1634/10487, 15.6%	0.010
nucleoside phosphate binding	1612/10189, 15.8%	1634/10487, 15.6%	0.010
purine nucleotide binding	1482/10189, 14.5%	1502/10487, 14.3%	0.019
ribonucleotide binding	1478/10189, 14.5%	1498/10487, 14.3%	0.021
purine ribonucleotide binding	1478/10189, 14.5%	1498/10487, 14.3%	0.021
adenyl nucleotide binding	1118/10189, 11.0%	1131/10487, 10.8%	0.035
adenyl ribonucleotide binding	1116/10189, 11.0%	1129/10487, 10.8%	0.036
carbohydrate derivative binding	1570/10189, 15.4%	1593/10487, 15.2%	0.046
transition metal ion binding	1472/10189, 14.4%	1494/10487, 14.2%	0.116
ion binding	2702/10189, 26.5%	2755/10487, 26.3%	0.226
cation binding	2532/10189, 24.9%	2582/10487, 24.6%	0.457
protein kinase activity	393/10189, 3.9%	395/10487, 3.8%	0.577
phosphotransferase activity, alcohol group as acceptor	430/10189, 4.2%	433/10487, 4.1%	1.000
hydrolase activity	1679/10189, 16.5%	1710/10487, 16.3%	1.000
ligase activity	185/10189, 1.8%	185/10487, 1.8%	1.000
kinase activity	555/10189, 5.4%	562/10487, 5.4%	1.000
channel activity	228/10189, 2.2%	229/10487, 2.2%	1.000
passive transmembrane transporter activity	228/10189, 2.2%	229/10487, 2.2%	1.000
enzyme binding	287/10189, 2.8%	289/10487, 2.8%	1.000
ion channel activity	218/10189, 2.1%	219/10487, 2.1%	1.000
substrate-specific channel activity	218/10189, 2.1%	219/10487, 2.1%	1.000
binding	7056/10189, 69.3%	7244/10487, 69.1%	1.000
transferase activity, transferring phosphorus-containing groups	784/10189, 7.7%	797/10487, 7.6%	1.000
hydrolase activity, acting on acid anhydrides	608/10189, 6.0%	617/10487, 5.9%	1.000
hydrolase activity, acting on acid anhydrides, in phosphorus-containing anhydrides	606/10189, 5.9%	615/10487, 5.9%	1.000
pyrophosphatase activity	602/10189, 5.9%	611/10487, 5.8%	1.000
transferase activity, transferring glycosyl groups	194/10189, 1.9%	195/10487, 1.9%	1.000
organic cyclic compound binding	2985/10189, 29.3%	3057/10487, 29.2%	1.000

transcription cofactor activity	119/10189, 1.2%	119/10487, 1.1%	1.000
protein serine/threonine kinase activity	116/10189, 1.1%	116/10487, 1.1%	1.000
secondary active transmembrane transporter activity	116/10189, 1.1%	116/10487, 1.1%	1.000
heterocyclic compound binding	2960/10189, 29.1%	3032/10487, 28.9%	1.000
protein dimerization activity	113/10189, 1.1%	113/10487, 1.1%	1.000
oxidoreductase activity, acting on CH-OH group of donors	110/10189, 1.1%	110/10487, 1.0%	1.000
coenzyme binding	109/10189, 1.1%	109/10487, 1.0%	1.000
hydrolase activity, acting on glycosyl bonds	105/10189, 1.0%	105/10487, 1.0%	1.000
transferase activity, transferring one-carbon groups	162/10189, 1.6%	163/10487, 1.6%	1.000
peptidase activity	445/10189, 4.4%	452/10487, 4.3%	1.000
transmembrane transporter activity	612/10189, 6.0%	623/10487, 5.9%	1.000
peptidase activity, acting on L-amino acid peptides	439/10189, 4.3%	446/10487, 4.3%	1.000
lipid binding	207/10189, 2.0%	209/10487, 2.0%	1.000
nucleoside-triphosphatase activity	520/10189, 5.1%	529/10487, 5.0%	1.000
methyltransferase activity	95/10189, 0.9%	95/10487, 0.9%	1.000
active transmembrane transporter activity	203/10189, 2.0%	205/10487, 2.0%	1.000
kinase binding	91/10189, 0.9%	91/10487, 0.9%	1.000
transferase activity, transferring acyl groups	196/10189, 1.9%	198/10487, 1.9%	1.000
anion transmembrane transporter activity	89/10189, 0.9%	89/10487, 0.8%	1.000
metal ion transmembrane transporter activity	144/10189, 1.4%	145/10487, 1.4%	1.000
ligase activity, forming carbon-nitrogen bonds	87/10189, 0.9%	87/10487, 0.8%	1.000
peptide receptor activity	83/10189, 0.8%	83/10487, 0.8%	1.000
lyase activity	137/10189, 1.3%	138/10487, 1.3%	1.000
transporter activity	727/10189, 7.1%	742/10487, 7.1%	1.000
G-protein coupled peptide receptor activity	80/10189, 0.8%	80/10487, 0.8%	1.000
oxidoreductase activity, acting on the CH-OH group of donors, NAD or NADP as acceptor	79/10189, 0.8%	79/10487, 0.8%	1.000
ATPase activity	181/10189, 1.8%	183/10487, 1.7%	1.000
protein binding transcription factor activity	130/10189, 1.3%	131/10487, 1.2%	1.000
transcription factor binding transcription factor activity	130/10189, 1.3%	131/10487, 1.2%	1.000
ATPase activity, coupled	177/10189, 1.7%	179/10487, 1.7%	1.000
thiolester hydrolase activity	75/10189, 0.7%	75/10487, 0.7%	1.000
transferase activity, transferring acyl groups other than amino-acyl groups	176/10189, 1.7%	178/10487, 1.7%	1.000
hydrolase activity, hydrolyzing O-glycosyl	73/10189, 0.7%	73/10487, 0.7%	1.000

compounds			
ion transmembrane transporter activity	469/10189, 4.6%	478/10487, 4.6%	1.000
cation channel activity	125/10189, 1.2%	126/10487, 1.2%	1.000
molecular transducer activity	932/10189, 9.1%	953/10487, 9.1%	1.000
potassium ion transmembrane transporter activity	71/10189, 0.7%	71/10487, 0.7%	1.000
cofactor binding	213/10189, 2.1%	216/10487, 2.1%	1.000
guanyl nucleotide binding	381/10189, 3.7%	388/10487, 3.7%	1.000
guanyl ribonucleotide binding	380/10189, 3.7%	387/10487, 3.7%	1.000
oxidoreductase activity, acting on the CH-CH group of donors	69/10189, 0.7%	69/10487, 0.7%	1.000
substrate-specific transmembrane transporter activity	538/10189, 5.3%	549/10487, 5.2%	1.000
tubulin binding	68/10189, 0.7%	68/10487, 0.6%	1.000
endopeptidase activity	295/10189, 2.9%	300/10487, 2.9%	1.000
UDP-glycosyltransferase activity	66/10189, 0.6%	66/10487, 0.6%	1.000
ligand-gated ion channel activity	65/10189, 0.6%	65/10487, 0.6%	1.000
ligand-gated channel activity	65/10189, 0.6%	65/10487, 0.6%	1.000
potassium channel activity	63/10189, 0.6%	63/10487, 0.6%	1.000
N-acyltransferase activity	61/10189, 0.6%	61/10487, 0.6%	1.000
oxidoreductase activity, acting on paired donors, with incorporation or reduction of molecular oxygen	109/10189, 1.1%	110/10487, 1.0%	1.000
sequence-specific DNA binding	107/10189, 1.1%	108/10487, 1.0%	1.000
ligase activity, forming carbon-oxygen bonds	58/10189, 0.6%	58/10487, 0.6%	1.000
ligase activity, forming aminoacyl-tRNA and related compounds	58/10189, 0.6%	58/10487, 0.6%	1.000
receptor activity	586/10189, 5.8%	599/10487, 5.7%	1.000
hydrolase activity, acting on ester bonds	466/10189, 4.6%	476/10487, 4.5%	1.000
cytoskeletal protein binding	268/10189, 2.6%	273/10487, 2.6%	1.000
nucleic acid binding transcription factor activity	459/10189, 4.5%	469/10487, 4.5%	1.000
aminoacyl-tRNA ligase activity	53/10189, 0.5%	53/10487, 0.5%	1.000
phospholipid binding	99/10189, 1.0%	100/10487, 1.0%	1.000
carbon-oxygen lyase activity	52/10189, 0.5%	52/10487, 0.5%	1.000
dioxygenase activity	52/10189, 0.5%	52/10487, 0.5%	1.000
gated channel activity	97/10189, 1.0%	98/10487, 0.9%	1.000
substrate-specific transporter activity	638/10189, 6.3%	653/10487, 6.2%	1.000
small conjugating protein transferase activity	179/10189, 1.8%	182/10487, 1.7%	1.000
signal transducer activity	784/10189, 7.7%	803/10487, 7.7%	1.000
transferase activity, transferring	49/10189, 0.5%	49/10487, 0.5%	1.000

sulfur-containing groups			
hormone receptor binding	49/10189, 0.5%	49/10487, 0.5%	1.000
oxidoreductase activity, acting on single donors with incorporation of molecular O_2	48/10189, 0.5%	48/10487, 0.5%	1.000
protein tyrosine kinase activity	47/10189, 0.5%	47/10487, 0.4%	1.000
transferase activity, transferring hexosyl groups	91/10189, 0.9%	92/10487, 0.9%	1.000
organic acid transmembrane transporter activity	46/10189, 0.5%	46/10487, 0.4%	1.000
N-acetyltransferase activity	46/10189, 0.5%	46/10487, 0.4%	1.000
transferase activity, transferring alkyl or aryl (other than methyl) groups	45/10189, 0.4%	45/10487, 0.4%	1.000
carbohydrate binding	129/10189, 1.3%	131/10487, 1.2%	1.000
ligand-activated sequence-specific DNA binding RNA polymerase II transcription factor activity	44/10189, 0.4%	44/10487, 0.4%	1.000
S-adenosylmethionine-dependent methyltransferase activity	44/10189, 0.4%	44/10487, 0.4%	1.000
carbon-carbon lyase activity	44/10189, 0.4%	44/10487, 0.4%	1.000
direct ligand regulated sequence-specific DNA binding transcription factor activity	44/10189, 0.4%	44/10487, 0.4%	1.000
small GTPase regulator activity	85/10189, 0.8%	86/10487, 0.8%	1.000
sodium ion transmembrane transporter activity	42/10189, 0.4%	42/10487, 0.4%	1.000
cysteine-type peptidase activity	40/10189, 0.4%	40/10487, 0.4%	1.000
transferase activity, transferring nitrogenous groups	40/10189, 0.4%	40/10487, 0.4%	1.000
guanyl-nucleotide exchange factor activity	39/10189, 0.4%	39/10487, 0.4%	1.000
cation-transporting ATPase activity	39/10189, 0.4%	39/10487, 0.4%	1.000
phosphoric ester hydrolase activity	232/10189, 2.3%	237/10487, 2.3%	1.000
motor activity	38/10189, 0.4%	38/10487, 0.4%	1.000
Ras guanyl-nucleotide exchange factor activity	38/10189, 0.4%	38/10487, 0.4%	1.000
oxidoreductase activity, acting on paired donors, with incorporation or reduction of molecular O_2, 2-oxoglutarate as one donor, and incorporation of one atom each of O_2 into both donors	37/10189, 0.4%	37/10487, 0.4%	1.000
transferase activity, transferring pentosyl groups	37/10189, 0.4%	37/10487, 0.4%	1.000
ligase activity, forming carbon-sulfur bonds	37/10189, 0.4%	37/10487, 0.4%	1.000
vitamin binding	37/10189, 0.4%	37/10487, 0.4%	1.000
primary active transmembrane transporter	77/10189, 0.8%	78/10487, 0.7%	1.000

activity			
P-P-bond-hydrolysis-driven transmembrane transporter activity	77/10189, 0.8%	78/10487, 0.7%	1.000
signaling receptor activity	516/10189, 5.1%	529/10487, 5.0%	1.000
sequence-specific DNA binding transcription factor activity	75/10189, 0.7%	76/10487, 0.7%	1.000
anion channel activity	35/10189, 0.3%	35/10487, 0.3%	1.000
nuclear hormone receptor binding	35/10189, 0.3%	35/10487, 0.3%	1.000
monooxygenase activity	74/10189, 0.7%	75/10487, 0.7%	1.000
sequence-specific DNA binding RNA polymerase II transcription factor activity	73/10189, 0.7%	74/10487, 0.7%	1.000
actin binding	34/10189, 0.3%	34/10487, 0.3%	1.000
phosphoric diester hydrolase activity	34/10189, 0.3%	34/10487, 0.3%	1.000
organic anion transmembrane transporter activity	34/10189, 0.3%	34/10487, 0.3%	1.000
carboxylic acid transmembrane transporter activity	34/10189, 0.3%	34/10487, 0.3%	1.000
phosphatidylinositol binding	33/10189, 0.3%	33/10487, 0.3%	1.000
cation transmembrane transporter activity	290/10189, 2.8%	297/10487, 2.8%	1.000
transmembrane signaling receptor activity	501/10189, 4.9%	514/10487, 4.9%	1.000
lysine N-acetyltransferase activity, acting on acetyl phosphate as donor	32/10189, 0.3%	32/10487, 0.3%	1.000
intramolecular transferase activity	32/10189, 0.3%	32/10487, 0.3%	1.000
RNA binding	286/10189, 2.8%	293/10487, 2.8%	1.000
N-methyltransferase activity	31/10189, 0.3%	31/10487, 0.3%	1.000
symporter activity	31/10189, 0.3%	31/10487, 0.3%	1.000
solute:cation symporter activity	31/10189, 0.3%	31/10487, 0.3%	1.000
lipase activity	31/10189, 0.3%	31/10487, 0.3%	1.000
hydro-lyase activity	31/10189, 0.3%	31/10487, 0.3%	1.000
small conjugating protein-specific protease activity	31/10189, 0.3%	31/10487, 0.3%	1.000
hydrolase activity, acting on acid anhydrides, catalyzing transmembrane movement of substances	68/10189, 0.7%	69/10487, 0.7%	1.000
ATPase activity, coupled to transmembrane movement of substances	67/10189, 0.7%	68/10487, 0.6%	1.000
ATPase activity, coupled to movement of substances	67/10189, 0.7%	68/10487, 0.6%	1.000
transmembrane receptor protein kinase activity	29/10189, 0.3%	29/10487, 0.3%	1.000
phosphatase binding	29/10189, 0.3%	29/10487, 0.3%	1.000
anion binding	172/10189, 1.7%	176/10487, 1.7%	1.000
protein complex binding	65/10189, 0.6%	66/10487, 0.6%	1.000

transcription factor binding	64/10189, 0.6%	65/10487, 0.6%	1.000
exopeptidase activity	64/10189, 0.6%	65/10487, 0.6%	1.000
mRNA binding	28/10189, 0.3%	28/10487, 0.3%	1.000
macrolide binding	28/10189, 0.3%	28/10487, 0.3%	1.000
drug binding	28/10189, 0.3%	28/10487, 0.3%	1.000
oxidoreductase activity, acting on the aldehyde or oxo group of donors	28/10189, 0.3%	28/10487, 0.3%	1.000
neurotransmitter receptor activity	28/10189, 0.3%	28/10487, 0.3%	1.000
poly(A) RNA binding	28/10189, 0.3%	28/10487, 0.3%	1.000
acetyltransferase activity	63/10189, 0.6%	64/10487, 0.6%	1.000
hydrolase activity, acting on carbon-nitrogen (but not peptide) bonds	63/10189, 0.6%	64/10487, 0.6%	1.000
ATPase activity, coupled to transmembrane movement of ions	63/10189, 0.6%	64/10487, 0.6%	1.000
pattern binding	27/10189, 0.3%	27/10487, 0.3%	1.000
helicase activity	27/10189, 0.3%	27/10487, 0.3%	1.000
phospholipase activity	27/10189, 0.3%	27/10487, 0.3%	1.000
extracellular ligand-gated ion channel activity	27/10189, 0.3%	27/10487, 0.3%	1.000
amino acid transmembrane transporter activity	27/10189, 0.3%	27/10487, 0.3%	1.000
carboxy-lyase activity	27/10189, 0.3%	27/10487, 0.3%	1.000
DNA polymerase activity	27/10189, 0.3%	27/10487, 0.3%	1.000
excitatory extracellular ligand-gated ion channel activity	26/10189, 0.3%	26/10487, 0.2%	1.000
steroid binding	26/10189, 0.3%	26/10487, 0.2%	1.000
acetylglucosaminyltransferase activity	26/10189, 0.3%	26/10487, 0.2%	1.000
adenylyltransferase activity	26/10189, 0.3%	26/10487, 0.2%	1.000
G-protein coupled chemoattractant receptor activity	25/10189, 0.2%	25/10487, 0.2%	1.000
nucleoside binding	25/10189, 0.2%	25/10487, 0.2%	1.000
sulfotransferase activity	25/10189, 0.2%	25/10487, 0.2%	1.000
protein-lipid complex binding	25/10189, 0.2%	25/10487, 0.2%	1.000
solute:sodium symporter activity	24/10189, 0.2%	24/10487, 0.2%	1.000
steroid dehydrogenase activity	24/10189, 0.2%	24/10487, 0.2%	1.000
GTPase binding	24/10189, 0.2%	24/10487, 0.2%	1.000
lipoprotein particle binding	24/10189, 0.2%	24/10487, 0.2%	1.000
endopeptidase regulator activity	124/10189, 1.2%	127/10487, 1.2%	1.000
deoxyribonuclease activity	23/10189, 0.2%	23/10487, 0.2%	1.000
neuropeptide receptor activity	23/10189, 0.2%	23/10487, 0.2%	1.000
G-protein coupled amine receptor activity	23/10189, 0.2%	23/10487, 0.2%	1.000
protein methyltransferase activity	23/10189, 0.2%	23/10487, 0.2%	1.000
hydrolase activity, acting on	23/10189, 0.2%	23/10487, 0.2%	1.000

carbon-nitrogen (but not peptide) bonds, in linear amides			
acid-amino acid ligase activity	23/10189, 0.2%	23/10487, 0.2%	1.000
G-protein coupled nucleotide receptor activity	22/10189, 0.2%	22/10487, 0.2%	1.000
transcription coactivator activity	22/10189, 0.2%	22/10487, 0.2%	1.000
fatty acid binding	22/10189, 0.2%	22/10487, 0.2%	1.000
oxidoreductase activity, acting on paired donors, with incorporation or reduction of molecular oxygen, NAD(P)H as one donor, and incorporation of one atom of oxygen	22/10189, 0.2%	22/10487, 0.2%	1.000
monocarboxylic acid binding	22/10189, 0.2%	22/10487, 0.2%	1.000
steroid hormone receptor binding	22/10189, 0.2%	22/10487, 0.2%	1.000
translation factor activity, nucleic acid binding	86/10189, 0.8%	88/10487, 0.8%	1.000
GTPase regulator activity	119/10189, 1.2%	122/10487, 1.2%	1.000
nucleoside-triphosphatase regulator activity	119/10189, 1.2%	122/10487, 1.2%	1.000
phosphatase activity	185/10189, 1.8%	190/10487, 1.8%	1.000
receptor signaling protein activity	21/10189, 0.2%	21/10487, 0.2%	1.000
transaminase activity	21/10189, 0.2%	21/10487, 0.2%	1.000
structure-specific DNA binding	84/10189, 0.8%	86/10487, 0.8%	1.000
purine nucleoside binding	20/10189, 0.2%	20/10487, 0.2%	1.000
oxidoreductase activity, acting on the CH-NH group of donors, NAD or NADP as acceptor	20/10189, 0.2%	20/10487, 0.2%	1.000
carbon-sulfur lyase activity	20/10189, 0.2%	20/10487, 0.2%	1.000
GABA receptor activity	20/10189, 0.2%	20/10487, 0.2%	1.000
protein kinase binding	20/10189, 0.2%	20/10487, 0.2%	1.000
ribonucleoside binding	20/10189, 0.2%	20/10487, 0.2%	1.000
purine ribonucleoside binding	20/10189, 0.2%	20/10487, 0.2%	1.000
cell adhesion molecule binding	20/10189, 0.2%	20/10487, 0.2%	1.000
DNA binding	411/10189, 4.0%	423/10487, 4.0%	1.000
endodeoxyribonuclease activity	19/10189, 0.2%	19/10487, 0.2%	1.000
chemokine receptor activity	19/10189, 0.2%	19/10487, 0.2%	1.000
organic acid:sodium symporter activity	19/10189, 0.2%	19/10487, 0.2%	1.000
inorganic anion transmembrane transporter activity	19/10189, 0.2%	19/10487, 0.2%	1.000
oxidoreductase activity, acting on the aldehyde or oxo group of donors, NAD or NADP as acceptor	19/10189, 0.2%	19/10487, 0.2%	1.000
Ras GTPase binding	19/10189, 0.2%	19/10487, 0.2%	1.000
small GTPase binding	19/10189, 0.2%	19/10487, 0.2%	1.000
monosaccharide binding	19/10189, 0.2%	19/10487, 0.2%	1.000

protein binding	2186/10189, 21.5%	2251/10487, 21.5%	1.000
endopeptidase inhibitor activity	111/10189, 1.1%	114/10487, 1.1%	1.000
receptor signaling protein serine/threonine kinase activity	18/10189, 0.2%	18/10487, 0.2%	1.000
neurotransmitter transporter activity	18/10189, 0.2%	18/10487, 0.2%	1.000
galactosyltransferase activity	18/10189, 0.2%	18/10487, 0.2%	1.000
intramolecular oxidoreductase activity, interconverting aldoses and ketoses	18/10189, 0.2%	18/10487, 0.2%	1.000
protein phosphatase regulator activity	18/10189, 0.2%	18/10487, 0.2%	1.000
steroid dehydrogenase activity, acting on the CH-OH group of donors, NAD or NADP as acceptor	18/10189, 0.2%	18/10487, 0.2%	1.000
alcohol binding	18/10189, 0.2%	18/10487, 0.2%	1.000
RNA methyltransferase activity	17/10189, 0.2%	17/10487, 0.2%	1.000
antiporter activity	17/10189, 0.2%	17/10487, 0.2%	1.000
oxidoreductase activity, acting on the CH-CH group of donors, NAD or NADP as acceptor	17/10189, 0.2%	17/10487, 0.2%	1.000
polysaccharide binding	17/10189, 0.2%	17/10487, 0.2%	1.000
sterol binding	17/10189, 0.2%	17/10487, 0.2%	1.000
UDP-galactosyltransferase activity	17/10189, 0.2%	17/10487, 0.2%	1.000
histone binding	17/10189, 0.2%	17/10487, 0.2%	1.000
cyclic-nucleotide phosphodiesterase activity	16/10189, 0.2%	16/10487, 0.2%	1.000
transmembrane receptor protein serine/threonine kinase activity	16/10189, 0.2%	16/10487, 0.2%	1.000
carbohydrate transmembrane transporter activity	16/10189, 0.2%	16/10487, 0.2%	1.000
growth factor receptor binding	16/10189, 0.2%	16/10487, 0.2%	1.000
carbohydrate transporter activity	16/10189, 0.2%	16/10487, 0.2%	1.000
nucleotidase activity	15/10189, 0.1%	15/10487, 0.1%	1.000
mannosidase activity	15/10189, 0.1%	15/10487, 0.1%	1.000
lysine N-methyltransferase activity	15/10189, 0.1%	15/10487, 0.1%	1.000
protein-lysine N-methyltransferase activity	15/10189, 0.1%	15/10487, 0.1%	1.000
CoA-ligase activity	15/10189, 0.1%	15/10487, 0.1%	1.000
oxidoreductase activity, acting on single donors with incorporation of molecular oxygen, incorporation of two atoms of O_2	15/10189, 0.1%	15/10487, 0.1%	1.000
GTPase activator activity	42/10189, 0.4%	43/10487, 0.4%	1.000
Ras GTPase activator activity	42/10189, 0.4%	43/10487, 0.4%	1.000
chromatin binding	14/10189, 0.1%	14/10487, 0.1%	1.000
sialyltransferase activity	14/10189, 0.1%	14/10487, 0.1%	1.000
nucleobase-containing compound transmembrane transporter activity	14/10189, 0.1%	14/10487, 0.1%	1.000

GMP binding	14/10189, 0.1%	14/10487, 0.1%	1.000
G-protein coupled receptor activity	252/10189, 2.5%	260/10487, 2.5%	1.000
transmembrane receptor protein tyrosine kinase activity	13/10189, 0.1%	13/10487, 0.1%	1.000
signal sequence binding	13/10189, 0.1%	13/10487, 0.1%	1.000
voltage-gated potassium channel activity	13/10189, 0.1%	13/10487, 0.1%	1.000
chloride transmembrane transporter activity	13/10189, 0.1%	13/10487, 0.1%	1.000
sterol transporter activity	13/10189, 0.1%	13/10487, 0.1%	1.000
intramolecular transferase activity, phosphotransferases	13/10189, 0.1%	13/10487, 0.1%	1.000
carbohydrate phosphatase activity	13/10189, 0.1%	13/10487, 0.1%	1.000
wide pore channel activity	13/10189, 0.1%	13/10487, 0.1%	1.000
heat shock protein binding	13/10189, 0.1%	13/10487, 0.1%	1.000
sugar transmembrane transporter activity	13/10189, 0.1%	13/10487, 0.1%	1.000
macromolecular complex binding	125/10189, 1.2%	129/10487, 1.2%	1.000
antioxidant activity	38/10189, 0.4%	39/10487, 0.4%	1.000
intramolecular oxidoreductase activity	37/10189, 0.4%	38/10487, 0.4%	1.000
enzyme inhibitor activity	153/10189, 1.5%	158/10487, 1.5%	1.000
prenyltransferase activity	12/10189, 0.1%	12/10487, 0.1%	1.000
protein histidine kinase activity	12/10189, 0.1%	12/10487, 0.1%	1.000
chloride channel activity	12/10189, 0.1%	12/10487, 0.1%	1.000
steroid hydroxylase activity	12/10189, 0.1%	12/10487, 0.1%	1.000
small protein activating enzyme activity	12/10189, 0.1%	12/10487, 0.1%	1.000
C-acyltransferase activity	12/10189, 0.1%	12/10487, 0.1%	1.000
hydroxymethyl-, formyl- and related transferase activity	12/10189, 0.1%	12/10487, 0.1%	1.000
phosphotransferase activity, nitrogenous group as acceptor	12/10189, 0.1%	12/10487, 0.1%	1.000
hydrolase activity, hydrolyzing N-glycosyl compounds	12/10189, 0.1%	12/10487, 0.1%	1.000
acid-thiol ligase activity	12/10189, 0.1%	12/10487, 0.1%	1.000
endonuclease activity, active with either ribo- or deoxyribonucleic acids and producing 5'-phosphomonoesters	12/10189, 0.1%	12/10487, 0.1%	1.000
rRNA binding	12/10189, 0.1%	12/10487, 0.1%	1.000
MHC protein binding	12/10189, 0.1%	12/10487, 0.1%	1.000
retinoic acid receptor binding	12/10189, 0.1%	12/10487, 0.1%	1.000
SMAD binding	12/10189, 0.1%	12/10487, 0.1%	1.000
NAD binding	12/10189, 0.1%	12/10487, 0.1%	1.000
enzyme regulator activity	433/10189, 4.2%	447/10487, 4.3%	1.000
O-acyltransferase activity	36/10189, 0.4%	37/10487, 0.4%	1.000
amide binding	36/10189, 0.4%	37/10487, 0.4%	1.000
peptide binding	36/10189, 0.4%	37/10487, 0.4%	1.000

inorganic cation transmembrane transporter activity	209/10189, 2.1%	216/10487, 2.1%	1.000
DNA helicase activity	11/10189, 0.1%	11/10487, 0.1%	1.000
intracellular ligand-gated ion channel activity	11/10189, 0.1%	11/10487, 0.1%	1.000
hexosaminidase activity	11/10189, 0.1%	11/10487, 0.1%	1.000
nucleotide receptor activity	11/10189, 0.1%	11/10487, 0.1%	1.000
phosphotransferase activity, for other substituted phosphate groups	11/10189, 0.1%	11/10487, 0.1%	1.000
cyclo-ligase activity	11/10189, 0.1%	11/10487, 0.1%	1.000
carbohydrate kinase activity	11/10189, 0.1%	11/10487, 0.1%	1.000
receptor activator activity	11/10189, 0.1%	11/10487, 0.1%	1.000
phosphatidylinositol 3-kinase activity	11/10189, 0.1%	11/10487, 0.1%	1.000
transition metal ion transmembrane transporter activity	11/10189, 0.1%	11/10487, 0.1%	1.000
peptidase inhibitor activity	118/10189, 1.2%	122/10487, 1.2%	1.000
cytokine receptor activity	33/10189, 0.3%	34/10487, 0.3%	1.000
phosphotransferase activity, phosphate group as acceptor	33/10189, 0.3%	34/10487, 0.3%	1.000
3',5'-cyclic-nucleotide phosphodiesterase activity	10/10189, 0.1%	10/10487, 0.1%	1.000
calcium activated cation channel activity	10/10189, 0.1%	10/10487, 0.1%	1.000
dicarboxylic acid transmembrane transporter activity	10/10189, 0.1%	10/10487, 0.1%	1.000
metalloexopeptidase activity	10/10189, 0.1%	10/10487, 0.1%	1.000
ion gated channel activity	10/10189, 0.1%	10/10487, 0.1%	1.000
NADP binding	10/10189, 0.1%	10/10487, 0.1%	1.000
kinase regulator activity	58/10189, 0.6%	60/10487, 0.6%	1.000
monovalent inorganic cation transmembrane transporter activity	171/10189, 1.7%	177/10487, 1.7%	1.000
aldo-keto reductase (NADP) activity	9/10189, 0.1%	9/10487, 0.1%	1.000
cysteine-type endopeptidase activity	9/10189, 0.1%	9/10487, 0.1%	1.000
phospholipase C activity	9/10189, 0.1%	9/10487, 0.1%	1.000
ubiquitin-protein transferase activity	9/10189, 0.1%	9/10487, 0.1%	1.000
protein kinase inhibitor activity	9/10189, 0.1%	9/10487, 0.1%	1.000
cysteine-type endopeptidase inhibitor activity	9/10189, 0.1%	9/10487, 0.1%	1.000
transforming growth factor beta-activated receptor activity	9/10189, 0.1%	9/10487, 0.1%	1.000
O-methyltransferase activity	9/10189, 0.1%	9/10487, 0.1%	1.000
tRNA methyltransferase activity	9/10189, 0.1%	9/10487, 0.1%	1.000
phosphofructokinase activity	9/10189, 0.1%	9/10487, 0.1%	1.000
monosaccharide transmembrane transporter	9/10189, 0.1%	9/10487, 0.1%	1.000

activity			
hexose transmembrane transporter activity	9/10189, 0.1%	9/10487, 0.1%	1.000
neutral amino acid transmembrane transporter activity	9/10189, 0.1%	9/10487, 0.1%	1.000
oxidoreductase activity, oxidizing metal ions	9/10189, 0.1%	9/10487, 0.1%	1.000
transferase activity, transferring amino-acyl groups	9/10189, 0.1%	9/10487, 0.1%	1.000
hydrolase activity, acting on ether bonds	9/10189, 0.1%	9/10487, 0.1%	1.000
hydrolase activity, acting on carbon-nitrogen (but not peptide) bonds, in linear amidines	9/10189, 0.1%	9/10487, 0.1%	1.000
aldehyde-lyase activity	9/10189, 0.1%	9/10487, 0.1%	1.000
endoribonuclease activity, producing 5'-phosphomonoesters	9/10189, 0.1%	9/10487, 0.1%	1.000
Rho GTPase binding	9/10189, 0.1%	9/10487, 0.1%	1.000
kinase inhibitor activity	9/10189, 0.1%	9/10487, 0.1%	1.000
procollagen-proline dioxygenase activity	9/10189, 0.1%	9/10487, 0.1%	1.000
peptidyl-proline dioxygenase activity	9/10189, 0.1%	9/10487, 0.1%	1.000
histone methyltransferase activity	9/10189, 0.1%	9/10487, 0.1%	1.000
oxidoreductase activity, acting on NAD(P)H, oxygen as acceptor	9/10189, 0.1%	9/10487, 0.1%	1.000
exonuclease activity	30/10189, 0.3%	31/10487, 0.3%	1.000
glycoprotein binding	8/10189, 0.1%	8/10487, 0.1%	1.000
icosanoid receptor activity	8/10189, 0.1%	8/10487, 0.1%	1.000
sodium channel activity	8/10189, 0.1%	8/10487, 0.1%	1.000
cation:amino acid symporter activity	8/10189, 0.1%	8/10487, 0.1%	1.000
protein prenyltransferase activity	8/10189, 0.1%	8/10487, 0.1%	1.000
acetylgalactosaminyltransferase activity	8/10189, 0.1%	8/10487, 0.1%	1.000
fucosyltransferase activity	8/10189, 0.1%	8/10487, 0.1%	1.000
potassium-transporting ATPase activity	8/10189, 0.1%	8/10487, 0.1%	1.000
thyroid hormone receptor activator activity	8/10189, 0.1%	8/10487, 0.1%	1.000
solute:cation antiporter activity	8/10189, 0.1%	8/10487, 0.1%	1.000
anion:anion antiporter activity	8/10189, 0.1%	8/10487, 0.1%	1.000
cyclin-dependent protein serine/threonine kinase regulator activity	8/10189, 0.1%	8/10487, 0.1%	1.000
oxidoreductase activity, acting on the CH-CH group of donors, O_2 as acceptor	8/10189, 0.1%	8/10487, 0.1%	1.000
diphosphotransferase activity	8/10189, 0.1%	8/10487, 0.1%	1.000
DNA N-glycosylase activity	8/10189, 0.1%	8/10487, 0.1%	1.000
protein serine/threonine kinase inhibitor activity	8/10189, 0.1%	8/10487, 0.1%	1.000
amide transmembrane transporter activity	8/10189, 0.1%	8/10487, 0.1%	1.000

bioactive lipid receptor activity	8/10189, 0.1%	8/10487, 0.1%	1.000
transferase activity, transferring acyl groups, acyl groups converted into alkyl on transfer	8/10189, 0.1%	8/10487, 0.1%	1.000
nucleotidyltransferase activity	104/10189, 1.0%	108/10487, 1.0%	1.000
metal cluster binding	51/10189, 0.5%	53/10487, 0.5%	1.000
peptidase regulator activity	131/10189, 1.3%	136/10487, 1.3%	1.000
voltage-gated ion channel activity	27/10189, 0.3%	28/10487, 0.3%	1.000
lipid transporter activity	27/10189, 0.3%	28/10487, 0.3%	1.000
voltage-gated channel activity	27/10189, 0.3%	28/10487, 0.3%	1.000
glycosaminoglycan binding	76/10189, 0.7%	79/10487, 0.8%	1.000
DNA secondary structure binding	7/10189, 0.1%	7/10487, 0.1%	1.000
steroid hormone receptor activity	7/10189, 0.1%	7/10487, 0.1%	1.000
lactate dehydrogenase activity	7/10189, 0.1%	7/10487, 0.1%	1.000
nucleotide diphosphatase activity	7/10189, 0.1%	7/10487, 0.1%	1.000
protein geranylgeranyltransferase activity	7/10189, 0.1%	7/10487, 0.1%	1.000
sterol O-acyltransferase activity	7/10189, 0.1%	7/10487, 0.1%	1.000
succinate-CoA ligase activity	7/10189, 0.1%	7/10487, 0.1%	1.000
adrenergic receptor activity	7/10189, 0.1%	7/10487, 0.1%	1.000
water transmembrane transporter activity	7/10189, 0.1%	7/10487, 0.1%	1.000
monovalent cation:proton antiporter activity	7/10189, 0.1%	7/10487, 0.1%	1.000
microtubule binding	7/10189, 0.1%	7/10487, 0.1%	1.000
solute:proton antiporter activity	7/10189, 0.1%	7/10487, 0.1%	1.000
cation:cation antiporter activity	7/10189, 0.1%	7/10487, 0.1%	1.000
phosphatidylinositol phosphate kinase activity	7/10189, 0.1%	7/10487, 0.1%	1.000
carnitine O-acyltransferase activity	7/10189, 0.1%	7/10487, 0.1%	1.000
palmitoyltransferase activity	7/10189, 0.1%	7/10487, 0.1%	1.000
transferase activity, transferring aldehyde or ketonic groups	7/10189, 0.1%	7/10487, 0.1%	1.000
sulfurtransferase activity	7/10189, 0.1%	7/10487, 0.1%	1.000
carbon-nitrogen ligase activity, with glutamine as amido-N-donor	7/10189, 0.1%	7/10487, 0.1%	1.000
ligase activity, forming carbon-carbon bonds	7/10189, 0.1%	7/10487, 0.1%	1.000
oxidoreductase activity, acting on the CH-OH group of donors, O_2 as acceptor	7/10189, 0.1%	7/10487, 0.1%	1.000
activin-activated receptor activity	7/10189, 0.1%	7/10487, 0.1%	1.000
histone-lysine N-methyltransferase activity	7/10189, 0.1%	7/10487, 0.1%	1.000
syntaxin binding	7/10189, 0.1%	7/10487, 0.1%	1.000
low-density lipoprotein particle binding	7/10189, 0.1%	7/10487, 0.1%	1.000
adrenergic receptor binding	7/10189, 0.1%	7/10487, 0.1%	1.000
receptor serine/threonine kinase binding	7/10189, 0.1%	7/10487, 0.1%	1.000
amine binding	7/10189, 0.1%	7/10487, 0.1%	1.000

vitamin transporter activity	7/10189, 0.1%	7/10487, 0.1%	1.000
uridylyltransferase activity	7/10189, 0.1%	7/10487, 0.1%	1.000
transmembrane receptor protein serine/threonine kinase binding	7/10189, 0.1%	7/10487, 0.1%	1.000
identical protein binding	241/10189, 2.4%	250/10487, 2.4%	1.000
protein kinase regulator activity	48/10189, 0.5%	50/10487, 0.5%	1.000
cyclase activity	25/10189, 0.2%	26/10487, 0.2%	1.000
antigen binding	6/10189, 0.1%	6/10487, 0.1%	1.000
acyl-CoA dehydrogenase activity	6/10189, 0.1%	6/10487, 0.1%	1.000
dihydroorotate dehydrogenase activity	6/10189, 0.1%	6/10487, 0.1%	1.000
oligosaccharyl transferase activity	6/10189, 0.1%	6/10487, 0.1%	1.000
anaphylatoxin receptor activity	6/10189, 0.1%	6/10487, 0.1%	1.000
prostanoid receptor activity	6/10189, 0.1%	6/10487, 0.1%	1.000
melanocortin receptor activity	6/10189, 0.1%	6/10487, 0.1%	1.000
neuropeptide Y receptor activity	6/10189, 0.1%	6/10487, 0.1%	1.000
sodium:amino acid symporter activity	6/10189, 0.1%	6/10487, 0.1%	1.000
collagen binding	6/10189, 0.1%	6/10487, 0.1%	1.000
glutamate receptor activity	6/10189, 0.1%	6/10487, 0.1%	1.000
acidic amino acid transmembrane transporter activity	6/10189, 0.1%	6/10487, 0.1%	1.000
AMP binding	6/10189, 0.1%	6/10487, 0.1%	1.000
arginine N-methyltransferase activity	6/10189, 0.1%	6/10487, 0.1%	1.000
protein-arginine N-methyltransferase activity	6/10189, 0.1%	6/10487, 0.1%	1.000
CoA carboxylase activity	6/10189, 0.1%	6/10487, 0.1%	1.000
C-palmitoyltransferase activity	6/10189, 0.1%	6/10487, 0.1%	1.000
oxidoreductase activity, acting on the CH-CH group of donors, quinone or related compound as acceptor	6/10189, 0.1%	6/10487, 0.1%	1.000
oxidoreductase activity, oxidizing metal ions, oxygen as acceptor	6/10189, 0.1%	6/10487, 0.1%	1.000
immunoglobulin binding	6/10189, 0.1%	6/10487, 0.1%	1.000
protein phosphatase binding	6/10189, 0.1%	6/10487, 0.1%	1.000
mismatch repair complex binding	6/10189, 0.1%	6/10487, 0.1%	1.000
steroid dehydrogenase activity, acting on the CH-CH group of donors	6/10189, 0.1%	6/10487, 0.1%	1.000
bisphosphoglycerate phosphatase activity	6/10189, 0.1%	6/10487, 0.1%	1.000
lipase inhibitor activity	6/10189, 0.1%	6/10487, 0.1%	1.000
guanylyltransferase activity	6/10189, 0.1%	6/10487, 0.1%	1.000
lipopeptide binding	6/10189, 0.1%	6/10487, 0.1%	1.000
carbohydrate derivative transporter activity	6/10189, 0.1%	6/10487, 0.1%	1.000
phosphatase regulator activity	23/10189, 0.2%	24/10487, 0.2%	1.000
lipopolysaccharide binding	5/10189, 0.0%	5/10487, 0.0%	1.000

RNA-directed DNA polymerase activity	5/10189, 0.0%	5/10487, 0.0%	1.000
fatty acid synthase activity	5/10189, 0.0%	5/10487, 0.0%	1.000
histone deacetylase activity	5/10189, 0.0%	5/10487, 0.0%	1.000
calmodulin-dependent protein kinase activity	5/10189, 0.0%	5/10487, 0.0%	1.000
protein serine/threonine/tyrosine kinase activity	5/10189, 0.0%	5/10487, 0.0%	1.000
pyruvate dehydrogenase activity	5/10189, 0.0%	5/10487, 0.0%	1.000
prostaglandin receptor activity	5/10189, 0.0%	5/10487, 0.0%	1.000
inward rectifier potassium channel activity	5/10189, 0.0%	5/10487, 0.0%	1.000
nucleoside transmembrane transporter activity	5/10189, 0.0%	5/10487, 0.0%	1.000
S-methyltransferase activity	5/10189, 0.0%	5/10487, 0.0%	1.000
signaling pattern recognition receptor activity	5/10189, 0.0%	5/10487, 0.0%	1.000
sulfuric ester hydrolase activity	5/10189, 0.0%	5/10487, 0.0%	1.000
glucuronosyltransferase activity	5/10189, 0.0%	5/10487, 0.0%	1.000
aromatic amino acid transmembrane transporter activity	5/10189, 0.0%	5/10487, 0.0%	1.000
fatty acid ligase activity	5/10189, 0.0%	5/10487, 0.0%	1.000
galactosidase activity	5/10189, 0.0%	5/10487, 0.0%	1.000
CoA hydrolase activity	5/10189, 0.0%	5/10487, 0.0%	1.000
malate dehydrogenase activity	5/10189, 0.0%	5/10487, 0.0%	1.000
oxidoreductase activity, acting on NAD(P)H, heme protein as acceptor	5/10189, 0.0%	5/10487, 0.0%	1.000
exonuclease activity, active with either ribo- or deoxyribonucleic acids and producing 5'-phosphomonoesters	5/10189, 0.0%	5/10487, 0.0%	1.000
ether hydrolase activity	5/10189, 0.0%	5/10487, 0.0%	1.000
racemase and epimerase activity, acting on carbohydrates and derivatives	5/10189, 0.0%	5/10487, 0.0%	1.000
intramolecular oxidoreductase activity, interconverting keto- and enol-groups	5/10189, 0.0%	5/10487, 0.0%	1.000
deacetylase activity	5/10189, 0.0%	5/10487, 0.0%	1.000
mismatched DNA binding	5/10189, 0.0%	5/10487, 0.0%	1.000
alkali metal ion binding	5/10189, 0.0%	5/10487, 0.0%	1.000
chromatin DNA binding	5/10189, 0.0%	5/10487, 0.0%	1.000
protein complex scaffold	5/10189, 0.0%	5/10487, 0.0%	1.000
protein deacetylase activity	5/10189, 0.0%	5/10487, 0.0%	1.000
UDP-glucosyltransferase activity	5/10189, 0.0%	5/10487, 0.0%	1.000
pattern recognition receptor activity	5/10189, 0.0%	5/10487, 0.0%	1.000
glucosyltransferase activity	5/10189, 0.0%	5/10487, 0.0%	1.000
phosphoprotein binding	5/10189, 0.0%	5/10487, 0.0%	1.000

phosphoprotein phosphatase activity	90/10189, 0.9%	94/10487, 0.9%	1.000
oxidoreductase activity, acting on the CH-NH2 group of donors	21/10189, 0.2%	22/10487, 0.2%	1.000
nuclease activity	112/10189, 1.1%	117/10487, 1.1%	1.000
ATPase activity, coupled to transmembrane movement of ions, phosphorylative mechanism	19/10189, 0.2%	20/10487, 0.2%	1.000
oxidoreductase activity, acting on paired donors, with incorporation or reduction of molecular O_2, reduced flavin or flavoprotein as one donor, and incorporation of one atom of O_2	19/10189, 0.2%	20/10487, 0.2%	1.000
mannosyltransferase activity	4/10189, 0.0%	4/10487, 0.0%	1.000
peroxisome targeting sequence binding	4/10189, 0.0%	4/10487, 0.0%	1.000
RNA helicase activity	4/10189, 0.0%	4/10487, 0.0%	1.000
translation release factor activity	4/10189, 0.0%	4/10487, 0.0%	1.000
aldehyde dehydrogenase [NAD(P)+] activity	4/10189, 0.0%	4/10487, 0.0%	1.000
carboxypeptidase activity	4/10189, 0.0%	4/10487, 0.0%	1.000
isocitrate dehydrogenase activity	4/10189, 0.0%	4/10487, 0.0%	1.000
methylenetetrahydrofolate dehydrogenase [NAD(P)+] activity	4/10189, 0.0%	4/10487, 0.0%	1.000
exodeoxyribonuclease activity	4/10189, 0.0%	4/10487, 0.0%	1.000
alpha-mannosidase activity	4/10189, 0.0%	4/10487, 0.0%	1.000
protein tyrosine phosphatase activity	4/10189, 0.0%	4/10487, 0.0%	1.000
phospholipase inhibitor activity	4/10189, 0.0%	4/10487, 0.0%	1.000
alpha-adrenergic receptor activity	4/10189, 0.0%	4/10487, 0.0%	1.000
GDP-dissociation inhibitor activity	4/10189, 0.0%	4/10487, 0.0%	1.000
transforming growth factor beta receptor binding	4/10189, 0.0%	4/10487, 0.0%	1.000
glucose transmembrane transporter activity	4/10189, 0.0%	4/10487, 0.0%	1.000
cation:sugar symporter activity	4/10189, 0.0%	4/10487, 0.0%	1.000
translation termination factor activity	4/10189, 0.0%	4/10487, 0.0%	1.000
electron carrier activity	4/10189, 0.0%	4/10487, 0.0%	1.000
organic cation transmembrane transporter activity	4/10189, 0.0%	4/10487, 0.0%	1.000
L-amino acid transmembrane transporter activity	4/10189, 0.0%	4/10487, 0.0%	1.000
peptide transporter activity	4/10189, 0.0%	4/10487, 0.0%	1.000
anion:cation symporter activity	4/10189, 0.0%	4/10487, 0.0%	1.000
alcohol transmembrane transporter activity	4/10189, 0.0%	4/10487, 0.0%	1.000
mannosyl-oligosaccharide mannosidase activity	4/10189, 0.0%	4/10487, 0.0%	1.000

tRNA (guanine) methyltransferase activity	4/10189, 0.0%	4/10487, 0.0%	1.000
oxidoreductase activity, acting on the aldehyde or oxo group of donors, disulfide as acceptor	4/10189, 0.0%	4/10487, 0.0%	1.000
oxidoreductase activity, acting on NAD(P)H, nitrogenous group as acceptor	4/10189, 0.0%	4/10487, 0.0%	1.000
oxidoreductase activity, acting on paired donors, with oxidation of a pair of donors resulting in the reduction of molecular oxygen to two molecules of water	4/10189, 0.0%	4/10487, 0.0%	1.000
oxidoreductase activity, acting on CH or CH2 groups	4/10189, 0.0%	4/10487, 0.0%	1.000
succinyltransferase activity	4/10189, 0.0%	4/10487, 0.0%	1.000
trialkylsulfonium hydrolase activity	4/10189, 0.0%	4/10487, 0.0%	1.000
endodeoxyribonuclease activity, producing 5'-phosphomonoesters	4/10189, 0.0%	4/10487, 0.0%	1.000
exodeoxyribonuclease activity, producing 5'-phosphomonoesters	4/10189, 0.0%	4/10487, 0.0%	1.000
nucleoside-diphosphatase activity	4/10189, 0.0%	4/10487, 0.0%	1.000
NAD-dependent histone deacetylase activity	4/10189, 0.0%	4/10487, 0.0%	1.000
cyclohydrolase activity	4/10189, 0.0%	4/10487, 0.0%	1.000
apolipoprotein receptor activity	4/10189, 0.0%	4/10487, 0.0%	1.000
heparan sulfate sulfotransferase activity	4/10189, 0.0%	4/10487, 0.0%	1.000
NAD-dependent protein deacetylase activity	4/10189, 0.0%	4/10487, 0.0%	1.000
histone kinase activity	4/10189, 0.0%	4/10487, 0.0%	1.000
proteoglycan binding	4/10189, 0.0%	4/10487, 0.0%	1.000
protein phosphorylated amino acid binding	4/10189, 0.0%	4/10487, 0.0%	1.000
nucleoside-triphosphate diphosphatase activity	4/10189, 0.0%	4/10487, 0.0%	1.000
3',5'-cyclic-GMP phosphodiesterase activity	4/10189, 0.0%	4/10487, 0.0%	1.000
galactose 3-O-sulfotransferase activity	4/10189, 0.0%	4/10487, 0.0%	1.000
cytidylyltransferase activity	4/10189, 0.0%	4/10487, 0.0%	1.000
organic hydroxy compound transmembrane transporter activity	4/10189, 0.0%	4/10487, 0.0%	1.000
tRNA-specific ribonuclease activity	18/10189, 0.2%	19/10487, 0.2%	1.000
metallopeptidase activity	18/10189, 0.2%	19/10487, 0.2%	1.000
tumor necrosis factor receptor superfamily binding	18/10189, 0.2%	19/10487, 0.2%	1.000
cytokine receptor binding	81/10189, 0.8%	85/10487, 0.8%	1.000
hydrogen ion transmembrane transporter activity	36/10189, 0.4%	38/10487, 0.4%	1.000
small conjugating protein binding	36/10189, 0.4%	38/10487, 0.4%	1.000
voltage-gated cation channel activity	17/10189, 0.2%	18/10487, 0.2%	1.000

enzyme activator activity	129/10189, 1.3%	135/10487, 1.3%	1.000
nucleic acid binding	1473/10189, 14.5%	1524/10487, 14.5%	1.000
iron-sulfur cluster binding	35/10189, 0.3%	37/10487, 0.4%	1.000
oxidized base lesion DNA N-glycosylase activity	3/10189, 0.0%	3/10487, 0.0%	1.000
angiotensin receptor activity	3/10189, 0.0%	3/10487, 0.0%	1.000
purinergic nucleotide receptor activity	3/10189, 0.0%	3/10487, 0.0%	1.000
lipid kinase activity	3/10189, 0.0%	3/10487, 0.0%	1.000
single-stranded RNA binding	3/10189, 0.0%	3/10487, 0.0%	1.000
DNA-(apurinic or apyrimidinic site) lyase activity	3/10189, 0.0%	3/10487, 0.0%	1.000
DNA topoisomerase activity	3/10189, 0.0%	3/10487, 0.0%	1.000
branched-chain-amino-acid transaminase activity	3/10189, 0.0%	3/10487, 0.0%	1.000
phosphoglycerate mutase activity	3/10189, 0.0%	3/10487, 0.0%	1.000
cyclic nucleotide-dependent protein kinase activity	3/10189, 0.0%	3/10487, 0.0%	1.000
MAP kinase activity	3/10189, 0.0%	3/10487, 0.0%	1.000
protein serine/threonine phosphatase activity	3/10189, 0.0%	3/10487, 0.0%	1.000
sulfate adenylyltransferase activity	3/10189, 0.0%	3/10487, 0.0%	1.000
uracil DNA N-glycosylase activity	3/10189, 0.0%	3/10487, 0.0%	1.000
interleukin-1 receptor activity	3/10189, 0.0%	3/10487, 0.0%	1.000
opioid receptor activity	3/10189, 0.0%	3/10487, 0.0%	1.000
transforming growth factor beta receptor, cytoplasmic mediator activity	3/10189, 0.0%	3/10487, 0.0%	1.000
vascular endothelial growth factor receptor binding	3/10189, 0.0%	3/10487, 0.0%	1.000
neurotransmitter:sodium symporter activity	3/10189, 0.0%	3/10487, 0.0%	1.000
iron ion transmembrane transporter activity	3/10189, 0.0%	3/10487, 0.0%	1.000
alcohol dehydrogenase (NADP+) activity	3/10189, 0.0%	3/10487, 0.0%	1.000
mRNA methyltransferase activity	3/10189, 0.0%	3/10487, 0.0%	1.000
RNA guanylyltransferase activity	3/10189, 0.0%	3/10487, 0.0%	1.000
ion channel inhibitor activity	3/10189, 0.0%	3/10487, 0.0%	1.000
tetracycline transporter activity	3/10189, 0.0%	3/10487, 0.0%	1.000
monoamine transmembrane transporter activity	3/10189, 0.0%	3/10487, 0.0%	1.000
oxidized purine nucleobase lesion DNA N-glycosylase activity	3/10189, 0.0%	3/10487, 0.0%	1.000
NAD(P)+ transhydrogenase activity	3/10189, 0.0%	3/10487, 0.0%	1.000
bis(5'-nucleosyl)-tetraphosphatase activity	3/10189, 0.0%	3/10487, 0.0%	1.000
mannose-phosphate guanylyltransferase activity	3/10189, 0.0%	3/10487, 0.0%	1.000

phosphate ion transmembrane transporter activity	3/10189, 0.0%	3/10487, 0.0%	1.000
polyol transmembrane transporter activity	3/10189, 0.0%	3/10487, 0.0%	1.000
calcium-activated potassium channel activity	3/10189, 0.0%	3/10487, 0.0%	1.000
acetylcholine receptor activity	3/10189, 0.0%	3/10487, 0.0%	1.000
glucosidase activity	3/10189, 0.0%	3/10487, 0.0%	1.000
acyl-CoA desaturase activity	3/10189, 0.0%	3/10487, 0.0%	1.000
O-acetyltransferase activity	3/10189, 0.0%	3/10487, 0.0%	1.000
C-acetyltransferase activity	3/10189, 0.0%	3/10487, 0.0%	1.000
oxidoreductase activity, acting on the CH-NH group of donors, quinone or similar compound as acceptor	3/10189, 0.0%	3/10487, 0.0%	1.000
oxidoreductase activity, acting on NAD(P)H, NAD(P) as acceptor	3/10189, 0.0%	3/10487, 0.0%	1.000
oxidoreductase activity, acting on paired donors, with incorporation or reduction of molecular O_2, reduced pteridine as one donor, and incorporation of one atom of O_2	3/10189, 0.0%	3/10487, 0.0%	1.000
oxidoreductase activity, oxidizing metal ions, NAD or NADP as acceptor	3/10189, 0.0%	3/10487, 0.0%	1.000
N-succinyltransferase activity	3/10189, 0.0%	3/10487, 0.0%	1.000
phosphotransferase activity, carboxyl group as acceptor	3/10189, 0.0%	3/10487, 0.0%	1.000
hydrolase activity, acting on acid carbon-carbon bonds	3/10189, 0.0%	3/10487, 0.0%	1.000
hydrolase activity, acting on acid carbon-carbon bonds, in ketonic substances	3/10189, 0.0%	3/10487, 0.0%	1.000
amidine-lyase activity	3/10189, 0.0%	3/10487, 0.0%	1.000
endodeoxyribonuclease activity, producing 3'-phosphomonoesters	3/10189, 0.0%	3/10487, 0.0%	1.000
G-protein coupled acetylcholine receptor activity	3/10189, 0.0%	3/10487, 0.0%	1.000
SAP kinase activity	3/10189, 0.0%	3/10487, 0.0%	1.000
alpha-sialidase activity	3/10189, 0.0%	3/10487, 0.0%	1.000
CDP-alcohol phosphatidyltransferase activity	3/10189, 0.0%	3/10487, 0.0%	1.000
toxin transporter activity	3/10189, 0.0%	3/10487, 0.0%	1.000
immunoglobulin receptor activity	3/10189, 0.0%	3/10487, 0.0%	1.000
interferon binding	3/10189, 0.0%	3/10487, 0.0%	1.000
translation initiation factor binding	3/10189, 0.0%	3/10487, 0.0%	1.000
polyubiquitin binding	3/10189, 0.0%	3/10487, 0.0%	1.000
bombesin receptor binding	3/10189, 0.0%	3/10487, 0.0%	1.000

DNA insertion or deletion binding	3/10189, 0.0%	3/10487, 0.0%	1.000
apolipoprotein binding	3/10189, 0.0%	3/10487, 0.0%	1.000
peptide N-acetyltransferase activity	3/10189, 0.0%	3/10487, 0.0%	1.000
phosphatidylinositol bisphosphate phosphatase activity	3/10189, 0.0%	3/10487, 0.0%	1.000
histone threonine kinase activity	3/10189, 0.0%	3/10487, 0.0%	1.000
purinergic receptor activity	3/10189, 0.0%	3/10487, 0.0%	1.000
antibiotic transporter activity	3/10189, 0.0%	3/10487, 0.0%	1.000
G-protein coupled purinergic nucleotide receptor activity	3/10189, 0.0%	3/10487, 0.0%	1.000
quinone binding	3/10189, 0.0%	3/10487, 0.0%	1.000
UTP-monosaccharide-1-phosphate uridylyltransferase activity	3/10189, 0.0%	3/10487, 0.0%	1.000
carboxylic ester hydrolase activity	3/10189, 0.0%	3/10487, 0.0%	1.000
phosphatidylinositol phosphate phosphatase activity	3/10189, 0.0%	3/10487, 0.0%	1.000
lipase activator activity	3/10189, 0.0%	3/10487, 0.0%	1.000
peptidyl-lysine 5-dioxygenase activity	3/10189, 0.0%	3/10487, 0.0%	1.000
drug transporter activity	3/10189, 0.0%	3/10487, 0.0%	1.000
deaminated base DNA N-glycosylase activity	3/10189, 0.0%	3/10487, 0.0%	1.000
double-stranded DNA binding	16/10189, 0.2%	17/10487, 0.2%	1.000
protein transporter activity	16/10189, 0.2%	17/10487, 0.2%	1.000
hydrolase activity, acting on carbon-nitrogen (but not peptide) bonds, in cyclic amidines	16/10189, 0.2%	17/10487, 0.2%	1.000
calcium channel activity	15/10189, 0.1%	16/10487, 0.2%	1.000
calcium ion transmembrane transporter activity	15/10189, 0.1%	16/10487, 0.2%	1.000
oxidoreductase activity, acting on the CH-NH2 group of donors, O_2 as acceptor	15/10189, 0.1%	16/10487, 0.2%	1.000
divalent inorganic cation transmembrane transporter activity	15/10189, 0.1%	16/10487, 0.2%	1.000
protein transmembrane transporter activity	14/10189, 0.1%	15/10487, 0.1%	1.000
macromolecule transmembrane transporter activity	14/10189, 0.1%	15/10487, 0.1%	1.000
RNA cap binding	2/10189, 0.0%	2/10487, 0.0%	1.000
damaged DNA binding	2/10189, 0.0%	2/10487, 0.0%	1.000
11-beta-hydroxysteroid dehydrogenase [NAD(P)] activity	2/10189, 0.0%	2/10487, 0.0%	1.000
DNA-directed RNA polymerase activity	2/10189, 0.0%	2/10487, 0.0%	1.000
glycogen debranching enzyme activity	2/10189, 0.0%	2/10487, 0.0%	1.000
farnesyltranstransferase activity	2/10189, 0.0%	2/10487, 0.0%	1.000

exoribonuclease activity	2/10189, 0.0%	2/10487, 0.0%	1.000
alpha-1,4-glucosidase activity	2/10189, 0.0%	2/10487, 0.0%	1.000
phosphoenolpyruvate carboxykinase activity	2/10189, 0.0%	2/10487, 0.0%	1.000
phospholipase A2 activity	2/10189, 0.0%	2/10487, 0.0%	1.000
phosphorylase activity	2/10189, 0.0%	2/10487, 0.0%	1.000
saccharopine dehydrogenase activity	2/10189, 0.0%	2/10487, 0.0%	1.000
ciliary neurotrophic factor receptor activity	2/10189, 0.0%	2/10487, 0.0%	1.000
beta-adrenergic receptor activity	2/10189, 0.0%	2/10487, 0.0%	1.000
leukotriene receptor activity	2/10189, 0.0%	2/10487, 0.0%	1.000
death receptor activity	2/10189, 0.0%	2/10487, 0.0%	1.000
nuclear export signal receptor activity	2/10189, 0.0%	2/10487, 0.0%	1.000
fibroblast growth factor receptor binding	2/10189, 0.0%	2/10487, 0.0%	1.000
intracellular cyclic nucleotide activated cation channel activity	2/10189, 0.0%	2/10487, 0.0%	1.000
inhibitory extracellular ligand-gated ion channel activity	2/10189, 0.0%	2/10487, 0.0%	1.000
L-glutamate transmembrane transporter activity	2/10189, 0.0%	2/10487, 0.0%	1.000
insulin-like growth factor binding	2/10189, 0.0%	2/10487, 0.0%	1.000
high-density lipoprotein particle binding	2/10189, 0.0%	2/10487, 0.0%	1.000
5'-3' exonuclease activity	2/10189, 0.0%	2/10487, 0.0%	1.000
CoA-transferase activity	2/10189, 0.0%	2/10487, 0.0%	1.000
histone-arginine N-methyltransferase activity	2/10189, 0.0%	2/10487, 0.0%	1.000
basic amino acid transmembrane transporter activity	2/10189, 0.0%	2/10487, 0.0%	1.000
oligopeptide transporter activity	2/10189, 0.0%	2/10487, 0.0%	1.000
nucleobase transmembrane transporter activity	2/10189, 0.0%	2/10487, 0.0%	1.000
secondary active oligopeptide transmembrane transporter activity	2/10189, 0.0%	2/10487, 0.0%	1.000
peptide-transporting ATPase activity	2/10189, 0.0%	2/10487, 0.0%	1.000
aspartate oxidase activity	2/10189, 0.0%	2/10487, 0.0%	1.000
ammonia ligase activity	2/10189, 0.0%	2/10487, 0.0%	1.000
S-acyltransferase activity	2/10189, 0.0%	2/10487, 0.0%	1.000
tRNA (adenine) methyltransferase activity	2/10189, 0.0%	2/10487, 0.0%	1.000
oxidoreductase activity, acting on a sulfur group of donors, NAD(P) as acceptor	2/10189, 0.0%	2/10487, 0.0%	1.000
oxidoreductase activity, acting on a sulfur group of donors, O_2 as acceptor	2/10189, 0.0%	2/10487, 0.0%	1.000
oxidoreductase activity, acting on paired donors, with incorporation or reduction of molecular O_2, reduced ascorbate as one	2/10189, 0.0%	2/10487, 0.0%	1.000

donor, and incorporation of one atom of O_2			
oxidoreductase activity, acting on paired donors, with incorporation or reduction of molecular O_2, another compound as one donor, and incorporation of one atom of O_2	2/10189, 0.0%	2/10487, 0.0%	1.000
oxidoreductase activity, acting on CH or CH2 groups, NAD or NADP as acceptor	2/10189, 0.0%	2/10487, 0.0%	1.000
oxidoreductase activity, acting on CH or CH2 groups, O_2 as acceptor	2/10189, 0.0%	2/10487, 0.0%	1.000
oxidoreductase activity, acting on CH or CH2 groups, disulfide as acceptor	2/10189, 0.0%	2/10487, 0.0%	1.000
oxo-acid-lyase activity	2/10189, 0.0%	2/10487, 0.0%	1.000
acid-ammonia (or amide) ligase activity	2/10189, 0.0%	2/10487, 0.0%	1.000
ligase activity, forming phosphoric ester bonds	2/10189, 0.0%	2/10487, 0.0%	1.000
oxidoreductase activity, acting on the CH-OH group of donors, quinone or similar compound as acceptor	2/10189, 0.0%	2/10487, 0.0%	1.000
snRNA binding	2/10189, 0.0%	2/10487, 0.0%	1.000
sodium:dicarboxylate symporter activity	2/10189, 0.0%	2/10487, 0.0%	1.000
interleukin-12 binding	2/10189, 0.0%	2/10487, 0.0%	1.000
alpha-(1,2)-fucosyltransferase activity	2/10189, 0.0%	2/10487, 0.0%	1.000
opioid receptor binding	2/10189, 0.0%	2/10487, 0.0%	1.000
endothelial differentiation G-protein coupled receptor binding	2/10189, 0.0%	2/10487, 0.0%	1.000
single base insertion or deletion binding	2/10189, 0.0%	2/10487, 0.0%	1.000
oxidized DNA binding	2/10189, 0.0%	2/10487, 0.0%	1.000
demethylase activity	2/10189, 0.0%	2/10487, 0.0%	1.000
histone demethylase activity	2/10189, 0.0%	2/10487, 0.0%	1.000
oxoglutarate dehydrogenase [NAD(P)+] activity	2/10189, 0.0%	2/10487, 0.0%	1.000
glutamate receptor binding	2/10189, 0.0%	2/10487, 0.0%	1.000
5'-3' exodeoxyribonuclease activity	2/10189, 0.0%	2/10487, 0.0%	1.000
oligopeptide transmembrane transporter activity	2/10189, 0.0%	2/10487, 0.0%	1.000
neurotrophin binding	2/10189, 0.0%	2/10487, 0.0%	1.000
laminin binding	2/10189, 0.0%	2/10487, 0.0%	1.000
cyclic nucleotide-gated ion channel activity	2/10189, 0.0%	2/10487, 0.0%	1.000
alpha-(1->6)-fucosyltransferase activity	2/10189, 0.0%	2/10487, 0.0%	1.000
TAP binding	2/10189, 0.0%	2/10487, 0.0%	1.000
dopamine receptor binding	2/10189, 0.0%	2/10487, 0.0%	1.000
extracellular matrix binding	2/10189, 0.0%	2/10487, 0.0%	1.000
cofactor transporter activity	2/10189, 0.0%	2/10487, 0.0%	1.000

BH domain binding	2/10189, 0.0%	2/10487, 0.0%	1.000
inositol tetrakisphosphate kinase activity	2/10189, 0.0%	2/10487, 0.0%	1.000
inositol phosphate phosphatase activity	2/10189, 0.0%	2/10487, 0.0%	1.000
ligase regulator activity	2/10189, 0.0%	2/10487, 0.0%	1.000
L-tyrosine aminotransferase activity	2/10189, 0.0%	2/10487, 0.0%	1.000
alpha-glucosidase activity	2/10189, 0.0%	2/10487, 0.0%	1.000
endonuclease activity	47/10189, 0.5%	50/10487, 0.5%	1.000
SNARE binding	12/10189, 0.1%	13/10487, 0.1%	1.000
peroxidase activity	12/10189, 0.1%	13/10487, 0.1%	1.000
amine transmembrane transporter activity	12/10189, 0.1%	13/10487, 0.1%	1.000
oxidoreductase activity, acting on peroxide as acceptor	12/10189, 0.1%	13/10487, 0.1%	1.000
isoprenoid binding	12/10189, 0.1%	13/10487, 0.1%	1.000
protein binding, bridging	12/10189, 0.1%	13/10487, 0.1%	1.000
binding, bridging	12/10189, 0.1%	13/10487, 0.1%	1.000
protein domain specific binding	134/10189, 1.3%	141/10487, 1.3%	1.000
G-protein coupled receptor binding	45/10189, 0.4%	48/10487, 0.5%	1.000
carboxylic acid binding	45/10189, 0.4%	48/10487, 0.5%	1.000
organic acid binding	45/10189, 0.4%	48/10487, 0.5%	1.000
retinoid binding	11/10189, 0.1%	12/10487, 0.1%	1.000
oxidoreductase activity, acting on the CH-NH group of donors	25/10189, 0.2%	27/10487, 0.3%	1.000
iron ion binding	177/10189, 1.7%	186/10487, 1.8%	1.000
phospholipid transporter activity	10/10189, 0.1%	11/10487, 0.1%	1.000
inositol trisphosphate kinase activity	10/10189, 0.1%	11/10487, 0.1%	1.000
acylglycerol O-acyltransferase activity	9/10189, 0.1%	10/10487, 0.1%	1.000
intramolecular oxidoreductase activity, transposing C=C bonds	9/10189, 0.1%	10/10487, 0.1%	1.000
hormone binding	9/10189, 0.1%	10/10487, 0.1%	1.000
translation regulator activity	9/10189, 0.1%	10/10487, 0.1%	1.000
lipoprotein particle receptor binding	9/10189, 0.1%	10/10487, 0.1%	1.000
isomerase activity	141/10189, 1.4%	149/10487, 1.4%	1.000
carbon-nitrogen lyase activity	8/10189, 0.1%	9/10487, 0.1%	1.000
kinase activator activity	8/10189, 0.1%	9/10487, 0.1%	1.000
phosphatase inhibitor activity	8/10189, 0.1%	9/10487, 0.1%	1.000
protein kinase activator activity	8/10189, 0.1%	9/10487, 0.1%	1.000
growth factor binding	34/10189, 0.3%	37/10487, 0.4%	1.000
neuropeptide receptor binding	7/10189, 0.1%	8/10487, 0.1%	1.000
oxidoreductase activity, acting on NAD(P)H	48/10189, 0.5%	52/10487, 0.5%	1.000
ubiquitin binding	15/10189, 0.1%	17/10487, 0.2%	1.000
3'-5' exonuclease activity	5/10189, 0.0%	6/10487, 0.1%	1.000
receptor binding	512/10189, 5.0%	536/10487, 5.1%	1.000
cis-trans isomerase activity	59/10189, 0.6%	64/10487, 0.6%	1.000

ribonuclease activity	41/10189, 0.4%	45/10487, 0.4%	1.000
oxidoreductase activity	585/10189, 5.7%	612/10487, 5.8%	1.000
receptor regulator activity	13/10189, 0.1%	15/10487, 0.1%	1.000
ribonucleoprotein complex binding	25/10189, 0.2%	28/10487, 0.3%	1.000
voltage-gated calcium channel activity	4/10189, 0.0%	5/10487, 0.0%	1.000
protein-hormone receptor activity	4/10189, 0.0%	5/10487, 0.0%	1.000
ammonia-lyase activity	4/10189, 0.0%	5/10487, 0.0%	1.000
peptide hormone binding	4/10189, 0.0%	5/10487, 0.0%	1.000
clathrin binding	4/10189, 0.0%	5/10487, 0.0%	1.000
oxidoreductase activity, acting on NAD(P)H, quinone as acceptor	24/10189, 0.2%	27/10487, 0.3%	1.000
oxidoreductase activity, acting on a sulfur group of donors	37/10189, 0.4%	41/10487, 0.4%	1.000
cytokine activity	34/10189, 0.3%	38/10487, 0.4%	1.000
RNA polymerase activity	34/10189, 0.3%	38/10487, 0.4%	1.000
hormone activity	10/10189, 0.1%	12/10487, 0.1%	1.000
oxidoreductase activity, acting on a sulfur group of donors, disulfide as acceptor	10/10189, 0.1%	12/10487, 0.1%	1.000
racemase and epimerase activity	10/10189, 0.1%	12/10487, 0.1%	1.000
endoribonuclease activity	20/10189, 0.2%	23/10487, 0.2%	1.000
NADH dehydrogenase activity	18/10189, 0.2%	21/10487, 0.2%	1.000
calcium channel regulator activity	2/10189, 0.0%	3/10487, 0.0%	1.000
copper ion binding	2/10189, 0.0%	3/10487, 0.0%	1.000
melanocortin receptor binding	2/10189, 0.0%	3/10487, 0.0%	1.000
NADH dehydrogenase (quinone) activity	17/10189, 0.2%	20/10487, 0.2%	1.000
channel regulator activity	16/10189, 0.2%	19/10487, 0.2%	1.000
amino acid binding	16/10189, 0.2%	19/10487, 0.2%	1.000
fibroblast growth factor binding	6/10189, 0.1%	8/10487, 0.1%	1.000
cytokine binding	6/10189, 0.1%	8/10487, 0.1%	1.000
endonuclease activity, active with either ribo- or deoxyribonucleic acids and producing 3'-phosphomonoesters	13/10189, 0.1%	16/10487, 0.2%	1.000
disulfide oxidoreductase activity	21/10189, 0.2%	25/10487, 0.2%	1.000
endoribonuclease activity, producing 3'-phosphomonoesters	10/10189, 0.1%	13/10487, 0.1%	1.000
nucleobase-containing compound kinase activity	10/10189, 0.1%	13/10487, 0.1%	1.000
channel inhibitor activity	3/10189, 0.0%	5/10487, 0.0%	1.000
nucleoside kinase activity	7/10189, 0.1%	10/10487, 0.1%	1.000
myosin binding	2/10189, 0.0%	4/10487, 0.0%	1.000
protein tyrosine/serine/threonine phosphatase activity	10/10189, 0.1%	14/10487, 0.1%	1.000
structural molecule activity	333/10189, 3.3%	374/10487, 3.6%	1.000
heme-copper terminal oxidase activity	24/10189, 0.2%	36/10487, 0.3%	1.000

Supplementary Table S7

Quantative real-time PCR primers for target miRNAs

	Forward	Reverse
**Mir-93**	**AACTGTTTGCAGAGGAAACTGA**	**GTGACTGGAGTTCAGACGTGTGCTCTTCCGATCT**
**Mir-20**	**TCCCTGAGACCCTAACTTGTGAT**	
**Mir-81**	**TAGCAGCGGGAACAGTACTGCAG**	
**Mir-21-5p**	**TAGCTTATCAGACTGATGTTGAC**	
**Mir-127-3p**	**TCGGATCCGTCTGAGCTTGG**	
**Mir-199b-5p**	**CCCAGTGTTTAGACTATCTGTTC**	
**b-actin**	**cctgacggtcatgtaattaccg**	**tctcaccgataccaacagct**
